# The genome of the white-rot fungus *Pycnoporus cinnabarinus*: a basidiomycete model with a versatile arsenal for lignocellulosic biomass breakdown

**DOI:** 10.1186/1471-2164-15-486

**Published:** 2014-06-18

**Authors:** Anthony Levasseur, Anne Lomascolo, Olivier Chabrol, Francisco J Ruiz-Dueñas, Eva Boukhris-Uzan, François Piumi, Ursula Kües, Arthur F J Ram, Claude Murat, Mireille Haon, Isabelle Benoit, Yonathan Arfi, Didier Chevret, Elodie Drula, Min Jin Kwon, Philippe Gouret, Laurence Lesage-Meessen, Vincent Lombard, Jérôme Mariette, Céline Noirot, Joohae Park, Aleksandrina Patyshakuliyeva, Jean Claude Sigoillot, Ad Wiebenga, Han A B Wösten, Francis Martin, Pedro M Coutinho, Ronald P de Vries, Angel T Martínez, Christophe Klopp, Pierre Pontarotti, Bernard Henrissat, Eric Record

**Affiliations:** INRA, UMR1163 Biotechnologie des Champignons Filamenteux, Aix-Marseille Université, Polytech Marseille, 163 avenue de Luminy, CP 925, 13288 Marseille Cedex 09, France; Aix-Marseille Université, INRA, UMR1163 Biotechnologie des Champignons Filamenteux, Faculté des Sciences de Luminy-Polytech, CP 925, 13288 Marseille Cedex 09, France; Centrale Marseille, I2M, UMR7373, Aix-Marseille Université, CNRS, FR 4213 - FR Eccorev 3098, équipe EBM, 13331 Marseille, France; Centro de Investigaciones Biológicas (CIB), CSIC, Ramiro de Maeztu 9, E-28040 Madrid, Spain; CNRS ISM2 UMR 7353, Aix-Marseille Université, Campus Scientifique de Saint Jérôme avenue Escadrille Normandie-Niemen, case 341, 13397 Marseille Cedex 20, France; Molecular Wood Biotechnology and Technical Mycology, Büsgen-Instit ute, Georg-August-University, 37077 Göttingen, Germany; Department of Molecular Microbiology and Biotechnology, Institute of Biology Leiden, Leiden University, Sylviusweg 72, 2333 BE Leiden, The Netherlands; INRA, UMR 1136 INRA Université de Lorraine ‘Interactions Arbres-Microorganismes’, Labex ARBRE, FR EFABA, 54280 Champenoux, France; Fungal Physiology, CBS-KNAW Fungal Biodiversity Centre, P.O. Box 85167, 3508 AD Utrecht, The Netherlands; Faculty of Biological Chemistry, The Weizmann Institute of Science, 234 Herzl Street, Rehovot, 7610001 Israël; UMR1319 Micalis, Plateforme d’Analyse Protéomique de Paris Sud-Ouest, INRA, 78352 Jouy-en-Josas, France; Architecture et Fonction des Macromolécules Biologiques, Aix-Marseille Université, 13288 Marseille, France; CNRS UMR 7257, Centre National de la Recherche Scientifique, 13288 Marseille, France; Plateforme bioinformatique Genotoul, UR875 Biométrie et Intelligence Artificielle, INRA, 31326 Castanet-Tolosan, France; Department of Microbiology, Kluyver Centre for Genomics of Industrial Fermentation – Utrecht University, Padualaan 8, 3584 CH Utrecht, The Netherlands

**Keywords:** *Pycnoporus cinnabarinus*, Genome annotation, CAZy, Auxiliary activities, Oxidoreductase, White-rot fungi, Lignocellulose

## Abstract

**Background:**

Saprophytic filamentous fungi are ubiquitous micro-organisms that play an essential role in photosynthetic carbon recycling. The wood-decayer *Pycnoporus cinnabarinus* is a model fungus for the study of plant cell wall decomposition and is used for a number of applications in green and white biotechnology.

**Results:**

The 33.6 megabase genome of *P. cinnabarinus* was sequenced and assembled, and the 10,442 predicted genes were functionally annotated using a phylogenomic procedure. In-depth analyses were carried out for the numerous enzyme families involved in lignocellulosic biomass breakdown, for protein secretion and glycosylation pathways, and for mating type. The *P. cinnabarinus* genome sequence revealed a consistent repertoire of genes shared with wood-decaying basidiomycetes. *P. cinnabarinus* is thus fully equipped with the classical families involved in cellulose and hemicellulose degradation, whereas its pectinolytic repertoire appears relatively limited. In addition, *P. cinnabarinus* possesses a complete versatile enzymatic arsenal for lignin breakdown. We identified several genes encoding members of the three ligninolytic peroxidase types, namely lignin peroxidase, manganese peroxidase and versatile peroxidase. Comparative genome analyses were performed in fungi displaying different nutritional strategies (white-rot and brown-rot modes of decay). *P. cinnabarinus* presents a typical distribution of all the specific families found in the white-rot life style. Growth profiling of *P. cinnabarinus* was performed on 35 carbon sources including simple and complex substrates to study substrate utilization and preferences. *P. cinnabarinus* grew faster on crude plant substrates than on pure, mono- or polysaccharide substrates. Finally, proteomic analyses were conducted from liquid and solid-state fermentation to analyze the composition of the secretomes corresponding to growth on different substrates. The distribution of lignocellulolytic enzymes in the secretomes was strongly dependent on growth conditions, especially for lytic polysaccharide mono-oxygenases.

**Conclusions:**

With its available genome sequence, *P. cinnabarinus* is now an outstanding model system for the study of the enzyme machinery involved in the degradation or transformation of lignocellulosic biomass.

**Electronic supplementary material:**

The online version of this article (doi:10.1186/1471-2164-15-486) contains supplementary material, which is available to authorized users.

## Background

Filamentous fungi are a source of powerful enzymes for plant biomass breakdown and/or hydrolysis in green and white biotechnology, especially biorefining [1]. The enzymatic modification of lignin-derived aromatic compounds is of strategic importance both for biomass valorization of the other plant-cell-wall compounds in the green chemistry sector and for the biotransformation of these aromatic compounds into high-value products (foods, cosmetics and pharmaceuticals) or industrial compounds (surfactants, adhesives and biomaterials).

The proportions of the constituent polymers of plant cell walls, *i.e.* cellulose, hemicelluloses, pectin and lignins, fluctuates with botanical origin, tissue, and age of the plant. In response to the structural complexity and heterogeneity of the different plant cell wall polymers, saprophytic fungi produce a complex arsenal of enzymes to gain access to the carbon source. Lignocellulolytic fungi have traditionally been classified into three main fungal groups according to the appearance of the plant material remaining after decomposition [2]. Soft-rot fungi partially degrade plant polysaccharides by mobilizing cellulases and hemicellulases, and cause wood softening [3]. In contrast, brown-rot fungi such as *Postia placenta* produce enzymes involved in extracellular generation of Fenton’s reagent, where hydroxyl radicals resulting from the reaction between Fe(II) and hydrogen peroxide may ultimately cause cellulose depolymerization [4]. Lignin is apparently only slightly modified in this process, and remains as a crumbly, brownish material. Unlike the above two groups, white-rot fungi are the only organisms able to effectively degrade lignin, in a process called enzymatic combustion [5] where peroxidases cooperate with other oxidoreductases [6]. The decayed wood resulting from attack by white-rot fungi becomes white and stringy. For selective white-rot fungi, the white color is caused by rapid hemicellulose and lignin breakdown of the cell-wall constituents, followed later by cellulose degradation [7].

The white-rot fungus *Pycnoporus* is very efficient at completely degrading lignin [8]. The *Pycnoporus* genus belongs to the phylum Basidiomycota, class Agaricomycetes, order Polyporales, family Polyporaceae. The genus *Pycnoporus* is divided into four species with different geographic origins: *P. cinnabarinus* is widely distributed especially in the Northern hemisphere, *P. coccineus* in countries bordering the Indian and Pacific Oceans, *P. sanguineus* in the tropics and subtropics of both hemispheres, and *P. puniceus*, a rare species found in Africa, India, Malaysia and New Caledonia. *Pycnoporus* mycelia and fruiting bodies are characterized by red-to-orange pigmentation due to phenoxazinone pigments including cinnabarin, tramesanguin and cinnabarinic acid [9]. *P. cinnabarinus* is a heterothallic homobasidiomycete with a tetrapolar mating system. Its life cycle includes a short monokaryotic stage after spore germination, followed after mating by an indefinite dikaryotic stage where karyogamy and meiosis can take place. The fungus is able to produce fruiting body structures and to generate stable monokaryotic cell-lines amenable to genetic improvement by formal genetics and genetic engineering, e.g. development of expression systems for high- level ligninase production [10].

*P. cinnabarinus* has a large array of copper- and iron-containing metalloenzymes involved in transforming plant-cell-wall aromatics [11, 12] and harbors original metabolic pathways involved in functionalizing these cell-wall aromatics to yield high-added-value compounds such as aromas and antioxidants [13, 14]. *P. cinnabarinus* is listed as a food- and cosmetic-grade microorganism [15]. Among enzymes involved in lignin degradation, *P. cinnabarinus* is known to produce high-redox-potential laccase as the predominant enzyme at very high levels of up to 1 g per liter [16, 17]. The potential of *Pycnoporus* fungi lies in their laccases which find a variety of applications, such as bioconversion of agricultural by-products and raw plant materials into valuable products, biopulping and biobleaching paper pulp [18–22], dye bleaching in the textile and dye industries [23–25], wastewater treatment [26–28], removal of phenolic compounds in beverages [29], biosensor and biofuel cell construction [30], and producing substances of pharmaceutical importance [31].

All studies performed over the last decade support the *Pycnoporus* genus as a strong contender for green and white biotechnology applications. Here, we describe the sequencing and annotation of the *P. cinnabarinus* monokaryotic cell-line BRFM137 genome, its growth profiling and its secretome analyses under different culture conditions. Lignocellulolytic repertoires of *P. cinnabarinus* are highlighted and compared with other fungal counterparts. *P. cinnabarinus* emerges as a versatile white-rot fungus for biotechnological applications.

## Methods

### Strain, DNA preparation and culture conditions

Monokaryotic strain *P. cinnabarinus* BRFM137 [9] was obtained from the International Centre of Microbial Resources dedicated to Filamentous Fungi (CIRM-CF, Marseille, France, http://cirm.esil.univ-mrs.fr/crbmarseille/pages/index_mizenpage.php). Genomic DNA was isolated from ground mycelia powder as described in Lomascolo *et al.* [32], and a roughly 180 μg sample was sent to GATC Biotech AG (Constanz, Germany) for genome sequencing.

For construction of the cDNA library, *P. cinnabarinus* BRFM137 was grown as described in Lomascolo *et al.* [17] with five types of substrate: *(i)* 20 g/L maltose, *(ii)* 20 g/l maltose and 0.1 g/l ferulic acid, *(iii)* 5 g/l maltose and 15 g/l oat spelt xylan (Sigma), *(iv)* 5 g/l maltose and 15 g/l autoclaved maize bran (ARD, Pomacle, France) and *(v)* 5 g/l maltose and 15 g/l Avicel cellulose (Sigma). The other constituents of the medium were: diammonium tartrate (1.84 g/l); disodium tartrate (2.3 g/l); KH_2_PO_4_ (1.33 g/l); CaCl_2_ · 2H_2_O (0.1 g/l); MgSO_4_ · 7H_2_O (0.5 g/l); FeSO_4_ · 7H_2_O (0.07 g/l); ZnSO_4_ · 7H_2_O (0.046 g/l); MnSO_4_ · H_2_O (0.035 g/l); CuSO_4_ · 5H_2_O (0.007 g/l); yeast extract (1 g/l). After 4–5 days of cultivation, the fungal mycelia from each of the five culture conditions were homogenized in liquid nitrogen, and total RNA was extracted following a standard phenol/chloroform method [33]. RNA from all the culture conditions was pooled and sent to GATC Biotech AG for reverse transcription and sequencing via Illumina technology.

For proteomic analysis, liquid cultures (LC) with non-immobilized or immobilized mycelia on 2 × 2 × 1 cm polyurethane cubes (10 per vial) were run in 250 ml baffled flasks containing 100 ml medium according to Lomascolo *et al.* [17]. Three LC conditions were used: *(i)* 20 g/l maltose (LC-M), *(ii)* 5 g/l maltose, 15 g/l Avicel cellulose (Sigma) and 15 g/l autoclaved maize bran (ARD) (LC-M-MB-A), and *(iii)* 5 g/l maltose and 15 g/l micronized birchwood (LC-B). Solid-state fermentation (SSF) cultures were also performed with five different substrates: sugarcane bagasse (Orizaba, Mexico), banana skins, wood shavings (Farmer Litter, Weldom, France), hemp (Zolux Litter, Weldom, France) and micronized birchwood. Each substrate was homogenized in water to obtain a moisture content of 70% (w/w). Five grams of substrate (wet weight) was placed in a 250 ml flask and inoculated with 1.2 ml of mycelial suspension (50 ml of nutrient medium and two mycelial mats from precultures) according to a protocol adapted from Meza *et al.* [34, 35]. For each growth condition, culture supernatants were harvested after 3, 7 and 10 days of cultivation and then pooled.

For growth profiling on 35 carbon sources, *P. cinnabarinus* BRFM137 was grown on agar plates according to Espagne *et al.* [36], using either 10 g/l simple carbohydrates or 30 g/l complex carbohydrates. The kraft-lignin was purchased from Sigma (reference: 370959).

### Genome sequencing and data assembly

The *P. cinnabarinus* BRFM137 genome was sequenced by a combination of methods: (*i*) sequencing of genomic DNA and two normalized cDNA libraries obtained from cultures grown on different substrates (maltose, oat spelt xylan, cellulose and autoclaved maize bran) using 454/GS Roche FLX Titanium technology, (*ii*) sequencing of genomic DNA with Illumina/Solexa Genome Analyzer II technology, and (*iii*) sequencing of a 3 kbp paired-end genomic library using Illumina/Solexa Genome Analyzer II technology. The genomic Roche 454 read sets were uploaded to the ng6 storage environment [37]. Reads were cleaned using pyrocleaner [38], which applied a low-complexity filter followed by a read-size filter (over 100 bp) and a duplication-removal filter. The 454 reads were then assembled using wgs-assembler version 6.0. The Illumina mate pair reads were filtered out using the contig alignment information. All short aligned read pairs and long reads were then reassembled to produce contigs and scaffolds using the same assembly software versions. The 454 transcriptome reads of *P. cinnabarinus* were also cleaned using pyrocleaner, but this time the duplicated sequences were not filtered out. The reads were *de novo* assembled using tgicl (TIGR Gene Indices clustering tools) and annotated using various databases. The reads and contigs were aligned on the genome using Exonerate to produce gff files. These gff files were uploaded to gbrowse (http://genome-browser.toulouse.inra.fr:9090/cgi-bin/gb2/gbrowse/). Gene prediction was performed using Augustus [39] with the fungal gene model: *Phanerochaete chrysosporium*. The corresponding gff outputs were also uploaded to the gbrowse environment. Ensembl fungal transcripts of *Aspergillus fumigatus*, *Aspergillus terreus*, *Aspergillus nidulans*, *Schizosaccharomyces pombe*, *Aspergillus clavatus*, *Aspergillus niger*, *Aspergillus flavus*, *Aspergillus oryzae* were also aligned on the genome and the results uploaded to gbrowse. For *P. cinnabarinus,* a biomart environment was set up to link *de novo* contigs to their genomic alignment location (http://genomebrowser.toulouse.inra.fr:9090/biomart/martview).

All the data are available at the European Nucleotide Archive (ENA), EMBL-EBI, Accession number: [EMBL: PRJEB5237].

### Gene prediction and functional annotation

#### Orthologous groups construction

Orthologous groups (OGs) were built by running OrthoMCL [40] software on the best protein models from: 1) *P. cinnabarinus BRFM137*, 2) *Trametes versicolor*, (TaxID: 717944) 3) *Postia placenta* (TaxID: 561896), 4) *Phanerochaete chrysosporium RP-78* (TaxID: 273507), 5) *Schizophyllum commune H4-8* (TaxID: 578458), 6) *Coprinopsis cinerea okayama7#130* (TaxID: 240176), 7) *Laccaria bicolor S238N-H82* (TaxID: 486041), 8) *Agaricus bisporus* (TaxID: 936046), 9) *Gloeophyllum trabeum* (TaxID: 670483), 10) *Ustilago maydis* (TaxID: 5270), 11) *Saccharomyces cerevisiae RM11-1a* (TaxID: 285006), 12) *Schizosaccharomyces pombe* (TaxID: 4896), 13) *Aspergillus niger* (TaxID: 380704), 225 14) *Trichoderma reesei QM6a* (TaxId: 431241), 15) *Nectria haematococca* (TaxID: 140110), 16) *Neurospora crassa* (TaxID: 367110), 17) *Myceliophthora thermophila* (TaxID: 573729), 18) *Chaetomium globosum* (TaxID: 306901), 19) *Mucor circinelloides* (TaxID: 747725), 20) *Homo sapiens* (TaxID: 9606) and 21) *Arabidopsis thaliana* (TaxID: 3702). Each OG is a set of proteins across one or more species in the 21 listed genomes that represents putative orthologs and in-paralogs. All-versus-all BLASTP was set a 10^−8^ cutoff.

#### Global functional annotation

Global functional annotation was based on the analysis of each OG. All 15788 OGs were used as a seed for the functional annotation process based on the bioinformatics initiative Gene Ontology [41]. OGs containing at least one sequence from *P. cinnabarinus* were selected (7002 OGs). All sequences included in OG were ordered following the species list above. Sequences from each OG were queried using BLAST against the NCBI non-redundant (NR) protein database. A strict *E*-value threshold of 10^−120^ was applied to select homologous sequences retrieved by BlastP. These homologs were mapped to the global Gene Ontology annotation files (ftp://ftp.pir.georgetown.edu/databases/idmapping/idmapping.tb.gz).

If GO information was retrieved for the first sequence, the process was ended; if no information was retrieved for the first sequence in the OG list, the second sequence was used for mapping. In the particular case where several sequences were present in the same species, sequences were ordered by length. All the coding sequences (CDS) not included in OGs were directly BLASTed as described above.

#### Identification of repeated sequences

RepeatScout [42] was used to identify *de novo* DNA repeats in the *P. cinnarinus* genome. Default parameters (with *l* = 15) were used. The RepeatScout library was then filtered as follows: *i*) all the sequences less than 100 bp in size were discarded; *ii*) repeats counting less than ten copies in the genome were removed (as they may correspond to protein-coding gene families) and *iii*) repeats having significant hits to known proteins in UNIPROT (The UNIPROT Consortium, 2008) other than proteins known to belong to transposable elements (TEs) were removed. The remaining consensus sequences were annotated manually by a TBLASTX search [43] against RepBase [44] to classify them into known TE families. To identify full-length long terminal repeat (LTR) retrotransposons, a second *de novo* search was performed with LTR_STRUC [45]. The TBLASTX algorithm was to check the full-length candidate LTR retrotransposon sequences for homology against the sequences from the RepBase database. The number of repeat element occurrences and the percent of genome coverage were assessed using RepeatMasker [46] by masking the genome assembly with the consensus sequences coming from the RepeatScout and LTR_STRUC pipelines. MISA (http://pgrc.ipk-gatersleben.de/misa/download/misa.pl) was used with default parameters to identify mono- to hexanucleotide simple sequence repeat (SSR) motifs. Mini-satellites (motif of 7 to 100 bp) and satellites (motif >100 bp) were searched for in the *P. cinnabarinus* genome using Tandem Repeats Finder software [47] with the following parameters: 2; 7; 7; 80; 10; 50; 500.

#### Carbohydrate-active enzyme and lignin degradation enzyme annotation

All putative proteins were compared to the entries in the CAZy database [48, 49] using BLASTP. The proteins with *E*-values smaller than 0.1 were further screened by a combination of BLAST searches against individual protein modules belonging to the AA (Auxiliary Activities), GH (Glycosyl Hydrolases), GT (GlycosylTransferases), PL (Polysaccharide Lyases), CE (Carbohydrate Esterases) and CBM (Carbohydrate-Binding Modules) classes (http://www.cazy.org/). HMMer 3 [50] was used to query against a collection of custom-made hidden Markov model (HMM) profiles constructed for each CAZy family. All identified proteins were then manually curated. Within families, subfamilies were manually defined according to their homology relationships between members of the focal family. Protein sequences obtained from automatic prediction by Augustus software were annotated via this procedure, and all identified proteins were then manually curated.

Structural annotation of the corresponding oxidative encoding genes (number, size and position of introns) was checked manually. To do this, each AA sequence detected was BLASTP-searched against the NCBI non-redundant database. The results with the most satisfactory *E*-values and coverage were retained.

Then, first the target protein sequence was aligned with the sequence previously selected by BLASTP using ClustalW (http://www.genome.jp/tools/clustalw/); second, the target nucleic acid sequence was translated in three reading frames (http://www.ebi.ac.uk/Tools/st/emboss_sixpack/). Gene intron splice sites were determined based on consensus sequences fitting the GT-AG rule as described in Breathnach *et al.* [51].

### Identification of proteins in secretomes by LC-MS/MS analysis

Proteins from the diafiltered supernatants of *P. cinnabarinus* BRFM137 cultures were separated by 1D SDS-PAGE electrophoresis according to the protocol of Couturier *et al.* [52]. After protein trypsinolysis, peptide analysis was performed by LC-MS/MS as described in Arfi *et al.* [53] using the PAPPSO platform facilities (Jouy-en-Josas, France; http://pappso.inra.fr). Based on the list of peptides, proteins were identified by querying the MS/MS data against the predicted proteins obtained from the *P. cinnabarinus* genome *de novo* sequencing data.

#### Annotation of protein secretion and glycosylation pathways

*A. niger* proteins related to protein secretion and glycosylation according to Pel *et al.* [54] and extended with additional proteins were used in a BLASTP search towards the *P. cinnabarinus* fasta file. The first hits were compared to the *A. niger* proteins to identify bi-directional BLAST best hits. An *E*-value cut-off of 10^−10^ was used. The description of the gene products was taken from the Saccharomyces Genome Database (SGD) after identifying the *S. cerevisiae* orthologs.

## Results and discussion

### Characteristics of the *P. cinnabarinus*genome

The genome of the monokaryotic strain *P. cinnabarinus* BRFM137 was sequenced by 454 pyrosequencing and Illumina sequencing runs to reach a final 31-fold coverage. The genome was ultimately assembled into 784 scaffolds with N50 of 165118 bp. Table 1 reports the features of the assembled genome sequences. The G + C content of the *P. cinnabarinus* genome was 52.55%. Genome size was 33.67 Mb and a total of 10,442 ORFs were identified in the structural annotation procedure. The number of ORFs in *P. cinnabarinus* is close to the average number among the order Polyporales. For instance, *Phanerochaete chrysosporium*, *Postia placenta, Wolfiporia cocos* and *Ceriporiopsis subvermispora* count 10048, 12541, 12747 and 12125 detected ORFs in their genomes, respectively [4, 6, 55, 56]. *P. cinnabarinus* genome size is slightly lower than in *P. placenta* (42.5 Mb), *C. subvermispora* (39 Mb) and *W. cocos* (50.5 Mb).

**Table 1:** **Statistical assembly of the** ***P. cinnabarinus*** **genome**

Total scaffolds	784
Total bases in scaffolds	33 133 717
Total span of scaffolds	33 638 736
Coverage	31.0916
Length of genome assembly (Mb)	33.67
ORFs number	10442
GC content (%)	52.55
Average number of exons per gene	6.7
Average exon size (bp)	257.42
Average coding sequence size (bp)	1774.36
N50 scaffold bases	165118

**Figure 1:**
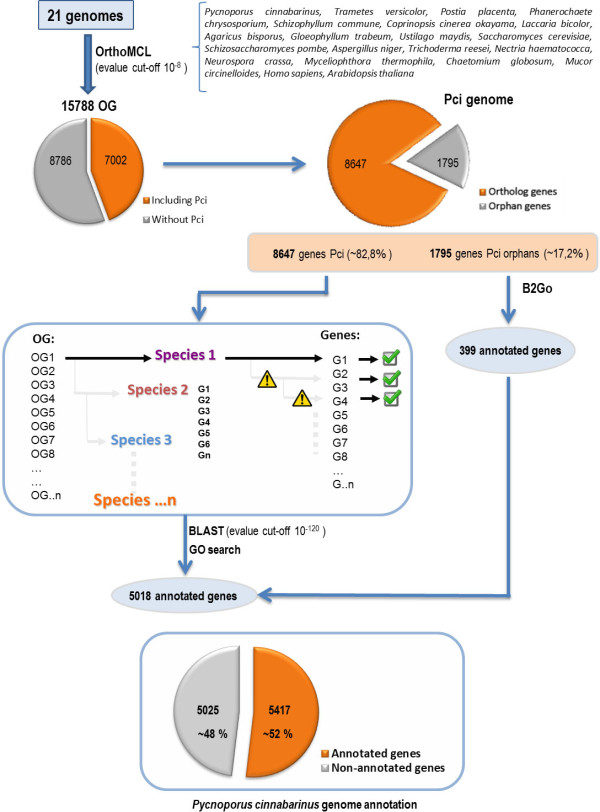
**Annotation strategy for** ***P. cinnabarinus*** **based on a phylogenomic approach.** Orthologous groups (OGs) were formed from 21 genomes by running the OrthoMCL software using a BLASTP cutoff *E*- value of 1e^−8^. OGs containing at least one sequence from *P. cinnabarinus* were selected (7002 OGs) and used as a seed for the functional annotation process based on the bioinformatics initiative Gene Ontology. Sequences from each OG were BLAST-queried against a NCBI non-redundant (NR) protein database using a cutoff *E*-value of 10^−120^. The mapping procedure was carried out with the global Gene Ontology annotation files. The process was ended once GO information was retrieved. For orphan genes, the coding sequences were directly annotated using B2Go procedures.

**Figure 2:**
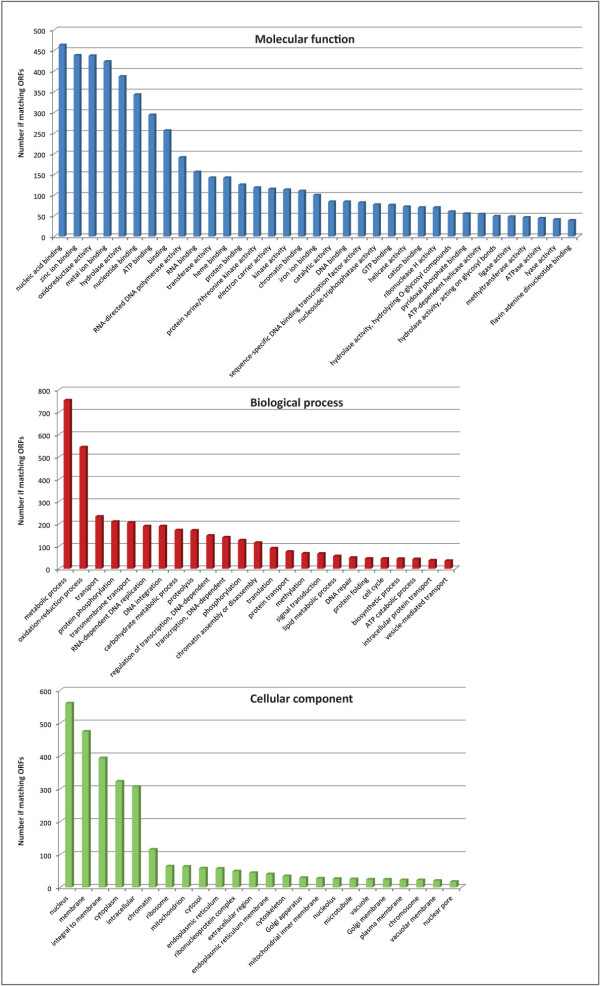
**Annotation of the** ***P. cinnabarinus*** **genome.** Classification scheme is summarized in three main GO categories, i.e. biological process, cellular component, molecular function. Some genes have more than one GO annotation.

**Figure 3:**
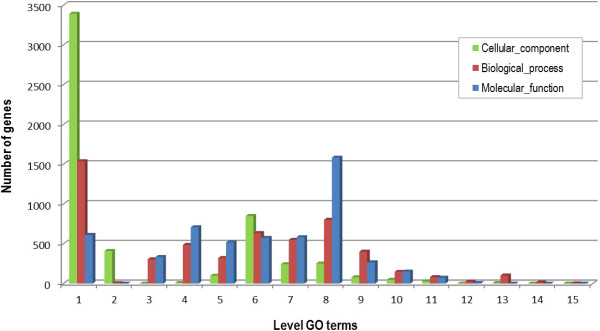
**GO annotation depth.** A bottom-up, recursive depth search is carried out to determine the level of GO terms.

In general, functional annotation hinges on the propagation of existing functional information via single homology searches. The resolution of functional inference could be improved by differentiating homologs into orthologs (homologous genes resulting from a speciation event) and paralogs (homologous genes resulting from a duplication event) [57]. Orthologs are assumed to have more chance of sharing the same function than paralogs. Gene duplication is an essential contributing factor for evolving novel functions, and one of the duplicates could undergo evolutionary events such as sub-functionalization, neofunctionalization, etc. (see [58, 59] for review). We therefore based our annotation strategy on the searches for OGs within 21 selected genomes followed by similarity searches from each OG. An outline flow of the functional annotation procedure based on this phylogenomic approach is shown in Figure 1. 15,788 OGs were retrieved using a best reciprocal hit approach. The OGs included 8,647 putative CDS from *P. cinnabarinus*, totaling ~83% of total CDS. Based on a sequence homology searches within each OG against the NR database using a strict *E*-value cutoff of 10^−120^, 5,018 genes were annotated across the GO categories. In addition, 399 orphan genes were annotated using the standard Blast2GO procedure. The annotation procedure enabled us to annotate 5,417 CDS corresponding to ~52% of total CDS (Additional file 1: Table S1). To compare with the classical method, fewer than 30% of total CDS were annotated using the Blast2GO procedure. Our approach based on ortholog clustering enables us to infer functional information directly from OGs using a subsequent drastic threshold for similarity searches and offers a conceptual framework for inferring information from various genomes. The 5,417 annotated genes were grouped into functional groups (Figure 2). Finally, a GO tree depth was calculated to assess amount and quality of GO annotations (Figure 3).

Repeated sequences were identified in the genome of *P. cinnabarinus* and a library of 1,118 consensus sequences was generated using RepeatScout [41]. After the different filtering steps, we were left with 190 consensus sequences: 13, 9, 5 and 5 consensus sequences showed homologies with Class 1 gypsy, copia, DIRS and Long Interspersed Element (LINE) retrotransposons, respectively, and 8 with Class 2 transposons (Table 2). The remaining 150 consensus sequences were uncategorized. Of the 9 putative full-length LTRs identified using LTR_STRUC, three were attributed to Gypsy/Ty3-like elements and two to Copia/Ty1-like elements. The remaining four sequences are excluded from further analyses. RepeatMasker masked 8.21% of the genome assembly: 2.91% by repeated elements belonging to unknown/uncategorized families, 2.5% by Class 1 *Gypsy* retrotransposons, 0.95 by Class 1 *Copia* retrotransposons, 0.2 by Class 1 DIRS retrotransposons, 0.7 by Class 1 LINE retrotransposons and 0.95 by Class 2 DNA transposons (Table 2).

**Table 2:** **Number of repeated sequences in the** ***P. cinnabarinus*** **genome**

	Number of families	Number of copies	Genome assembly coverage
**Class-1 LTR Gypsy-like**	16 (13* + 3**)	642	2.5
**Class-1 LTR Copia-like**	11 (9* + 2**)	306	0.95
**Class-1 DIRS**	5	62	0.2
**Class-1 LINE**	5	163	0.7
**Class-2 DNA Transposons**	8	251	0.95
**Uncategorized**	150	2831	2.91
**All**	168	4255	8.21

*Number of elements identified by RepeatScout pipeline.

**Number of elements identified by LTR_STRUC pipeline.

The number of full-length LTR was lower in *P. cinnabarinus* than in other white-rots [6], although the TE genome coverage was in the range of other white-rot fungi. A total of 1,707 SSRs were identified in the *P. cinnabarinus* genome corresponding to 350 mono-, 380 di-, 820 tri-, 91 tetra-, 25 penta- and 41 hexanucleotide motifs. A total of 2368 mini-satellites and 10 satellites were identified for a genome coverage of 0.42% and 0.01%, respectively. The number of microsatellites was in the range of those found in other white-rot and Polyporaceae genomes, although the genome of *P. cinnabarinus* was less rich in mini-satellite and satellite sequences [60].

### Carbohydrate metabolism, lignin-degrading oxidoreductases and wood decay

Carbohydrates and lignin are intimately interconnected in all land-plant cell-walls. The accessibility of all cell-wall components *i.e.* cellulose, hemicellulose, pectin and lignin, is strongly limited by the covalent cross-linkages of the constituents which create an intricate network and a physical barrier that resists microbial breakdown. Among the predicted lignin-degrading activities, a total of five laccases (AA1_1), one ferroxidase (AA1_2), one multicopper oxidase (AA1), nine ligninolytic peroxidases (AA2) including lignin peroxidases (LiP), manganese peroxidases (MnP) and versatile peroxidases (VP), one cellobiose dehydrogenase containing an iron reductase domain (AA8-AA3_1), three aryl-alcohol oxidases and one glucose oxidase (AA3_2), two alcohol oxidases (AA3_3), two pyranose oxidases (AA3_4), seven copper radical oxidases (AA5_1), one benzoquinone reductase (AA6), and one iron reductase domain (AA8) linked to a CBM1 were identified (Table 3 and Additional file 2: Table S2). *P. cinnabarinus* was initially considered to lack class-II peroxidases based on extracellular activities in the culture medium [16]. Remarkably, nine class II peroxidases were annotated and divided into at least four LiP, three MnP, one VP and one atypical VP. On average, white-rot fungi have 12 members of the AA2 family (Table 4). The only exception is *S. commune* in which the AA2 family is absent [61], although it is considered as a white-rot fungus despite limited lignin-degrading ability. Members of family AA2 can be considered as one of the most important family markers to differentiate white-rot and brown-rot fungi, since brown-rot (BR) fungi contain no AA2 members [6, 49]. In addition to class II peroxidases, *P. cinnabarinus* contains several laccases (AA1_1) and one cellobiose dehydrogenase (AA8-AA3_1), meaning that this fungus contains a complete, versatile ligninolytic enzymatic spectrum. A number of enzymes are proposed to supply the hydrogen peroxide required for oxidase activity. Among these, the best established candidate is glyoxal oxidase of family AA5_1, and *P. cinnabarinus* has seven candidate gene models in this family. Interestingly, *P. cinnabarinus* also possesses several other hydrogen peroxide providers, such as GMC oxidoreductases from family AA3_2 which includes at least three aryl-alcohol oxidases. In summary, the white-rot fungus *P. cinnabarinus* possesses a complete enzymatic arsenal for lignin breakdown. The full set of ligninolytic enzymes identified suggests that this fungus may exploit different strategies for ligninolysis, including oxidation mediated by class II peroxidases requiring hydrogen peroxide or by laccases in the presence of redox mediators, or via Fenton chemistry [49, 62, 63].

**Table 3:** **Global composition of AA encoding genes found in** ***P. cinnabarinus*** **BRFM137**

Family ^a^	Known activities	Total number
AA1_1	Laccase	5
AA1_2	Ferroxidase	1
AA1	Multicopper oxidase	1
AA2	Class II peroxidase	9 (+1 partial)
AA3_1	Cellobiose dehydrogenase	1
AA3_2^b^	Aryl-alcohol oxidase/	19
	Glucose oxidase	
AA3_3	Alcohol oxidase	2
AA3_4	Pyranose oxidase	2
AA5_1	Glyoxal oxidase	7
AA6	1,4-benzoquinone reductase	1
AA8	Iron reductase domain	2
AA9	Lytic polysaccharide monooxygenase	15

^a^Known (sub)family activities are as follows: AA1_1: laccase, AA1_2: ferroxidase, AA1: multicopper oxidase, AA2: class II peroxidase; AA3_1: cellobiose dehydrogenase; AA3_2: aryl alcohol oxidase, glucose oxidase; AA3_3: alcohol oxidase; AA3_4: pyranose oxidase; AA5_1: glyoxal oxidase, copper radical oxidase; AA6: benzoquinone reductase; AA8: iron reductase domain; AA9: LPMO. ^b^ Including 3 AO and 1 GOx. According to [49].

**Table 4:** **Comparison of the CAZy repertoire identified in the selected white-rot and brown-rot fungal genomes**

CAZy families ^a^	Aude	Cesu	Disq	Fome	Galu	Gano	Hean	Phch	Pust	Pyci	Scco	Sthi	Trve	Copu	Dac	Fopi	Gltr	Popl	Sela	Woco
GH1	1	3	4	5	3	3	2	2	1	1	3	3	2	3	1	2	5	2	3	1
GH2	7	4	4	2	3	3	3	2	4	3	4	3	5	5	3	4	4	3	3	3
GH3	14	6	8	8	12	13	12	11	14	7	12	17	13	13	9	12	11	6	11	8
GH5	43	18	19	20	19	18	16	19	18	17	16	20	22	21	24	19	19	17	20	18
GH6	2	1	1	2	1	1	1	1	1	1	1	1	1	2	0	0	0	0	1	0
GH7	8	3	4	2	3	3	1	9	5	3	2	3	4	2	0	0	0	0	0	0
GH10	4	6	5	4	7	10	2	6	5	2	5	6	6	3	3	2	3	3	1	4
GH11	3	1	0	0	0	0	0	1	1	0	1	1	0	0	0	0	0	0	0	0
GH12	1	2	3	3	3	3	4	2	2	3	1	5	5	4	1	2	2	2	1	2
GH13	10	7	10	6	9	8	8	9	10	7	13	14	7	6	11	7	9	7	7	11
GH15	2	3	2	1	3	3	5	2	4	1	3	3	4	2	2	4	2	2	2	2
GH26	0	0	0	0	0	0	0	0	0	0	1	0	0	0	4	0	0	0	0	0
GH27	5	4	6	4	6	3	4	3	5	1	1	5	4	4	2	4	3	3	3	3
GH28	14	6	7	17	13	10	8	4	13	4	3	17	11	13	6	12	10	8	7	9
GH29	3	0	0	0	0	0	2	0	1	0	2	4	0	4	2	0	1	0	1	0
GH31	11	5	6	5	6	7	10	6	8	5	4	8	5	12	6	5	5	4	5	5
GH32	2	0	2	0	1	1	1	0	1	1	1	1	3	2	1	3	1	0	0	0
GH35	6	1	3	2	10	7	4	3	4	2	4	7	2	2	1	2	2	1	3	2
GH36	0	0	0	0	0	0	0	0	0	0	0	0	0	0	1	0	0	0	0	0
GH43	28	2	7	7	11	12	4	4	7	2	19	12	3	6	5	7	6	1	2	1
GH45	2	2	1	0	2	2	2	2	1	1	1	1	2	1	1	1	1	0	0	0
GH51	3	2	2	1	2	2	1	2	3	0	2	3	2	3	2	4	4	1	1	4
GH53	1	6	1	1	1	1	1	1	2	0	1	2	1	1	1	1	2	1	1	1
GH54	0	0	0	0	0	0	0	0	2	0	0	0	0	0	0	0	0	0	0	0
GH62	0	0	0	0	0	0	0	0	0	0	1	0	0	0	0	0	0	0	0	0
GH74	1	1	1	4	1	1	1	4	2	1	1	2	1	0	0	0	1	0	1	0
GH78	4	1	5	2	5	4	2	1	7	1	3	3	3	2	0	3	2	3	2	3
GH88	2	1	1	2	1	1	1	1	1	2	1	1	1	1	1	1	1	1	1	1
GH93	1	0	1	0	2	2	0	0	1	0	2	1	0	1	0	0	0	0	0	0
GH95	1	1	1	2	1	2	1	1	1	1	2	1	1	1	0	1	1	1	1	1
GH105	3	0	1	1	1	1	2	0	2	0	2	2	1	0	0	2	2	0	0	0
GH115	2	2	2	3	4	3	1	1	1	2	2	2	2	2	2	1	2	1	1	2
GH131	2	1	3	2	3	3	2	3	2	3	2	3	3	2	1	1	1	0	2	0
**Total GH**	**186**	**89**	**110**	**106**	**133**	**127**	**101**	**100**	**129**	**71**	**116**	**151**	**114**	**118**	**90**	**100**	**100**	**67**	**80**	**81**
PL1	2	0	0	2	0	0	2	0	4	0	5	4	0	0	0	0	0	0	0	0
PL3	1	0	0	0	0	0	0	0	0	0	4	0	0	0	0	0	0	0	0	0
PL4	1	0	1	0	0	0	1	0	3	1	3	3	1	0	0	0	2	0	0	0
PL9	0	0	0	0	0	0	0	0	0	0	1	0	0	0	0	0	0	0	0	0
**Total PL**	**4**	**0**	**1**	**2**	**0**	**0**	**3**	**0**	**7**	**1**	**13**	**7**	**1**	**0**	**0**	**0**	**2**	**0**	**0**	**0**
CE1	4	2	0	0	2	2	1	4	2	3	11	1	3	0	0	0	1	0	0	0
CE8	2	2	3	3	3	3	3	2	6	1	2	5	2	2	3	2	2	2	2	1
CE12	2	0	2	2	1	1	2	0	1	0	2	3	0	0	0	0	0	0	0	0
**Total CE**	**8**	**4**	**5**	**5**	**6**	**6**	**6**	**6**	**9**	**4**	**15**	**9**	**5**	**2**	**3**	**2**	**3**	**2**	**2**	**1**
CBM1	43	17	17	6	14	18	17	30	21	17	5	17	23	2	1	0	1	0	8	0
CBM5	8	3	5	5	10	9	5	3	4	3	3	10	6	11	1	4	4	5	5	4
CBM12	2	1	1	0	1	1	1	0	0	1	1	1	1	1	0	1	1	1	1	1
CBM13	4	6	11	4	9	8	1	5	7	4	17	4	6	6	2	11	1	15	5	5
CBM18	1	1	1	0	2	1	1	1	1	1	1	1	1	1	1	1	0	1	1	1
CBM20	4	4	2	2	3	3	3	2	4	1	1	5	4	2	1	2	2	1	2	1
CBM21	3	2	2	2	2	2	2	2	3	2	3	1	2	2	3	2	2	2	1	1
CBM35	2	0	1	1	0	1	2	1	1	0	1	2	1	0	1	1	2	1	2	1
CBM38	0	0	0	0	0	0	0	0	0	0	0	0	0	1	0	0	0	0	0	0
CBM42	4	0	0	0	0	0	0	0	2	0	0	0	0	0	0	0	0	0	0	0
CBM43	1	1	1	1	1	1	1	1	1	1	1	2	1	1	1	1	1	2	1	1
CBM48	3	2	3	3	3	3	2	1	3	2	4	3	3	3	2	3	3	3	2	3
CBM50	21	1	2	2	8	10	1	1	10	1	5	1	1	1	1	15	2	4	1	0
CBM63	0	0	0	0	0	0	0	0	0	0	1	0	0	0	0	0	0	0	0	0
**Total CBM**	**96**	**38**	**46**	**26**	**53**	**57**	**36**	**47**	**57**	**33**	**43**	**47**	**49**	**31**	**14**	**41**	**19**	**35**	**29**	**18**
AA1_1	0	7	11	10	13	16	14	0	12	5	2	15	7	6	0	5	4	2	4	3
AA1_2	1	1	1	1	1	1	1	1	1	1	0	2	2	1	2	1	1	1	1	1
AA1_3	5	0	0	0	0	0	0	0	0	0	0	0	0	0	0	0	0	0	0	0
AA2	18	16	12	17	8	9	7	16	11	9	0	6	26	0	0	0	0	0	0	0
AA3_1	1	1	1	1	1	1	1	1	1	1	1	1	1	1	0	0	1	0	2	0
AA3_2^b^	3	6	11	2	4	8	13	3	6	4	2	15	5	0	0	1	6	3	0	0
AA3_3	6	3	4	3	3	5	3	3	4	2	4	7	4	5	1	4	2	4	5	5
AA3_4	3	0	0	0	0	0	0	1	1	2	0	0	1	0	0	0	1	0	0	0
AA4	0	0	0	0	0	0	0	0	0	0	0	0	0	0	2	0	3	0	0	0
AA5_1	8	3	9	4	9	9	5	7	9	7	2	8	9	6	3	4	2	2	3	4
AA5_2	0	0	0	0	0	0	0	0	0	0	0	0	0	0	0	0	0	0	0	0
AA6	4	0	1	3	1	2	2	4	2	1	4	1	1	2	1	1	3	1	2	1
AA7	2	0	4	0	4	4	3	0	1	0	4	3	0	0	3	5	0	0	0	0
AA8	2	2	2	1	2	2	2	2	1	2	3	2	2	4	0	0	0	0	4	0
AA9	20	9	15	13	15	16	10	15	14	15	22	16	18	10	0	4	4	2	5	2
AA11	0	0	0	0	0	0	0	0	0	0	2	0	0	0	0	0	0	0	0	0
**Total AA**	**73**	**48**	**71**	**55**	**61**	**73**	**61**	**53**	**63**	**49**	**46**	**76**	**76**	**35**	**12**	**25**	**27**	**15**	**26**	**16**

^a^Selection of the CAZy families involved in plant cell wall degradation. Full list of CAZy families are provided in Additional file 1: Table S1.

^b^AA3_2 includes only models with similarity to aryl-alcohol oxidase and glucose oxidase

**Species list** *: Auricularia delicata (Aude); Ceriporiopsis subvermispora (Cesu); Dichomitus squalens (Disq); Fomitiporia mediterranea (Fome); Ganoderma lucidum (Galu); Ganoderma sp. (Gano); Heterobasidion annosum (Hean); Phanerochaete chrysosporium (Phch); Punctularia strigosozonata (Pust); Pycnoporus cinnabarinus (Pyci); Schizophyllum commune (Scco); Stereum hirsutum (Sthi); Trametes versicolor (Trve); Coniophora puteana (Copu); Dacryopinax sp. (Dac); Fomitopsis pinicola (Fopi); Gloeophyllum trabeum (Gltr); Postia placenta (Popl); Serpula lacrymans (Sela); Wolfiporia cocos (Woco).*

**Figure 4:**
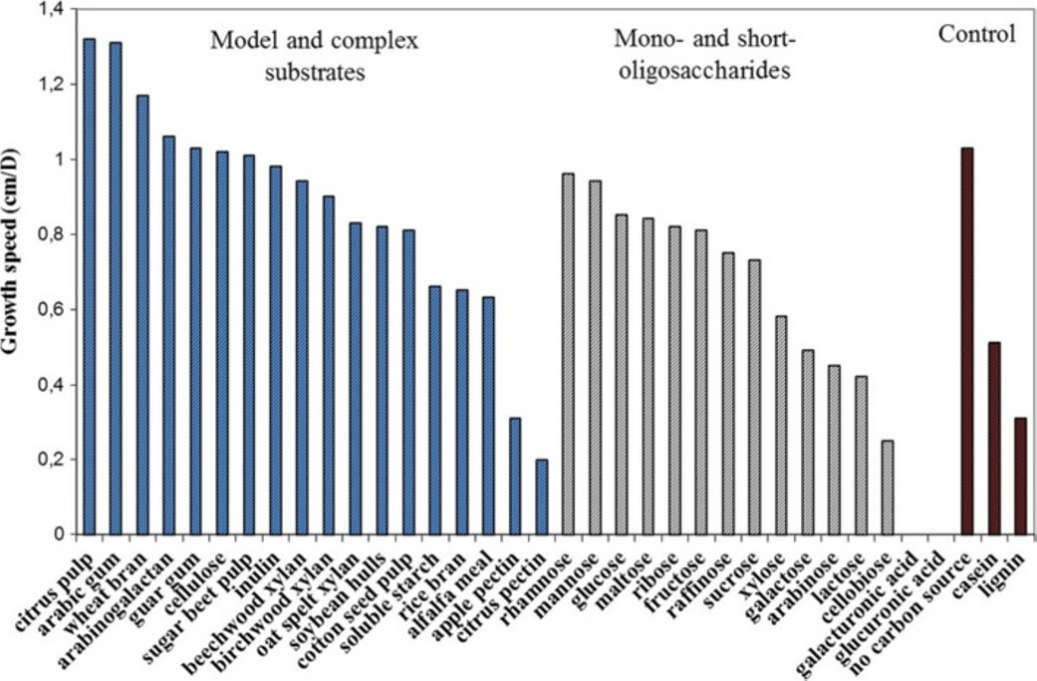
**Growth speed of** ***P. cinnabarinus*** **on different substrates.**

*P. cinnabarinus* is fully equipped with putative enzymes from families classically involved in cellulose degradation (GH1, GH3, GH5, GH6, GH7, GH12, GH45) and can grow on pure cellulose. However, *P. cinnabarinus* possesses the smallest number of GH members among the white-rot fungi. The *P. cinnabarinus* genome encodes 15 lytic polysaccharide monooxygenases (LPMOs) of family AA9, a number similar to that encoded by other white-rot fungal genomes (Table 4). The *P. cinnabarinus* BRFM137 genome contains a gene encoding a CDH (ORF scf185013.g1). This gene codes for the CDH already described by Moukha *et al.* [11], Sigoillot *et al.* [64] and Bey *et al.* [65]. Concerning xylan degradation, only two GH10 and two GH43 enzymes were identified in *P. cinnabarinus*, which is less than the average number of representatives in the white-rot group (respectively of 5.2 and 9). No members of family GH51 could be found in the *P. cinnabarinus* genome. The GH51 family includes α-L-arabinofuranosidases acting on terminal non-reducing α-L-arabinofuranose residues in arabinose-containing compounds [66]. Terminal arabinose residues are found in the rhamnogalacturonan I from dicot primary cell walls, and glucuronoarabinoxylan from grass primary cell walls, so the absence of GH51 could partly constrain the complete degradation of hemicelluloses and pectic polysaccharides in *P. cinnabarinus* and is consistent with the lack of such cell wall components in wood. The number of other *P. cinnabarinus* genes encoding pectinolytic enzymes also seems to be limited. The members of family GH28 are fewer than the average number found in other fungi, and no representative of family GH54 including α-L-arabinofuranosidase was found. Also, *P. cinnabarinus* contains no candidate gene of the pectinolytic families PL1 (pectin/pectate lyase), PL3 (pectate lyase), PL9 (pectate lyase), CE12 (rhamnogalacturonan acetyl esterase) or GH53 (endo-β-1,4-galactanase). *P. cinnabarinus* is the only fungus lacking a family GH53 member among the selected white- and brown-rots. Family GH53 enzymes degrade galactans and arabinogalactans in the pectic component of plant cell walls. This genomic repertoire is consistent with the very poor growth of *P. cinnabarinus* observed on apple pectin and citrus pectin as substrates (Figure 4).

In conclusion, white-rot fungi possess more representatives of lignocellulolytic enzymes than the brown-rot group, especially in the families AA2 (12 *vs*. 0) AA3_2 (6.3 *vs*. 1.4), AA5_1 (6.8 *vs*. 3.4), AA9 (15.2 *vs*. 3.8) and CBM1 (18.8 *vs*. 1.7) (Table 4). Based on these results, *P. cinnabarinus* clearly belongs to the classical white-rot fungi, with a distribution typical of all the specific families found for this nutritional strategy.

**Figure 5:**
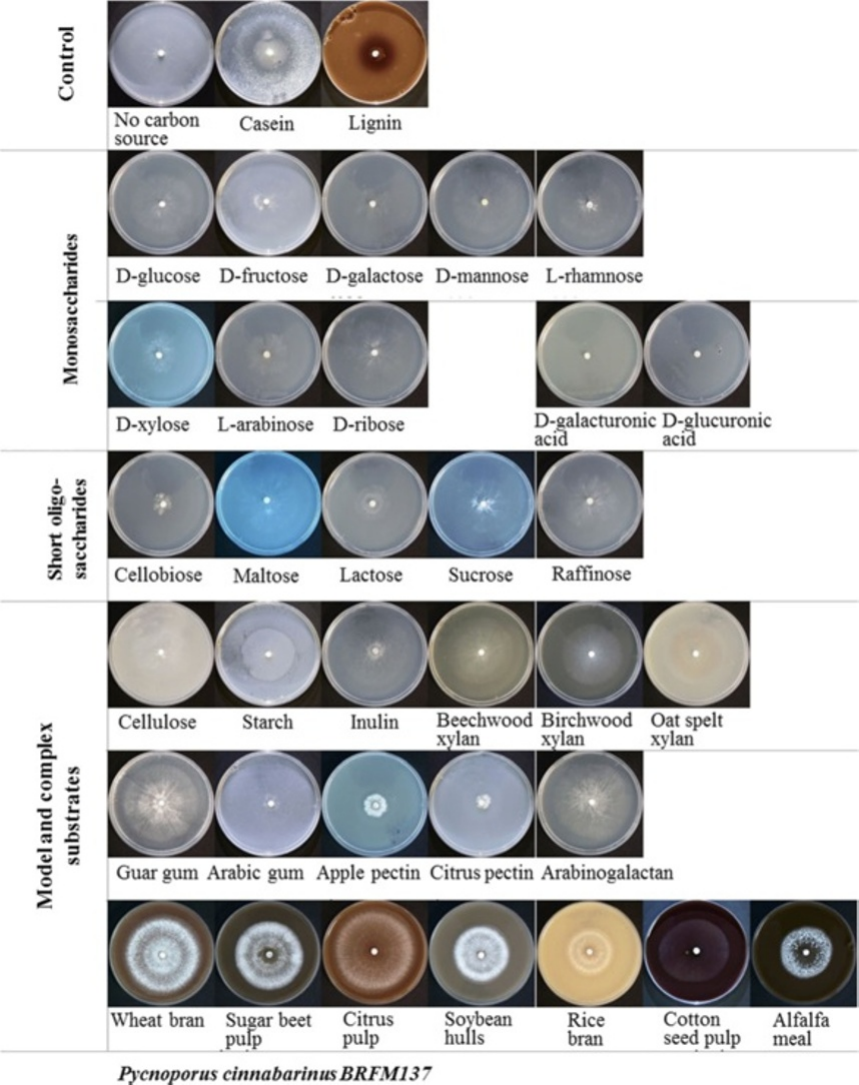
**Growth profiling of** ***P. cinnabarinus*** **on different substrates.**

To study the growth ability of *P. cinnabarinus*, growth profiling was performed on 35 carbon sources, including mono-, oligo- or polysaccharides, crude plant biomass, casein and lignin, and the profiles were compared with the CAZy gene content of the *P. cinnabarinus* genome. On average, growth was better on crude plant biomass substrates than on pure mono- oligo- or polysaccharides (Figures 4 and 5). Interestingly, growth on cotton seed hulls was poor, not only compared with the other plant biomass substrates but also compared with several pure substrates. Previous studies suggest that this is probably due to the high lignin content of cotton seed hulls (about 20–25%). The other plant biomass substrates are poorer in lignin (2–4%), suggesting that high lignin content inhibits growth of *P. cinnabarinus* in the culture conditions tested. This correlates with very poor growth on kraft lignin as sole substrate. As lignin may not be sole carbon source, the poor growth could be related to the possible impurities from lignocellulose introduced during lignin preparation. Growth is better on galactomannan (guar gum) than on xylan, suggesting a better mannan degrading system. Endomannanases are found in GH5 and GH26, but there are no family GH26 members in the *P. cinnabarinus* genome, suggesting that the good growth is mainly due to the GH5_7 endomannanase, together with the three GH2 β-mannosidases and one GH27 α-galactosidase. Inulin- and sucrose-degrading enzymes are found in GH32, but only one member of family GH32 is found in the *P. cinnabarinus* genome. Considering that growth on sucrose is significantly better than growth on inulin, this gene probably encodes an invertase rather than an inulinase. These growth profiling studies estimated growth speed by measuring the diameter of the on-plate fungal mycelium on plates. However, growth is also related to density of mycelium with dense, medium and thin mycelia on the plates. The fast growth on poor media, especially when no carbon source is added, could also be due to thin mycelial expansion to avoid starvation.

### Gene structure and localization of the ligninolytic repertoire in *P. cinnabarinus*

#### Descriptions of the P. cinnabarinus laccases (AA1_1)

Five laccases *stricto sensu* (AA1_1), one multicopper oxidase (Mco, AA1) and one ferroxidase (AA1_2) sequence were identified in the genome and in the cDNA library, even partially (Additional file 3: Table S3). Structural annotation of the genes (designated *lac1* to *lac5*) was performed, and the gene *lcc3-1* 15 (or *lac1*) coding for LacI protein was identified [17, 67]. In 2000, Otterbein *et al.* [68] demonstrated the presence of a second laccase isoenzyme, called Lac2, in the culture medium of *P. cinnabarinus* BRFM137.

Lac2 was purified and its *N*-terminal sequence was determined [68]. However, the corresponding gene has never been identified and cloned, and the biochemical properties of the Lac2 protein have never been determined. Based on the *N*-terminal sequence, we were able to determine the corresponding gene sequence, named *lac2,* from the strain BRFM137 genome sequencing data (Additional file 4: Table S4). The five laccase-encoding genes have a size of about 2.1–2.3 kb interrupted by 10 to 12 introns. Based on the intron and exon positions of each gene, we were able to classify the various laccase genes into three groups (Additional file 5: Figure S1). *Lac2/lac5* (12 introns) and *lac1/lac3* (10 introns) pairs have a similar structural organization with homologous intron positions, whereas the *lac4* gene is organized slightly differently (length of exons and introns). The *lac4* gene comprised 11 exons but showed a slightly different structure from *lac1* and *lac3*, and an experimentally-found stop codon was confirmed in exon 6 (Additional file 5: Figure S1). In contrast to other laccase-encoding genes, the full-length *lac4* mRNA could not be found. The multiplicity of laccase genes and their groupings are common features in fungi and are discussed in Additional file 6: Data S1 [69–80]. In the *P. cinnabarinus* BRFM137 genome, several laccase-encoding genes were identified on the same scaffold. For instance, the *lac1* and *lac3* genes were separated by approximately 23 kb in the same reading frame on scaffold 185007.

#### Descriptions of the P. cinnabarinus ligninolytic peroxidases (AA2)

**Figure 6:**
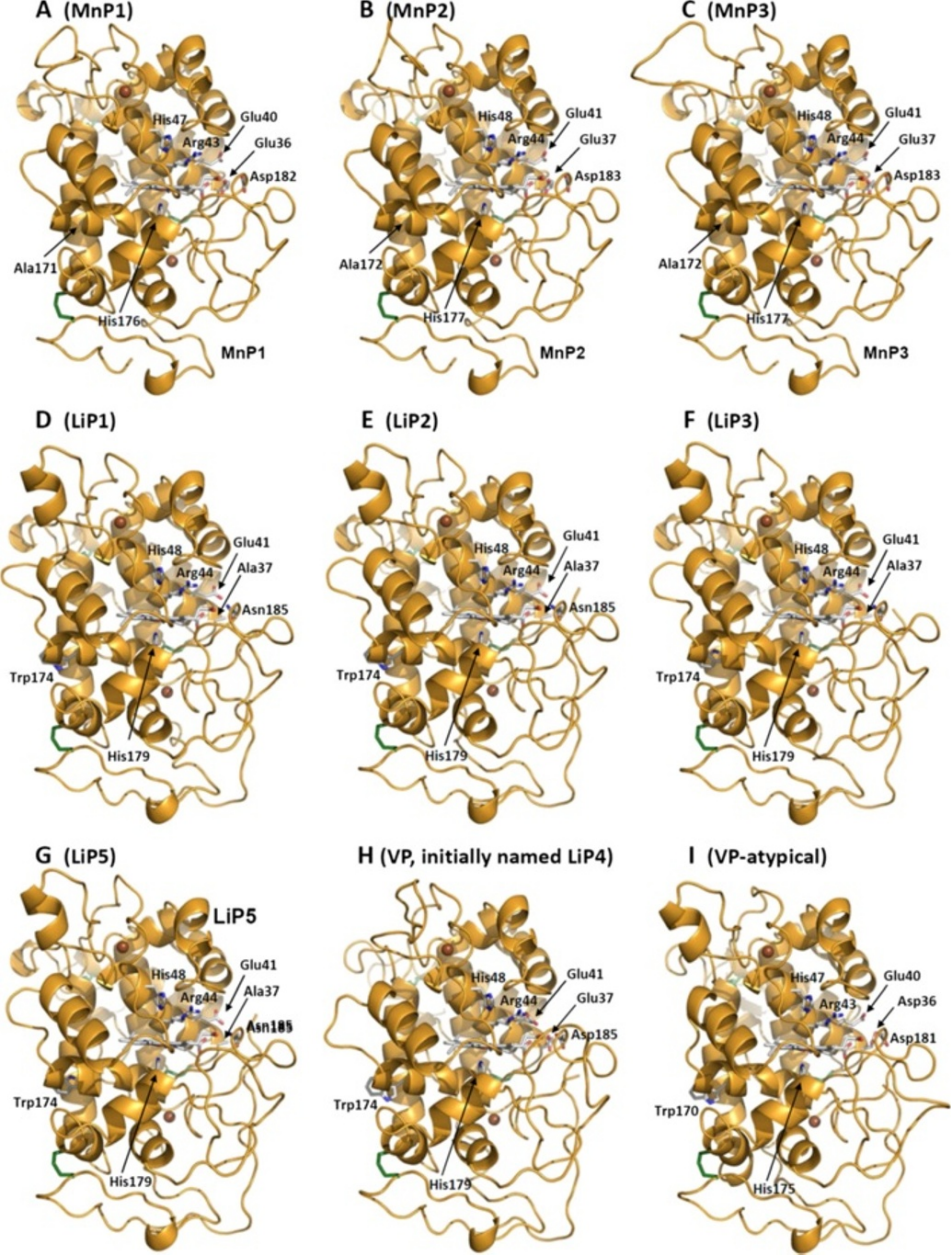
**Molecular models for the nine class-II heme peroxidases (AA2) found in the** ***P. cinnabarinus*** **genome.** MnP models **(A-C)** present a Mn^2+^ oxidation site characteristic of typical MnPs, formed by two glutamates and one aspartate at the internal heme propionate region; LiP models **(D-G)** exhibit a Trp residue exposed to the solvent, which has been involved in high-redox-potential aromatic compound oxidation by typical LiPs; the VP model **(H)** obtained for the only peroxidase of this family identified in the genome analysis evidences both the Mn^2+^ oxidation site and the Trp residue exposed to the solvent, characteristic of members of this class-II family; the atypical VP **(I)** contains an aspartate residue (Asp36) in a position occupied by a glutamate in VPs and MnPs. Two axial histidines, one acting as heme iron ligand (proximal histidine) and the second (distal histidine) contributing to the heme reaction with peroxide, together with an arginine residue characterizing class-II peroxidases are also shown in the nine molecular homology models. Four disulfide bridges are depicted as green sticks. These homology models were obtained at the Swiss-Model protein-homology server [83] using *P. eryngii* VPL (PDB entries 4FCS, 2VKA and 3FJW) and *P. chrysosporium* LiPH2 and LiPH8 (PDB entries 1LLP, 1B80 and 1B82) crystal structures as templates.

We have shown that the *P. cinnabarinus* genome encodes a large set of ligninolytic peroxidases of family AA2. Nine full-length AA2 sequences were detected from the genomic DNA of *P. cinnabarinus* BRFM137 (Table 3). After an initial automatic classification as LiPs and MnPs, they were manually reclassified following the strategy described by Ruiz-Dueñas *et al.* [81] for manual annotation of the complete inventory of heme peroxidases of *Pleurotus ostreatus*. This protocol was based on a combined analysis of the deduced amino acid sequences and structural homology models obtained using the crystal structures of related enzymes as templates. The identified members of family AA2 share common structural features, including four disulfide bridges and residues coordinating two calcium ions, a proximal histidine (acting as fifth heme iron ligand), and distal histidine and arginine residues (involved in enzyme activation by hydrogen peroxide), as shown in Figure 6. The presence of specific catalytic residues [82] allowed us to classify the nine members of family AA2. Firstly, three short MnPs (Figure 6A-C) characterized both by the presence of a manganese oxidation site formed by two glutamates and one aspartate at the internal heme propionate region, and by a shorter C-terminal tail than that of long and extralong MnPs [6]. Secondly, four LiPs (Figure 6D-G) containing a 174-Trp residue exposed to the solvent responsible for oxidation of high-redox-potential aromatic compounds. Thirdly, one VP (Figure 6H) including both a catalytic Trp residue exposed to the solvent and a manganese oxidation site; fourth, one atypical VP (Figure 6I) differing from VPs in one of the three acidic residues of the manganese oxidation site. A partial sequence for the first 138 amino acids of the *N*-terminal end of an additional putative class II peroxidase was also identified and could be hypothetically annotated as a LiP6. The above set of AA2 peroxidases identified in *P. cinnabarinus* is close to that identified in *Trametes versicolor* (in both cases consisting of MnP, LiP, VP and atypical-VP) [84], although the total number of sequences is lower in *Pycnoporus*. Two genes encoding heme peroxidases of a recently discovered superfamily of heme-thiolate peroxidases (HTP) [85] were also identified in *P. cinnabarinus*.

These peroxidases are widely distributed in fungal genomes, including those from soft-rot, brown-rot and white-rot fungi [6, 84, 86, 87]. However, only a few of them have so far been studied, with those from *Leptoxyphium fumago* and *Agrocybe aegerita* being the best characterized. They are known to catalyze halogenation reactions and to possess catalase, peroxidase and peroxygenase activities [88]. Consequently, similar reactions are expected to be catalyzed by the HTPs identified in the *P. cinnabarinus* genome sequence.

All *lip* and *mnp* genes except MnP2 and LiP6 were also found in the cDNA library (Additional file 3: Table S3). The *mnp* genes present lengths of 1.4–1.5 kb, (Additional file 7: Table S5) and and count 4–6 introns according to gene. The two genes encoding VP and the four LiPs showed relatively similar sizes (about 1.45 kb) and were interrupted by six introns, for coding sequences of similar length (about 1.1 kb).

Considering the analysis of the intron/exon structure, a division of family AA2 into several subgroups could be proposed. *vp* and *lip* genes share a similar structural organization and form one group (Additional file 8: Figure S2 A), whereas *mnp* genes are a more heterogeneous group in terms of gene structure, i.e. exons 2 and 3 of the *mnp2/mnp3* pair merge into a single exon in *mnp1* while exons 3, 4 and 5 of the *mnp1/mnp2* pair correspond to a single exon in *mnp3*. Finally, *atypical-vp* gene was totally different in length (1728 bp), number and structure of exons/introns compared with the other class II peroxidase genes analyzed (Additional file 8: Figure S2 A).

In the genome of *P. cinnabarinus,* we noted that some class II peroxidase genes were grouped on the same scaffold, forming a cluster of peroxidases. This was the case for *mnp3, lip1, lip2* and *lip3* genes, each separated by about 2 kb and oriented in the same transcriptional direction on the 184983 scaffold. Johansson and Nyman [89] had already described in *T. versicolor*, a similar cluster of three genes encoding two LiPs (LPGIII, LPGIV) and one MnP (MPG1) in a genomic region of 10 kb, oriented in the same transcriptional direction and separated by approximately 2.4 kb. In addition, the intron/exon organization of these *T. versicolor* genes pointed to a similar structure for the two *LPGIII* and *LPGIV* (about 1470 bp in length, including six introns), whereas the *MPG1* gene was slightly different (1400 bp interrupted by five introns).

**Figure 7:**
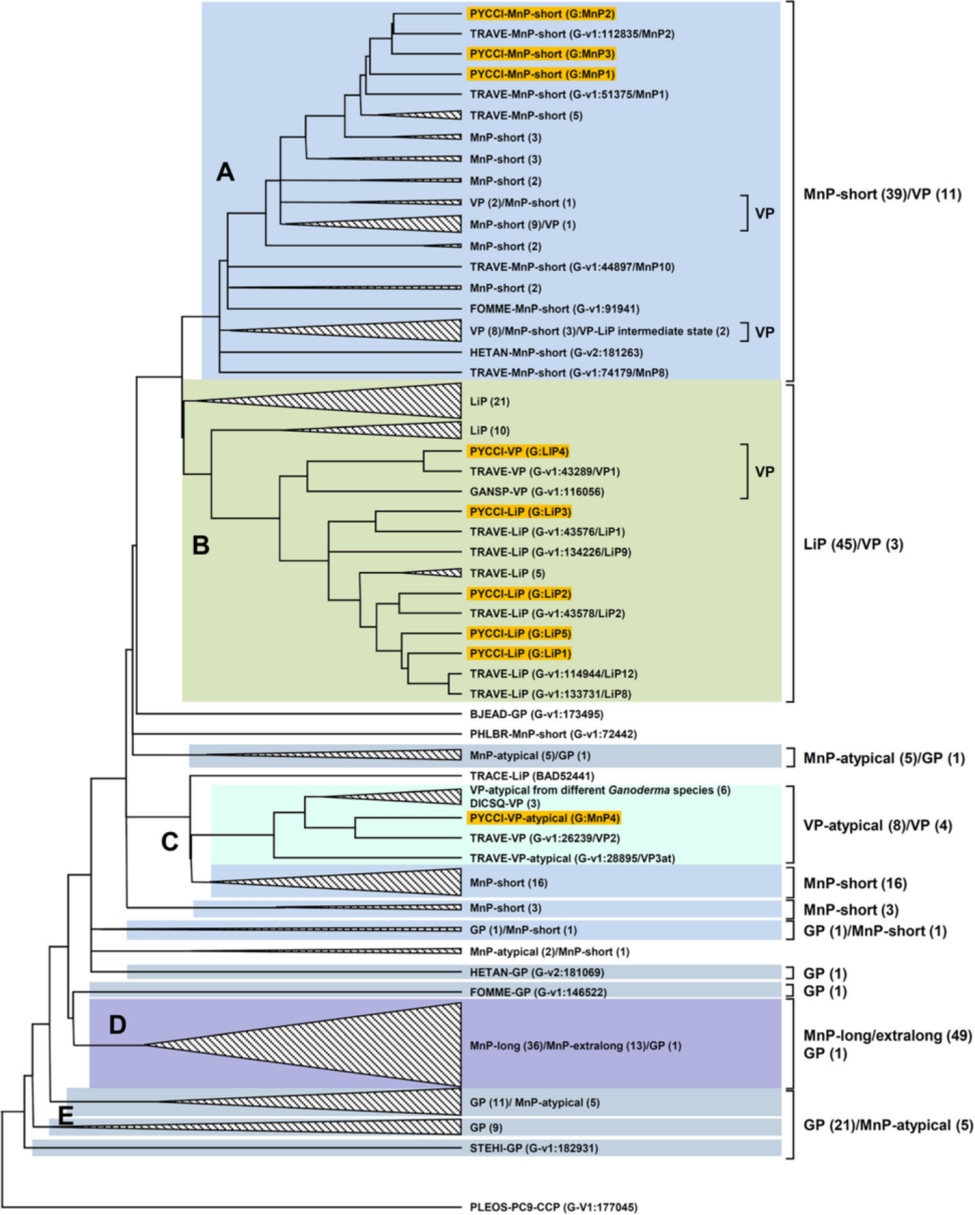
**Dendrogram of 223 sequences of class-II basidiomycete heme peroxidases (AA2) showing the position of nine sequences from the** ***P. cinnabarinus*** **genome (orange background).** Evolutionary analysis was performed with MEGA5 using Poisson distances and an unweighted pair group method with arithmetic mean clustering. The cytochrome *c* peroxidase from *P. ostreatus*, monokaryon PC9, was used to root the tree (http://phylobench.vital-it.ch/raxml-bb/). The dendogram was used to illustrate the clustering of sequences (clusters A to E). Clusters with no *P. cinnabarinus* sequences included were collapsed. Most of the sequences were obtained from the analysis of fungal genome sequences deposited at the US Department of Energy Joint Genome Institute (JGI), with the rest collected from GenBank [86]. Fungal abbreviations are as follows: BJEAD, *Bjerkandera adusta* (JGI); DICSQ, *Dichomitus squalens* (JGI); FOMME, *Fomitiporia mediterranea* (JGI); GANSP, *Ganoderma* sp. (JGI); HETAN, *Heterobasidion annosum* (JGI); PHLBR, *Phlebia brevispora* (JGI); PYCCI, *Pycnoporus cinnabarinus*; STEHI, *Stereum hirsutum* (JGI); TRACE, *Trametopsis cervina*; and TRAVE, *Trametes versicolor* (JGI). Other fungal species with peroxidase sequences included in the collapsed clusters are: *Agaricus bisporus* (JGI), *Auricularia delicata* (JGI), *Bjerkandera sp* (JGI), *Ceriporiopsis rivulosa*, *Coprinellus disseminatus*, *Coprinopsis cinerea* (JGI), *Fomitopsis pinicola* (JGI), *Ganoderma applanatum*, *Ganoderma australe*, *Ganoderma formosanum*, *Ganoderma lucidum*, *Gelatoporia subvermispora* (JGI), basidiomycete IZU-154, *Laccaria bicolor* (JGI), *Lentinula edodes*, *Phanerochaete chrysosporium* (JGI), *Phanerochaete sordida*, *Phlebia radiata*, *Pleurotus eryngii*, *Pleurotus ostreatus* (JGI), *Pleurotus pulmonarius*, *Pleurotus sapidus*, *Punctularia strigosozonata* (JGI), *Rhodonia placenta* (JGI), *Spongipellis* sp., *Taiwanofungus camphoratus*, *Wolfiporia cocos* (JGI).

After analyzing the recently-sequenced *T. versicolor* genome sequence [6], we identified an additional *lip* gene (1441 bp in length, including six introns) 6.8 kb upstream of the above sequences, completing the same cluster of three *lip* and one *mnp* genes as that observed in *P. cinnabarinus*. Compared with other class II peroxidases (see the dendrogram in Figure 7), these sequences appear closely related to those located at the same positions in the cluster identified in *P. cinnabarinus* (*mnp3*/*mnp2*, *lip1*/*lip12*, *lip2*/*lip2* and *lip3*/*lip1* in *P. cinnabarinus*/*T. versicolor*). The co-localization of these genes in both genomes suggests they may occupy a large orthologous genomic region that has been preserved in these two closely-related species sharing a common ancestor [84]. *htp1*, *htp2* and *lip6* genes also clustered on scaffold 184962 at 7.9 kb (*htp1* and *htp2*) and 34.1 kb (*htp2* and *lip6*) apart (Additional file 8: Figure S2 B). Similarly, two of the three *htp* genes identified in *T. versicolor*, only 1.2 kb apart, form a cluster on scaffold 12 but are arranged in the same transcriptional direction, whereas those from *P. cinnabarinus* are found in a transcriptionally convergent orientation, and the nearest class II gene is located 64 kb away. This suggests that unlike what is observed for *mnp3, lip1*, *lip2* and *lip3* genes, the organization of *htp* genes does not appear to be conserved between these two species of the core polyporoid clade. Almost all of these peroxidase genes were transcribed, as they were recovered in the *P. cinnabarinus* BRFM137 cDNA library (Additional file 3: Table S3). The cloning of partial *lip*-like genes is described in Additional file 9: Data S2 [90–93].

Figure 7 provides a dendrogram showing sequence relationships between 223 protein sequences of basidiomycete class II peroxidases [6], including those identified in the genome of *P. cinnabarinus*. Five peroxidase groups can be distinguished. Cluster A consists of 39 short MnPs where the three *P. cinnabarinus* MnPs appear closely related to seven of the 12 short MnPs identified in the *T. versicolor* genome sequence [84], and relatively distant from the 11 VPs from *P. eryngii*, *P. ostreatus*, *P. pulmonarius*, *P. sapidus*, *B. adusta* and *Spongipellis* sp. also included in this cluster. A well-defined cluster B contains all the LiP (45) sequences, including the 4 LiPs from *P. cinnabarinus* closely related to the 10 LiPs identified in *T. versicolor*, as well as the only *P. cinnabarinus* VP grouped together with the two other VPs (from *T. versicolor* and *Ganoderma* sp.) contained in this cluster. Cluster C consists of 16 short MnPs, four VPs and seven atypical VPs, plus the only atypical VP identified in *P. cinnabarinus* which is grouped together with VPs and atypical VPs from other species (*T. versicolor*, *D. squalens* and different *Ganoderma* species), all of them clustered together with *P. cinnabarinus* within the core polyporoid clade. The clearly-differentiated cluster D is composed of intermixed long and extralong MnPs absent in *P. cinnabarinus* and characterized by the presence of 10–20 and 20–30 extra amino acid residues at the C-terminal end, respectively (compared with short MnPs), and by containing one more disulfide bridge than LiPs, short MnPs and VPs (and their atypical variants). Different groups of generic peroxidases (GP) and atypical MnPs (not identified in *P. cinnabarinus*) are located next to the root of the dendrogram in the cluster D.

#### Descriptions of other AA proteins involved in ligninolysis

Other putative AA proteins produce the hydrogen peroxide necessary for the catalytic cycle of hydrogen peroxide-dependent fungal peroxidases (LiP, MnP, VP). The ability of hydrogen peroxide to generate hydroxyl radicals (OH^•^) also points to another role of hydrogen peroxide in the biodegradation of wood, where these hydroxyl radicals (OH^•^) could initiate the attack of lignocellulose [94]. For these reasons, research into hydrogen peroxide-producing enzymes – especially AA3_2 (aryl alcohol oxidases) and AA5_1 (glyoxal oxidases) – has surged. The subfamily AA5_1 contains glyoxal oxidases (called Glox) and copper radical oxidases (called Cro), which are enzymes related to glyoxal oxidases containing conserved active site residues but that diverge in terms of other structural features [95]. *In P. cinnabarinus*, seven AA5_1 enzymes have been identified in *P. cinnabarinus* BRFM137, including three glyoxal oxidases *stricto sensu* and four “radical copper oxidases” (Additional file 10: Table S6). Furthermore, these three *glox* and four *cro* were also expressed in the cDNA library.

Glox and Cro encoding genes (AA5_1) have diverse characteristics in *P. cinnabarinus*. The gene sizes ranged from 1.85 to 4.45 kb and were interrupted by one to 22 introns, corresponding to coding sequences ranging from 1.6 to 3 kb (Additional file 11: Table S7). The structure of the gene called *cro2* stands out 19 from the others, with a large number (22) of introns. In contrast, the sequences identified as *glox sensu stricto* share comparable size (1.85 kb) and structure (three introns) and form a homogeneous group. Based on the analysis of the intron/exon structure of each *Pycnoporus* AA5_1 encoding gene (Additional file 12: Figure S3 A), we could propose dividing AA5_1 into three subgroups corresponding to: *(i)* the *glox* sequences, which had strong intron position homology, *(ii)* the *cro1*, *cro3* and *cro4* sequences, and *(iii)* the very different *cro4* sequence. Moreover, the three *glox* genes formed a cluster oriented in the same transcriptional direction and grouped on the same scaffold, with *glox2* and *glox3* separated by only 1.1 kb (Additional file 12: Figure S3 B). This type of organization has also been found for the genes named *cro3*, *cro4* and *cro5* in *P. chrysosporium* ([95]; Additional file 13: Data S3 [95–97]). Additional file 14: Figure S4 and Additional file 15: Data S4 report the structural comparison between the Glox1 protein sequence from *P. cinnabarinus* and that of Gaox (PDB reference 1GOG) [97].

### Secretome analyses and lignocellulosic degradation

Several recent studies have shown that the diversity (number and type) of hemicellulolytic and ligninolytic enzymes or isoenzymes produced by basidiomycetes depends on substrate used and mode of cultivation (liquid culture (LC) or solid-state fermentation (SSF)) [98–102]. Agro-residues such as fruit peels (banana, mandarin, melon, peach and apple peels) are rich in cellulose, hemicellulose, lignin, soluble sugars and aromatic compounds, and were found to be substrates favoring the production of glycoside hydrolases and laccases in white-rot basidiomycetes [99]. Lignocellulosic residues such as straw, bran and wood chips favor the peroxidase production by most basidiomycetes [99]. LC promotes the production of laccases and hydrolases while SSF promotes the production of peroxidases, including MnPs [101, 102]. We thus ran several *P. cinnabarinus* BRFM137 cultures via both LC and SSF in presence of simple or complex “natural” substrates to compositionally analyze the corresponding secretomes (Additional file 16: Table S8).

**Figure 8:**
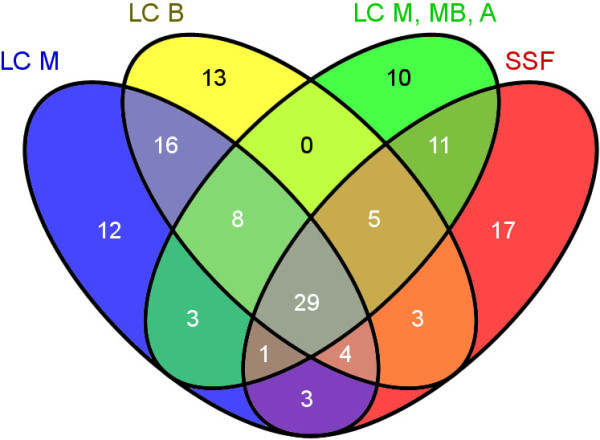
**Venn diagram showing CAZyme distributions among the** ***P. cinnabarinus*** **secretomes from different growth conditions.** LC: liquid culture, B: birchwood, M: maltose, M-MB-A: maltose + maize bran + Avicel, SSF: solid-state fermentation.

Analysis of the *P. cinnabarinus* secretomes detected 184 proteins in LC-M (maltose), 166 proteins in LC-B (maltose and micronized birchwood), 121 proteins in LC-M-MB-A (maltose, maize bran, Avicel), and 139 proteins in SSF cultures. Most of the secreted proteins in our culture conditions consisted of carbohydrate-active enzymes (CAZymes), which represented 55% and 52% of the total proteins detected in LC-M-MB-A and SSF, respectively, and 41% and 47% in LC-M and LC-B, respectively (Additional file 16: Table S8). CAZyme distributions were compared according to the different cultures conditions (Figure 8). Interestingly, the LPMOs of family AA9 were only identified in the conditions including complex substrates, and no AA9 protein was found in the control condition with maltose. Moreover, different AA9 proteins were produced in response to different growth conditions. For instance, three AA9 proteins were produced only with birchwood, whereas two different AA9 proteins were identified in cultures with maize bran and Avicel. This result indicates that there is a differential regulation of the LPMO genes that is dependent on growth substrates and/or on temporal scale. Indeed, the AA9-encoding genes may also be constrained by strict short expression during substrate-supported fungal growth. In recent studies, a preponderance of AA9 was produced exclusively in sugar beet pulp conditions [103]. The detailed distribution of the (hemi)cellulolytic and ligninolytic proteins detected in secretomes is described in Additional file 16: Table S8. Interestingly, all the representatives of the ligninolytic AA families were identified in these conditions, although with different distribution patterns depending on growth conditions (Additional file 17: Table S9). Three AA1_1 laccases (scf184817_g29; scf185007_g100; scf185007_g107) were identified in all conditions studied here, demonstrating that these enzymes are widely and constitutively produced by the fungus. Contrary to laccases, members belonging to the class II peroxidases (AA2) were only identified in the secretomes from SSF cultures (one Lip and one MnP) and in LC-M (atypical-VP). Despite the major role of family peroxidases AA2 in lignin degradation, no AA2 protein was detected in the conditions using the hardwood substrate (birchwood). The class II peroxidases could be constrained by a fine-tuned regulation or, alternatively, be not produced in our growth conditions. The expression and regulation of class II peroxidase-encoding genes depend on environmental signals such as concentration of carbon and nitrogen, exposure to metal ions and xenobiotics, temperature shock, and daylight [104].

A number of cellulolytic enzymes were produced in all conditions studied. For instance, secretomes contained members of the families GH3, GH6, GH7 and GH12, which are principally involved in cellulose breakdown. However, the endo-β-1,4-glucanases of the subfamily GH5_5 were only produced when birchwood was used in the culture medium. We also identified a number of xylan-degrading enzymes produced only in the LC-M-MB-A (maltose, maize bran, Avicel), including members of families CE1, CE15, GH3, GH5, GH10. Moreover, family CE1 members were only found when maize bran was used in the cultures. Among the known activities in the CE1 family, feruloyl esterase activity mobilizes key enzymes acting on ferulic and diferulic acid bridges embedded in the hemicellulose from plant cell walls [105]. Maize fiber xylan features among the most complex heteroxylans and is highly substituted by feruloylated branches yielding a large in ferulic acid content of up to 3% of the dry mass [106]. Thus, the breakdown of this substrate required varied enzymes, as suggested by the diversity of xylanolytic enzymes produced by *P. cinnabarinus* in presence of maize bran.

### Protein secretion and glycosylation pathways

The main lignocellulolytic enzymes of *P. cinnabarinus* are extracellular, and the proteins are secreted and processed during secretion by the secretion and glycosylation systems of the fungus. Analysis of the genes involved in protein secretion and glycosylation shows that *P. cinnabarinus* contains the entire machinery needed for protein secretion via the classical secretory pathway (Additional file 18: Table S10 and Additional file 19: Table S11, respectively). Transport of secretory proteins is expected to take place both via a pathway dependent on a signal recognition particle (SRP) and via an SRP-independent pathway, as genes for both pathways were identified. Protein transport from one compartment to the next in the secretory pathway is carried out by various protein complexes, such as the COPI/COPII complexes, Transport Protein Particule (TRAPP) complex and the exocyst complex (Additional file 18: Table S10).

The genome contains homologs of subunits in these complexes and indicates that the complexes are highly conserved in *P. cinnabarinus*. We also screened for V- and T-SNAREs (Soluble NSF Attachment Protein Receptors) in the genome and for secretion-related GTPases. Both the SNARE proteins and secretion-related GTPases are expected to function at discrete steps in the secretory pathway, and for most proteins we were able to identify a bi-directional best hit, indicating conservation of these functions, probably at the same step along the secretory pathway.

The endoplasmic reticulum (ER) is an important organelle that harbors the enzymes required for proper folding of secretory proteins. *P. cinnabarinus* is fully equipped with the enzymes needed for protein folding and disulfide bridge formation (Additional file 18: Table S10). The machinery to deal with misfolded or unfolded proteins (the Unfolded Response Pathway (UPR)) is also conserved, although we were unable to identify a clear ortholog of the Hac1/XBP1 transcription factor in the *P. cinnabarinus* genome. Hac1/HacA (in fungi) or Xbp1 (mammalian cells) is a bZIP transcription factor that is uniquely activated by an unconventional splicing event mediated by Ire1p (acting as sensor and endonuclease) and Trl1p (acting as ligase) [107]. The presence of proteins involved in Hac1 activation, such as the sensor (Ire1p) and the tRNA ligase (Trl1p), in the *P. cinnabarinus* genome suggests that this same UPR mechanism via HacA activation is also present in *P. cinnabarinus*. The removal of misfolded protein via the ER-associated degradation (ERAD) system, which targets misfolded proteins for degradation in the proteasome, is conserved, since we identified orthologous proteins to the ERAD and proteasome (Additional file 18: Table S10).

We also analyzed the presence of genes related to post-translational modifications in the secretory system including protein *N*- and *O*-glycosylation as well as glycosylphosphatidylinositol (GPI)-anchor biosynthesis, (Additional file 19: Table S11). The biosynthetic genes required for the formation of nucleotide sugar GDP-mannose, UDP-glucose, UDP-*N*-acetylglucosamine and UDP-galactose together with transporter to localize the nucleotide sugars in the ER or Golgi lumen were identified. The genes encoding the proteins for stepwise synthesis of the dolicholphosphate-linked oligosaccharide (ALG genes; asparagine (N)-linked- glycosylation) as well as the transfer of the oligosaccharide to asparagine residues (OST-complex) are conserved and found in the genome of *P. cinnabarinus*. Similarly, genes homologous to the attachment of the mannose residue to serine or threonine residues (*O*-linked glycosylation), which are carried out by protein mannosyl transferase (PMT), are also conserved. Like in other fungi, *P. cinnabarinus* has a genome that contains multiple PMT homologs. Glycosylphosphatidylinositol (GPI)-anchor biosynthesis and transfer of the pre-assembled GPI anchor also takes place in the ER. Most of the genes involved in GPI-anchor biosynthesis were identified. The genes encoding Golgi-localized proteins that are involved in outer chain elongation (Och1p/Mnn9p mannosyltransferase complexes) are not present in the *P. cinnabarinus* genome.

The genes encoding proteins that are expected to add the second and third mannosyltransferase to O-chains are present, but genes homologous to α-1-3-mannosyltransferase that add the fourth or fifth mannose to O-chains were not identified. Thus the post-transcriptional glycosylation events in the Golgi appear to be severely curtailed in *P. cinnabarinus* to much the same extent as previously reported for the basidiomycete *S. commune* [61, 108]. Galactofuranosylation is a type of modification found on glycoproteins in *Aspergillus* species [109], but the genes involved in this process are absent in *P. cinnabarinus*. This raises prospects for using *P. cinnabarinus* to produce pharmaceutical proteins, as the glycostructures (*N-* and *O-*chains) have a mammalian-like structure and are devoid of the highly antigenic galactofuranose residues found in expression hosts such as *A. niger* see [109] for review.

### Mating-type loci and their genes in *P. cinnabarinus*

In the past, the fungal lifecycle of *P. cinnabarinnus* was studied in order to select monokaryotic lines with characteristics specifically tied to lignocellulose degradation [17]. *Pycnoporus* species are heterothallic Agaricomycetes with two mating type loci controlling the fungal lifecycle [8, 110]. One mating type locus (*A* locus) in the tetrapolar Agaricomycetes encodes two types of homeodomain transcription factors (HD1 and HD2) in divergently-transcribed gene pairs, whereas the other (*B* locus) contains genes for pheromones and pheromone precursors, respectively [111, 112].

#### *The*A *mating type locus*

HD1 and HD2 mating type proteins from *P. chrysosporium* (ADN97192.1, ADN97171.1) were successfully used to screen the *Pycnoporus* EST contigs. *Pycnoporus*, like other basidiomycetes [110], has at least one *HD1* and one *HD2* gene for homeodomain transcription factors. The HD1 protein a1-1 deduced from contig > GCTO4WP02F0TDF.f.pc.1 dna:contig contig::GCTO4WP02F0TDF.f.pc.1:1:2252:1 is 495 aa long. Its *N*-terminal domain is related to the *N*-terminal of mating type proteins from other species (Additional file 20: Figure S5 A) and is expected to act in heterodimerization with compatible HD2 proteins while discriminating HD2 proteins from the same mating type [111]. The two classes of homeodomain proteins encoded in basidiomycete mating type loci are defined by their distinct homeodomain sequences [113]. HD1 proteins have a TALE-class homeodomain with three extra amino acids in-between Helix I and Helix II of the three-helical DNA-binding domain. Some amino acid exchanges in the conserved DNA-recognition motif (WFxNxR) in Helix III are tolerated [112]. In the *Pycnoporus* a1-1 protein, the position of the HD1 homeodomain is only recognized by sequence alignment with related HD1 proteins from other species (Additional file 20: Figure S5 A). The DNA-recognition sequence in Helix III is degenerated and Helix II has undergone a deletion. Previous research failed to find the expected conserved HD1 motif in respective proteins of *Postia placenta* [4]. We note from other species that a defective HD1 homeodomain does not inevitably cause loss-of-function in mating type regulation provided that the HD2 homeodomain in a HD1-HD2 heterodimer continues to function [114].

A *HD2* gene for the 569 aa-long protein a2-1 was found on > GCTO4WP02F01PN.f.pc.1 dna:contig contig::GCTO4WP02F01PN.f.pc.1:1:2027:1. The protein has a classical 60 amino acid-long homeodomain with all invariant residues in the DNA-binding motif which is highly sequence-conserved with HD2 mating type proteins from other Agaricomycetes (Additional file 20: Figure S5 B).

Interestingly, contig GCTO4WP02F0TDF.f.pc.1 contains not only the full-length coding sequence of protein a1-1 (>scf185007.g8) but also, downstream on the opposite strand, the 3-terminal half of gene *β-fg* for an unknown fungal protein (>scf185007.g7), which in most Agaricomycetes flanks one side of the homeodomain transcript factor locus [115]. At the other side of the loci, a *mip* gene for a mitochondrial intermediate peptidase is usually present [116]. *P. chrysosporium* and *P. placenta* differ from other analyzed Agaricomycetes in the relative order of their single *HD1* gene to *mip* and *β-fg*. These are the two species where *HD1* gene neighbors *β-fg* and not *mip* and is transcribed in the same direction as *mip*, suggesting that there has been an inversion of the mating type locus [115, 117]. Contig GCTO4WP02F0TDF.f.pc.1 indicates that *Pycnoporus* is another species with this same inverted arrangement. *P. chrysosporium* (Phanerochaetaceae) and *P. placenta* (Fomitopsidaceae), like *Pycnoporus* (Coriolaceae), belong to the Polyporales, and an inversion event early in evolution is likely [118].

#### *The*B *mating type locus*

The bipolar *P. chrysosporium* contains five genes for pheromone receptors, three of which cluster together in a locus orthologous to the *B* mating type locus of tetrapolar species, whereas two others belong to the still-unexplored non-mating-type G protein-coupled transmembrane receptors of the Agaricales [115, 117]. The five *P. chrysosporium* proteins were used to screen the *Pycnoporus* EST contigs, and five hits were found. Three models contained full-length (*PciSTE3.2*, *PciSTE3.3*) or nearly complete (*PciSTE3.4*) ORFs for G protein-coupled transmembrane receptors (Additional file 21: Figure S6). The other two contained a 5′ half of a gene (*PciSTE3N*) and a 3′ half of a gene (*PciSTE3C*), respectively, and it is possible that these two EST contigs present the same gene (Additional file 21: Figure S6). Sequence analysis of the nearly- complete proteins using ClustalW for alignment (http://www.clustal.org/clustal2/), GeneDoc (http://www.psc.edu/biomed/genedoc/) for manual corrections, and the neighbor-joining function in MEGA4 software [119] indicates that PciSTE3.4 groups with the two non-mating-type G protein-coupled transmembrane receptors of *P. chrysosporium* (Additional file 22: Figure S7 A), whereas *PciSTE3.2* and *PciSTE3.3* cluster with the B-mating-type-orthologous receptors, respectively (Additional file 22: Figure S7 B,C). This finding suggests that *P. cinnabarinus*, like other tetrapolar Agaricales, has B-mating-type-specific and non-mating-type genes for pheromone receptors [112, 115]. We also analyzed the *N*-terminal ends and the *C*-terminal ends of the proteins separately and together with the protein halves deduced from the incomplete EST contigs GCTO4WP02FNFO2.f.pc.1 and GCTO4WP02F7KNS.f.pc.1, respectively. In both phylogenetic trees, the partial pheromone receptors group with PciSTE3.2 and with the B orthologous PchSTE3.2 of *P. chrysosporium*, which is evidence that the sequences may come from the same gene. As in several other species [112, 115], there are thus at least three expressed paralogous candidate genes for B-mating-type function in *P. cinnabarinus*.

Pheromone precursors are short peptide chains of up to about 100 aa and the mature pheromones are 9 to 14 aa-long peptides, which are difficult to find in BLAST searches even at lowest stringency due to strongly divergent sequences [112, 120]. Searches starting with the five identified *P. chrysosporium* pheromone precursor sequences [112, 117] were unsuccessful, but sequences from *Serpula lacrymans* (http://genome.jgi-psf.org/SerlaS7_3_2/SerlaS7_3_2.home.html) and cross-searches with the detected *P. cinnabarinus* pheromone precursors identified a total of seven potential 39-to-65-aa-long pheromone precursors. All possess the typical CAAX (cysteine-aliphatic-aliphatic-any amino acid) motif at the *C*-terminus and a MDA/DF-motif at the *N*-terminus (Additional file 23: Figure S8). Three are very distinct in sequence, as is typical for B-mating-type pheromone precursors, whereas four others share more similarity, resembling the precursors of presumed non-mating-type pheromone-like peptides [112, 115].

## Conclusions

The *P. cinnabarinus* genome contains the genes encoding the full enzymatic portfolio for lignin degradation, notably peroxidases and numerous auxiliary enzymes for the generation of hydrogen peroxide. Several laccase-encoding gene models and AA2 peroxidases (MnP, LiP or VP) were identified in *P. cinnabarinus*. A large number of these genes are expressed as they have been detected as transcripts in the cDNA library. Furthermore, secretome analysis showed effective and differential secretion of several peroxidases, copper radical oxidases, and aryl alcohol, glucose and pyranose oxidases under our culture conditions. These genes structurally organize into a set of clusters and intron/exon position homologies. The physical proximity of these genes (*lac, lip, mnp, glox*) suggests that this organization may result from chromosomal rearrangements such as local duplications. Furthermore, the different isoenzymes annotated in *P. cinnabarinus* evidenced high diversity in terms of primary sequence and predicted biochemical characteristics. In *P. cinnabarinus*, post-transcriptional glycosylation capabilities appear reduced to the strict minimum, making it a promising candidate for heterologous protein production in biotechnological applications. In conclusion, *P. cinnabarinus* is shown to be an outstanding and representative model white-rot fungi for studying the enzyme machinery involved in the degradation and/or transformation of lignocellulosic materials.

## Electronic supplementary material

Additional file 1: Table S1: List of annotated genes in *P. cinnabarinus*. (XLS 1 MB)

Additional file 2: Table S2: List of the lignocellulolytic repertoire encoding-genes in *P. cinnabarinus* BRFM137. (DOCX 28 KB)

Additional file 3: Table S3: Annotation of the lignin oxidoreductases predicted from the genome of *P. cinnabarinus* BRFM137. (DOCX 15 KB)

Additional file 4: Table S4: Characteristics of laccase genes from *P. cinnabarinus* BRFM137. (DOCX 14 KB)

Additional file 5: Figure S1: Molecular characterization of *P. cinnabarinus* BRFM137 laccase genes. (DOCX 76 KB)

Additional file 6: Data S1: Multiplicity of fungal laccase encoding-genes and gene organization 69 70 71 72 73 74 75 76 77 78 79 80. (DOCX 17 KB)

Additional file 7: Table S5: Characteristics of peroxidase genes from *P. cinnabarinus* BRFM137. (DOCX 14 KB)

Additional file 8: Figure S2: Molecular characterization of peroxidase genes (A) and schematic organization of peroxidase gene clusters on genomic DNA from *P. cinnabarinus BRFM137* (B). (DOCX 64 KB)

Additional file 9: Data S2: Amplification of lip-like genes in white-rot fungi 90 91 92 93. (DOCX 15 KB)

Additional file 10: Table S6: Annotation of the AA5_1 proteins predicted from the genome of *P. cinnabarinus* BRFM137. (DOCX 14 KB)

Additional file 11: Table S7: Characteristics of AA5_1 genes from *P. cinnabarinus* BRFM137. (DOCX 14 KB)

Additional file 12: Figure S3: Molecular characterization of AA5_1 genes (A) and schematic organization of glyoxal oxidase gene clusters on genomic DNA from *P. cinnabarinus* BRFM137 (B). (DOCX 50 KB)

Additional file 13: Data S3: Copper radical oxidases of *P. chrysosporium* [95–97] *.* (DOCX 16 KB)

Additional file 14: Figure S4: Modeling of the active site of the theoretical Glox1 predicted in *P. cinnabarinus* BRFM137 and comparison with galactose oxidase from *Dactylium dendroides*. (DOCX 701 KB)

Additional file 15: Data S4: Structural comparison between Glox1 and Gaox proteins [97]. (DOCX 14 KB)

Additional file 16: Table S8: List of lignocellulolytic enzymes identified in the secretomes of *P. cinnabarinus* BRFM137 in different growth conditions. (XLSX 30 KB)

Additional file 17: Table S9: List of unique and common genes identified in the different growth conditions. (DOCX 18 KB)

Additional file 18: Table S10: *P. cinnabarinus* genome annotation related to protein secretion pathways. (DOCX 48 KB)

Additional file 19: Table S11: *P. cinnabarinus* genome annotation related to protein glycosylation pathways. (DOCX 32 KB)

Additional file 20: Figure S5: Alignments of the N-terminal regions of HD1 (A) and HD2 mating type proteins (B) of *Pycnoporus* and other Agaricomycetes. (DOCX 182 KB)

Additional file 21: Figure S6: Alignment of sequences of putative G protein-coupled transmembrane pheromone receptors deduced from EST contigs of *P. cinnabarinus* (Pci) and sequences of pheromone receptors of *P. chrysosporium* (Pch; for nomenclature, see James *et al.* [117]). (DOCX 2 MB)

Additional file 22: Figure S7: Neighbour-joining trees (bootstrap values: 500) of sequences of A. (nearly) complete, B. N-terminal and C. C-terminal halves of pheromone receptors of *P. cinnabarinus* and *P. chrysosporium* (for nomenclature see James *et al.* [117]). The classification in non-mating type and B orthologs follows the analysis of Niculita-Hierzel *et al.* [115]. (DOCX 38 KB)

Additional file 23: Figure S8: Alignment of sequences of putative B mating type pheromone precursors (Ph1 and Ph2) and of putative precursors for non-mating-type pheromone-like peptides (Phl1 to Phl3). (DOCX 42 KB)
