# Supplementary material for: The genome of the white-rot fungus Pycnoporus cinnabarinus: a basidiomycete model with a versatile arsenal for lignocellulosic biomass breakdown
Source: BMC Genomics. 2014 Jun 18;15:486. doi: 10.1186/1471-2164-15-486 (PMC4101180; doi:10.1186/1471-2164-15-486)
Supplement: Supplementary file 2 — Additional file 2: Table S2: List of the lignocellulolytic repertoire encoding-genes in P. cinnabarinus BRFM137. (DOCX 28 KB) [file 12864_2014_6245_MOESM2_ESM.docx]

**Additional file 2: Table S2. List of the lignocellulolytic repertoire encoding-genes in *P. cinnabarinus* BRFM137.**

| **ProteinID** | **Defline** | **Description** |
| --- | --- | --- |
| scf185007_g107 | Laccase | AA1_1 |
| scf185007_g100 | Laccase | AA1_1 |
| scf184817_g29 | Laccase | AA1_1 |
| scf184851_g84 | Laccase | AA1_1 |
| scf184918_g4 | Laccase | AA1_1 |
| scf184857_g29 | Multicopper oxidase | AA1_dist |
| scf184845_g66 | Ferroxidase | AA1_2 |
| scf184473_g24 | Class II peroxidase | AA2 |
| scf184983_g20 | Class II peroxidase | AA2 |
| scf184569_g58 | Class II peroxidase | AA2 |
| scf184993_g4 | Class II peroxidase | AA2 |
| scf184983_g21 | Class II peroxidase | AA2 |
| scf184983_g22 | Class II peroxidase | AA2 |
| scf184969_g43 | Class II peroxidase | AA2 |
| scf184983_g23 | Class II peroxidase | AA2 |
| scf184946_g21 | Class II peroxidase | AA2 |
| scf184962_g60 | Class II peroxidase | AA2 |
| scf185013_g1 | Iron reductase domain / Cellobiose dehydrogenase | AA8-AA3_1 |
| scf184611_g7 | Aryl-alcohol oxidase | AA3_2 |
| scf185002_g8 | Aryl-alcohol oxidase | AA3_2 |
| scf184746_g13 | Aryl-alcohol oxidase | AA3_2 |
| scf184817_g4 | GMC oxidoreductase | AA3_2 |
| scf184817_g12 | GMC oxidoreductase | AA3_2 |
| scf184986_g5 | GMC oxidoreductase | AA3_2 |
| scf184863_g21 | GMC oxidoreductase | AA3_2 |
| scf184939_g39 | GMC oxidoreductase | AA3_2 |
| scf184611_g2 | GMC oxidoreductase | AA3_2 |
| scf184868_g6 | GMC oxidoreductase | AA3_2 |
| scf184803_g17 | Glucose oxidase | AA3_2 |
| scf184989_g54 | GMC oxidoreductase | AA3_2 |
| scf184986_g6 | GMC oxidoreductase | AA3_2 |
| scf184866_g17 | GMC oxidoreductase | AA3_2 |
| scf185002_g110 | GMC oxidoreductase | AA3_2 |
| scf184674_g5 | GMC oxidoreductase | AA3_2 |
| scf184594_g10 | GMC oxidoreductase | AA3_2 |
| scf185015_g104 | GMC oxidoreductase | AA3_2 |
| scf184830_g29 | GMC oxidoreductase | AA3_2 |
| scf185011_g8 | Alcohol oxidase | AA3_3 |
| scf184805_g45 | Alcohol oxidase | AA3_3 |
| scf184913_g13 | Pyranose oxidase | AA3_4 |
| scf184913_g15 | Pyranose oxidase | AA3_4 |
| scf185014_g43 | Copper radical oxidase | AA5_1 |
| scf184281_g9 | Copper radical oxidase | AA5_1 |
| scf185009_g14 | Copper radical oxidase | AA5_1 |
| scf184747_g41 | Glyoxal oxidase | AA5_1 |
| scf184747_g42 | Glyoxal oxidase | AA5_1 |
| scf184992_g45 | Copper radical oxidase | AA5_1 |
| scf184747_g48 | Glyoxal oxidase | AA5_1 |
| scf184992_g18 | Benzoquinone reductase | AA6 |
| scf185014_g31 | Iron reductase domain / Carbohydrate-Binding Module Family 1 protein | AA8-CBM1 |
| 184844_g129 | Auxilliary Activities Family 9 protein | AA9 |
| 185042_g102 | Auxilliary Activities Family 9 protein | AA9 |
| 184915_g56 | Auxilliary Activities Family 9 protein | AA9 |
| 184983_g12 | Auxilliary Activities Family 9 protein | AA9 |
| 184908_g125 | Auxilliary Activities Family 9 protein | AA9 |
| 184577_g3 | Auxilliary Activities Family 9 protein | AA9 |
| 184873_g30 | Auxilliary Activities Family 9 protein | AA9 |
| 184873_g32 | Auxilliary Activities Family 9 protein | AA9 |
| 184569_g69 | Auxilliary Activities Family 9 protein | AA9 |
| 184569_g70 | Auxilliary Activities Family 9 protein | AA9 |
| 184817_g21 | Auxilliary Activities Family 9 protein | AA9 |
| 184831_g7 | Auxilliary Activities Family 9 protein | AA9 |
| 185037_g14 | Auxilliary Activities Family 9 protein | AA9 |
| 184915_g57 | Auxilliary Activities Family 9 / Carbohydrate-Binding Module Family 1 protein | AA9-CBM1 |
| 184747_g17 | Auxilliary Activities Family 9 / Carbohydrate-Binding Module Family 1 protein | AA9-CBM1 |
| 184616_g3 | Carbohydrate-Binding Module Family 1 protein | CBM1 |
| 184980_g44 | Carbohydrate-Binding Module Family 1 / Carbohydrate Esterase Family 1 protein | CBM1-CE1 |
| 184980_g45 | Carbohydrate-Binding Module Family 1 / Carbohydrate Esterase Family 1 protein | CBM1-CE1 |
| 184969_g36 | Carbohydrate-Binding Module Family 1 / Carbohydrate Esterase Family 1 protein | CBM1-CE1 |
| 184856_g2 | Carbohydrate-Binding Module Family 1 / Carbohydrate Esterase Family 15 protein | CBM1-CE15 |
| 184817_g18 | Carbohydrate-Binding Module Family 1 / Glycoside Hydrolase Family 10 protein | CBM1-GH10 |
| 184654_g6 | Carbohydrate-Binding Module Family 1 / Glycoside Hydrolase Family 131 protein | CBM1-GH131 |
| 184983_g16 | Carbohydrate-Binding Module Family 1 / Glycoside Hydrolase Family 18 protein | CBM1-GH18 |
| 184727_g5 | Carbohydrate-Binding Module Family 1 / Glycoside Hydrolase Family 5 protein | CBM1-GH5_5 |
| 184921_g29 | Carbohydrate-Binding Module Family 1 / Glycoside Hydrolase Family 5 protein | CBM1-GH5_5 |
| 185007_g51 | Carbohydrate-Binding Module Family 1 / Glycoside Hydrolase Family 5 protein | CBM1-GH5_5 |
| 184961_g16 | Carbohydrate-Binding Module Family 1 / Glycoside Hydrolase Family 5 protein | CBM1-GH5_7 |
| 185014_g56 | Carbohydrate-Binding Module Family 1 / Glycoside Hydrolase Family 6 protein | CBM1-GH6 |
| 184884_g58 | Carbohydrate-Binding Module Family 12 protein | CBM12 |
| 184697_g12 | Carbohydrate-Binding Module Family 13 protein | CBM13 |
| 184697_g13 | Carbohydrate-Binding Module Family 13 protein | CBM13 |
| 184697_g14 | Carbohydrate-Binding Module Family 13 protein | CBM13 |
| 184712_g7 | Carbohydrate-Binding Module Family 13 protein | CBM13 |
| 184830_g3 | Carbohydrate-Binding Module Family 18 / Glycoside Hydrolase Family 16 protein | CBM18-GH16 |
| 184867_g21 | Carbohydrate-Binding Module Family 21 protein | CBM21 |
| 184940_g32 | Carbohydrate-Binding Module Family 21 protein | CBM21 |
| 184915_g9 | Carbohydrate-Binding Module Family 48 protein | CBM48 |
| 184982_g20 | Carbohydrate-Binding Module Family 48 / Glycoside Hydrolase Family 13 protein | CBM48-GH13_8 |
| 184753_g21 | Carbohydrate-Binding Module Family 50 protein | CBM50 |
| 184856_g1 | Carbohydrate Esterase Family 15 protein | CE15 |
| 185014_g131 | Carbohydrate Esterase Family 16 protein | CE16 |
| 184751_g9 | Carbohydrate Esterase Family 16 protein | CE16 |
| 184936_g15 | Carbohydrate Esterase Family 16 protein | CE16 |
| 184851_g92 | Carbohydrate Esterase Family 16 protein | CE16 |
| 184938_g40 | Carbohydrate Esterase Family 16 protein | CE16 |
| 184916_g7 | Carbohydrate Esterase Family 16 protein | CE16 |
| 184858_g50 | Carbohydrate Esterase Family 4 protein | CE4 |
| 184942_g36 | Carbohydrate Esterase Family 4 protein | CE4 |
| 184766_g19 | Carbohydrate Esterase Family 4 protein | CE4 |
| 185043_g20 | Carbohydrate Esterase Family 8 protein | CE8 |
| 184842_g13 | Distantly related to plant expansins | EXPN |
| 184842_g30 | Distantly related to plant expansins | EXPN |
| 185043_g66 | Distantly related to plant expansins | EXPN |
| 185016_g1 | Distantly related to plant expansins | EXPN |
| 184944_g3 | Distantly related to plant expansins | EXPN |
| 184652_g38 | Distantly related to plant expansins | EXPN |
| 184798_g41 | Distantly related to plant expansins | EXPN |
| 184798_g42 | Distantly related to plant expansins | EXPN |
| 184798_g43 | Distantly related to plant expansins | EXPN |
| 184785_g15 | Distantly related to plant expansins | EXPN |
| 184857_g56 | Glycoside Hydrolase Family 1 protein | GH1 |
| 185042_g12 | Glycoside Hydrolase Family 10 protein | GH10 |
| 184989_g9 | Glycoside Hydrolase Family 115 protein | GH115 |
| 184989_g20 | Glycoside Hydrolase Family 115 protein | GH115 |
| 184970_g119 | Glycoside Hydrolase Family 12 protein | GH12 |
| 184970_g120 | Glycoside Hydrolase Family 12 protein | GH12 |
| 184806_g33 | Glycoside Hydrolase Family 12 protein | GH12 |
| 185043_g112 | Glycoside Hydrolase Family 125 protein | GH125 |
| 185043_g104 | Glycoside Hydrolase Family 128 protein | GH128 |
| 185016_g6 | Glycoside Hydrolase Family 128 protein | GH128 |
| 185016_g7 | Glycoside Hydrolase Family 128 protein | GH128 |
| 184937_g23 | Glycoside Hydrolase Family 128 protein | GH128 |
| 184977_g63 | Glycoside Hydrolase Family 131 protein | GH131 |
| 184970_g84 | Glycoside Hydrolase Family 131 protein | GH131 |
| 184834_g43 | Glycoside Hydrolase Family 13 protein | GH13_1 |
| 184712_g6 | Glycoside Hydrolase Family 13 protein | GH13_1 |
| 184599_g1 | Glycoside Hydrolase Family 13 / Glycosyltransferase Family 5 protein | GH13_22-GT5 |
| 185008_g61 | Glycoside Hydrolase Family 13 protein | GH13_25 |
| 184992_g11 | Glycoside Hydrolase Family 13 / Carbohydrate-Binding Module Family 20 protein | GH13_32-CBM20 |
| 184883_g16 | Glycoside Hydrolase Family 13 protein | GH13_5 |
| 184970_g132 | Glycoside Hydrolase Family 15 protein | GH15 |
| 184935_g11 | Glycoside Hydrolase Family 16 protein | GH16 |
| 184935_g12 | Glycoside Hydrolase Family 16 protein | GH16 |
| 184829_g10 | Glycoside Hydrolase Family 16 protein | GH16 |
| 184829_g25 | Glycoside Hydrolase Family 16 protein | GH16 |
| 184989_g27 | Glycoside Hydrolase Family 16 protein | GH16 |
| 184911_g15 | Glycoside Hydrolase Family 16 protein | GH16 |
| 185015_g16 | Glycoside Hydrolase Family 16 protein | GH16 |
| 185015_g17 | Glycoside Hydrolase Family 16 protein | GH16 |
| 185015_g65 | Glycoside Hydrolase Family 16 protein | GH16 |
| 184645_g4 | Glycoside Hydrolase Family 16 protein | GH16 |
| 184392_g3 | Glycoside Hydrolase Family 16 protein | GH16 |
| 184902_g23 | Glycoside Hydrolase Family 16 protein | GH16 |
| 185009_g6 | Glycoside Hydrolase Family 16 protein | GH16 |
| 185009_g8 | Glycoside Hydrolase Family 16 protein | GH16 |
| 185009_g9 | Glycoside Hydrolase Family 16 protein | GH16 |
| 185016_g81 | Glycoside Hydrolase Family 16 protein | GH16 |
| 184970_g15 | Glycoside Hydrolase Family 16 protein | GH16 |
| 184746_g32 | Glycoside Hydrolase Family 16 protein | GH16 |
| 184801_g33 | Glycoside Hydrolase Family 16 protein | GH16 |
| 184941_g16 | Glycoside Hydrolase Family 16 protein | GH16 |
| 184989_g1 | Glycoside Hydrolase Family 16 protein | GH16 |
| 185015_g15 | Glycoside Hydrolase Family 16 protein | GH16 |
| 184902_g4 | Glycoside Hydrolase Family 16 protein | GH16 |
| 184829_g23 | Glycoside Hydrolase Family 16 | GH16-GH16 |
| 184980_g23 | Glycoside Hydrolase Family 17 protein | GH17 |
| 184645_g2 | Glycoside Hydrolase Family 17 protein | GH17 |
| 184338_g2 | Glycoside Hydrolase Family 18 protein | GH18 |
| 184939_g24 | Glycoside Hydrolase Family 18 protein | GH18 |
| 184939_g35 | Glycoside Hydrolase Family 18 protein | GH18 |
| 184845_g46 | Glycoside Hydrolase Family 18 protein | GH18 |
| 184747_g1 | Glycoside Hydrolase Family 18 protein | GH18 |
| 184665_g23 | Glycoside Hydrolase Family 18 protein | GH18 |
| 184941_g24 | Glycoside Hydrolase Family 18 protein | GH18 |
| 184688_g3 | Glycoside Hydrolase Family 18 protein | GH18 |
| 184688_g4 | Glycoside Hydrolase Family 18 protein | GH18 |
| 184992_g8 | Glycoside Hydrolase Family 18 / Carbohydrate-Binding Module Family 5 protein | GH18-CBM5 |
| 184992_g9 | Glycoside Hydrolase Family 18 / Carbohydrate-Binding Module Family 5 protein | GH18-CBM5 |
| 184939_g23 | Glycoside Hydrolase Family 18 / Carbohydrate-Binding Module Family 5 protein | GH18-CBM5 |
| 184615_g9 | Glycoside Hydrolase Family 2 protein | GH2 |
| 184696_g6 | Glycoside Hydrolase Family 2 protein | GH2 |
| 184908_g146 | Glycoside Hydrolase Family 2 protein | GH2 |
| 184867_g5 | Glycoside Hydrolase Family 20 protein | GH20 |
| 184978_g17 | Glycoside Hydrolase Family 20 protein | GH20 |
| 185007_g46 | Glycoside Hydrolase Family 20 protein | GH20 |
| 184970_g37 | Glycoside Hydrolase Family 20 protein | GH20 |
| 184970_g39 | Glycoside Hydrolase Family 20 protein | GH20 |
| 184775_g2 | Glycoside Hydrolase Family 23 protein | GH23 |
| 184970_g4 | Glycoside Hydrolase Family 25 protein | GH25 |
| 184977_g120 | Glycoside Hydrolase Family 27 protein | GH27 |
| 184414_g9 | Glycoside Hydrolase Family 28 protein | GH28 |
| 184579_g19 | Glycoside Hydrolase Family 28 protein | GH28 |
| 184836_g54 | Glycoside Hydrolase Family 28 protein | GH28 |
| 184996_g46 | Glycoside Hydrolase Family 28 protein | GH28 |
| 185041_g7 | Glycoside Hydrolase Family 3 protein | GH3 |
| 184977_g62 | Glycoside Hydrolase Family 3 protein | GH3 |
| 185007_g108 | Glycoside Hydrolase Family 3 protein | GH3 |
| 184868_g9 | Glycoside Hydrolase Family 3 protein | GH3 |
| 184823_g10 | Glycoside Hydrolase Family 3 protein | GH3 |
| 184977_g100 | Glycoside Hydrolase Family 3 protein | GH3 |
| 184817_g14 | Glycoside Hydrolase Family 3 protein | GH3 |
| 184334_g6 | Glycoside Hydrolase Family 30 protein | GH30 |
| 184657_g15 | Glycoside Hydrolase Family 30 protein | GH30_3 |
| 184657_g36 | Glycoside Hydrolase Family 30 protein | GH30_3 |
| 184985_g33 | Glycoside Hydrolase Family 31 protein | GH31 |
| 184798_g2 | Glycoside Hydrolase Family 31 protein | GH31 |
| 184940_g83 | Glycoside Hydrolase Family 31 protein | GH31 |
| 185022_g7 | Glycoside Hydrolase Family 31 protein | GH31 |
| 184603_g6 | Glycoside Hydrolase Family 31 protein | GH31 |
| 184938_g20 | Glycoside Hydrolase Family 32 protein | GH32 |
| 184970_g129 | Glycoside Hydrolase Family 35 protein | GH35 |
| 184970_g147 | Glycoside Hydrolase Family 35 protein | GH35 |
| 184970_g56 | Glycoside Hydrolase Family 37 protein | GH37 |
| 184590_g13 | Glycoside Hydrolase Family 38 protein | GH38 |
| 184884_g55 | Glycoside Hydrolase Family 43 protein | GH43 |
| 184884_g56 | Glycoside Hydrolase Family 43 protein | GH43 |
| 184815_g16 | Glycoside Hydrolase Family 45 protein | GH45 |
| 184815_g42 | Glycoside Hydrolase Family 47 protein | GH47 |
| 184815_g58 | Glycoside Hydrolase Family 47 protein | GH47 |
| 184569_g13 | Glycoside Hydrolase Family 47 protein | GH47 |
| 184763_g4 | Glycoside Hydrolase Family 47 protein | GH47 |
| 185043_g124 | Glycoside Hydrolase Family 47 protein | GH47 |
| 184980_g29 | Glycoside Hydrolase Family 55 protein | GH55 |
| 185002_g87 | Glycoside Hydrolase Family 55 protein | GH55 |
| 184787_g1 | Glycoside Hydrolase Family 5 protein | GH5_12 |
| 184940_g69 | Glycoside Hydrolase Family 5 protein | GH5_12 |
| 184773_g1 | Glycoside Hydrolase Family 5 protein | GH5_12 |
| 184805_g27 | Glycoside Hydrolase Family 5 protein | GH5_15 |
| 184873_g19 | Glycoside Hydrolase Family 5 protein | GH5_22 |
| 184873_g20 | Glycoside Hydrolase Family 5 protein | GH5_22 |
| 185002_g67 | Glycoside Hydrolase Family 5 protein | GH5_50 |
| 184844_g37 | Glycoside Hydrolase Family 5 protein | GH5_50 |
| 185007_g288 | Glycoside Hydrolase Family 5 protein | GH5_7 |
| 184797_g9 | Glycoside Hydrolase Family 5 protein | GH5_9 |
| 185011_g43 | Glycoside Hydrolase Family 5 protein | GH5_9 |
| 185000_g65 | Glycoside Hydrolase Family 5 protein | GH5_9 |
| 185000_g66 | Glycoside Hydrolase Family 5 protein | GH5_9 |
| 184750_g6 | Glycoside Hydrolase Family 63 protein | GH63 |
| 184935_g52 | Glycoside Hydrolase Family 7 protein | GH7 |
| 185007_g75 | Glycoside Hydrolase Family 7 protein | GH7 |
| 184969_g23 | Glycoside Hydrolase Family 7 protein | GH7 |
| 184909_g31 | Glycoside Hydrolase Family 71 protein | GH71 |
| 184909_g32 | Glycoside Hydrolase Family 71 protein | GH71 |
| 184909_g40 | Glycoside Hydrolase Family 71 protein | GH71 |
| 184992_g43 | Glycoside Hydrolase Family 71 protein | GH71 |
| 184942_g35 | Glycoside Hydrolase Family 72 / Carbohydrate-Binding Module Family 43 protein | GH72-CBM43 |
| 184977_g4 | Glycoside Hydrolase Family 74 / Carbohydrate-Binding Module Family 1 protein | GH74-CBM1 |
| 184926_g3 | Glycoside Hydrolase Family 76 protein | GH76 |
| 184926_g4 | Glycoside Hydrolase Family 76 protein | GH76 |
| 184977_g152 | Glycoside Hydrolase Family 78 protein | GH78 |
| 185042_g128 | Glycoside Hydrolase Family 79 protein | GH79 |
| 184902_g33 | Glycoside Hydrolase Family 79 protein | GH79 |
| 184902_g34 | Glycoside Hydrolase Family 79 protein | GH79 |
| 185016_g13 | Glycoside Hydrolase Family 79 protein | GH79 |
| 184594_g3 | Glycoside Hydrolase Family 79 protein | GH79 |
| 184780_g14 | Glycoside Hydrolase Family 79 protein | GH79 |
| 184908_g155 | Glycoside Hydrolase Family 79 protein | GH79 |
| 184902_g35 | Glycoside Hydrolase Family 79 protein | GH79 |
| 184817_g30 | Glycoside Hydrolase Family 79 protein | GH79 |
| 185000_g28 | Glycoside Hydrolase Family 88 protein | GH88 |
| 185000_g29 | Glycoside Hydrolase Family 88 protein | GH88 |
| 185002_g50 | Glycoside Hydrolase Family 89 protein | GH89 |
| 184325_g6 | Glycoside Hydrolase Family 9 protein | GH9 |
| 184969_g71 | Glycoside Hydrolase Family 92 protein | GH92 |
| 184969_g74 | Glycoside Hydrolase Family 92 protein | GH92 |
| 184501_g5 | Glycoside Hydrolase Family 92 protein | GH92 |
| 184817_g11 | Glycoside Hydrolase Family 92 protein | GH92 |
| 184817_g13 | Glycoside Hydrolase Family 92 protein | GH92 |
| 184593_g5 | Glycoside Hydrolase Family 95 protein | GH95 |
| 185042_g149 | Glycosyltransferase Family 1 protein | GT1 |
| 184497_g2 | Glycosyltransferase Family 1 protein | GT1 |
| 184942_g33 | Glycosyltransferase Family 1 protein | GT1 |
| 184787_g19 | Glycosyltransferase Family 1 protein | GT1 |
| 184851_g27 | Glycosyltransferase Family 1 protein | GT1 |
| 184994_g17 | Glycosyltransferase Family 1 protein | GT1 |
| 185042_g148 | Glycosyltransferase Family 1 protein | GT1 |
| 184753_g22 | Glycosyltransferase Family 1 protein | GT1 |
| 184909_g79 | Glycosyltransferase Family 1 protein | GT1 |
| 184863_g22 | Glycosyltransferase Family 15 protein | GT15 |
| 185033_g56 | Glycosyltransferase Family 15 protein | GT15 |
| 184754_g2 | Glycosyltransferase Family 17 protein | GT17 |
| 184494_g3 | Glycosyltransferase Family 2 protein | GT2 |
| 184980_g48 | Glycosyltransferase Family 2 protein | GT2 |
| 185007_g90 | Glycosyltransferase Family 2 protein | GT2 |
| 185043_g239 | Glycosyltransferase Family 2 protein | GT2 |
| 184970_g143 | Glycosyltransferase Family 2 protein | GT2 |
| 184801_g35 | Glycosyltransferase Family 2 protein | GT2 |
| 184908_g149 | Glycosyltransferase Family 2 protein | GT2 |
| 184994_g20 | Glycosyltransferase Family 2 protein | GT2 |
| 184806_g20 | Glycosyltransferase Family 2 protein | GT2 |
| 184941_g31 | Glycosyltransferase Family 2 protein | GT2 |
| 184615_g10 | Glycosyltransferase Family 2 protein | GT2 |
| 184615_g11 | Glycosyltransferase Family 2 protein | GT2 |
| 184757_g8 | Glycosyltransferase Family 20 protein | GT20 |
| 184844_g73 | Glycosyltransferase Family 20 protein | GT20 |
| 184945_g68 | Glycosyltransferase Family 20 protein | GT20 |
| 185007_g36 | Glycosyltransferase Family 21 protein | GT21 |
| 185007_g234 | Glycosyltransferase Family 22 protein | GT22 |
| 185014_g36 | Glycosyltransferase Family 22 protein | GT22 |
| 184939_g28 | Glycosyltransferase Family 22 protein | GT22 |
| 184704_g5 | Glycosyltransferase Family 24 protein | GT24 |
| 184594_g8 | Glycosyltransferase Family 3 protein | GT3 |
| 184911_g76 | Glycosyltransferase Family 31 protein | GT31 |
| 184908_g39 | Glycosyltransferase Family 32 protein | GT32 |
| 185001_g38 | Glycosyltransferase Family 33 protein | GT33 |
| 184633_g5 | Glycosyltransferase Family 35 protein | GT35 |
| 184588_g3 | Glycosyltransferase Family 39 protein | GT39 |
| 184996_g57 | Glycosyltransferase Family 39 protein | GT39 |
| 184938_g56 | Glycosyltransferase Family 39 protein | GT39 |
| 185004_g31 | Glycosyltransferase Family 4 protein | GT4 |
| 184911_g35 | Glycosyltransferase Family 41 protein | GT41 |
| 184969_g87 | Glycosyltransferase Family 48 protein | GT48 |
| 184799_g30 | Glycosyltransferase Family 48 protein | GT48 |
| 184829_g7 | Glycosyltransferase Family 49 protein | GT49 |
| 184909_g11 | Glycosyltransferase Family 57 protein | GT57 |
| 184805_g41 | Glycosyltransferase Family 66 protein | GT66 |
| 184821_g3 | Glycosyltransferase Family 69 protein | GT69 |
| 184857_g7 | Glycosyltransferase Family 69 protein | GT69 |
| 184998_g30 | Glycosyltransferase Family 76 protein | GT76 |
| 185007_g4 | Glycosyltransferase Family 8 protein | GT8 |
| 185043_g240 | Glycosyltransferase Family 8 protein | GT8 |
| 184977_g81 | Glycosyltransferase Family 8 protein | GT8 |
| 184912_g15 | Glycosyltransferase Family 8 protein | GT8 |
| 184636_g14 | Glycosyltransferase Family 8 protein | GT8 |
| 184857_g22 | Glycosyltransferase Family 90 protein | GT90 |
| 184857_g27 | Glycosyltransferase Family 2 | GT2 |
| 184940_g55 | Polysaccharide Lyase Family 14 protein | PL14_4 |
| 184940_g56 | Polysaccharide Lyase Family 14 protein | PL14_4 |
| 184940_g57 | Polysaccharide Lyase Family 14 protein | PL14_4 |
| 185042_g150 | Polysaccharide Lyase Family 14 protein | PL14_5 |
| 184911_g9 | Polysaccharide Lyase Family 4 protein | PL4 |
| 184911_g5 | Polysaccharide Lyase Family 8 protein | PL8_4 |
| 184911_g6 | Polysaccharide Lyase Family 8 protein | PL8_4 |
