# Supplementary material for: The genome of the white-rot fungus Pycnoporus cinnabarinus: a basidiomycete model with a versatile arsenal for lignocellulosic biomass breakdown
Source: BMC Genomics. 2014 Jun 18;15:486. doi: 10.1186/1471-2164-15-486 (PMC4101180; doi:10.1186/1471-2164-15-486)
Supplement: Supplementary file 3 — Additional file 3: Table S3: Annotation of the lignin oxidoreductases predicted from the genome of P. cinnabarinus BRFM137. (DOCX 15 KB) [file 12864_2014_6245_MOESM3_ESM.docx]

**Additional file 3: Table S3.** Annotation of the lignin oxidoreductases predicted from the genome of *P. cinnabarinus* BRFM137.

| ORF number | Predicted enzyme | Detection in the cDNA library and recovery rate compared with predicted cDNA |
| --- | --- | --- |
| scf184857.g29 | AA1, multi-copper oxidase | yes (85-90%) |
| scf185007.g100 | AA1, laccase called Lac3 | yes (98%) |
| scf185007.g107 | AA1, laccase (Lac1 *P. cinnabarinus* BRFM137, Lomascolo et al. 2003**)** | yes (95%) |
| scf184918.g4 | AA1, laccase called Lac4 | very partially |
| scf184817.g29 | AA1, laccase (Lac2 *P. cinnabarinus* BRFM137, Otterbein et al. 2000) | yes (97-98%) |
| scf184845.g66 | AA1, ferroxidase | very partially |
| scf184851.g84 | AA1, laccase called Lac5 | yes (97%) |
| scf184946.g21 | AA2, MnP, VP (called atypical-VP) | yes (95%) |
| scf184473.g24 | AA2, MnP (called MnP2) | no |
| scf184569.g58 | AA2, MnP (called MnP1) | yes (75%) |
| scf184983.g20 | AA2, MnP (called MnP3) | yes (55%) |
| scf184983.g21 | AA2, LiP (called LiP1) | yes (50%) |
| scf184969.g43 | AA2, VP (called VP) | yes (50%) |
| scf184983.g22 | AA2, LiP (called LiP2) | yes (100%) |
| scf184983.g23 | AA2, LiP (called LiP3) | yes (50%) |
| scf184993.g4 | AA2, LiP (called LiP5) | yes (25%) |
| scf184962.g46 | HTP (called HTP1) | yes (50%) |
| scf184962.g49 | HTP (called HTP2) | yes (95%) |
| scf184962.g60 | AA2, LiP (called LiP6) Partial protein | no |
