# Supplementary material for: The genome of the white-rot fungus Pycnoporus cinnabarinus: a basidiomycete model with a versatile arsenal for lignocellulosic biomass breakdown
Source: BMC Genomics. 2014 Jun 18;15:486. doi: 10.1186/1471-2164-15-486 (PMC4101180; doi:10.1186/1471-2164-15-486)
Supplement: Supplementary file 4 — Additional file 4: Table S4: Characteristics of laccase genes from P. cinnabarinus BRFM137. (DOCX 14 KB) [file 12864_2014_6245_MOESM4_ESM.docx]

**Additional file 4: Table S4.** Characteristics of laccase genes from *P.* *cinnabarinus* BRFM137.

|  | Gene length (bp) | ADNc length (b) | Intron number | Exon number |
| --- | --- | --- | --- | --- |
| *lac 1* | 2,131 | 1,557 | 10 | 11 |
| *lac 2* | 2,282 | 1,590 | 12 | 13 |
| *lac 3* | 2,174 | 1,578 | 10 | 11 |
| *lac 4* | 2,128 | 1,578 | 10 | 11 |
| *lac 5* | 2,265 | 1,545 | 12 | 13 |
