# Supplementary material for: The genome of the white-rot fungus Pycnoporus cinnabarinus: a basidiomycete model with a versatile arsenal for lignocellulosic biomass breakdown
Source: BMC Genomics. 2014 Jun 18;15:486. doi: 10.1186/1471-2164-15-486 (PMC4101180; doi:10.1186/1471-2164-15-486)
Supplement: Supplementary file 5 — Additional file 5: Figure S1: Molecular characterization of P. cinnabarinus BRFM137 laccase genes. (DOCX 76 KB) [file 12864_2014_6245_MOESM5_ESM.docx]

*lac5*

*lac1*

*lac2*

*lac3*

*lac4*

0

500

1000

1500

2000

2500 pb

*****

**Additional file 5: Figure S1.** Molecular characterization of *P. cinnabarinus* BRFM137 laccase genes.

Exons Introns
