# Supplementary material for: The genome of the white-rot fungus Pycnoporus cinnabarinus: a basidiomycete model with a versatile arsenal for lignocellulosic biomass breakdown
Source: BMC Genomics. 2014 Jun 18;15:486. doi: 10.1186/1471-2164-15-486 (PMC4101180; doi:10.1186/1471-2164-15-486)
Supplement: Supplementary file 6 — Additional file 6: Data S1: Multiplicity of fungal laccase encoding-genes and gene organization 69 70 71 72 73 74 75 76 77 78 79 80. (DOCX 17 KB) [file 12864_2014_6245_MOESM6_ESM.docx]

**Additional file 6: Data S1. Multiplicity of fungal laccase-encoding genes and gene organization.**

In 1993, Perry *et al*. revealed the presence of two laccase genes located on the same chromosome from the basidiomycete *Agaricus bisporus*. Five distinct laccase genes have been characterized from *Trametes villosa* [69]. These genes were classified into two groups based on their intron/exon structures (*lcc1*/*lcc2* and *lcc3*/*lcc4*/*lcc5*). Similarly, five laccase genes have also been described in *Trametes sanguinea* [70], four in *Rhizoctonia solani* [71], three in *Gaeumannomyces graminis* [72] and three in *Trametes* sp. [73]. Laccase gene families have also been described in the genus *Pleurotus*, where four members were isolated from *P. sajor-caju* [74], two from *P. eryngii* [75] and seven *P. ostreatus* [76]. The progressively increasing availability of fungal genome sequences has led to improved characterization of laccase gene families. Seventeen laccase genes have been identified in the *Coprinopsis cinerea* genome, divided into two sub-groups defined by intron position and percentage similarity of predicted proteins [77]. The first sub-group has 15 members (*lcc1* to *lcc15*) with introns predicted in 16 different positions, with each gene counting between seven and 13 introns. The positional homology of these introns suggested that these genes were derived from duplication events. Furthermore, this first sub-group was itself divided into four groups comprising up to four genes. The second sub-group consisted of only two genes (*lcc16*/*lcc17*) with 18–19 introns. At least nine of these members were translated into functional proteins [74]. Analysis of the genome of the fungus *Laccaria bicolor* also revealed a complex family of multicopper oxidases composed of 11 members, nine of which correspond to laccases *sensu stricto* [78]. A stand-out feature of the *P. cinnabarinus* BRFM137 genome is that in addition to a similar structural organization of several laccase-encoding genes, we were also able to identify their grouping on the same scaffold as for *lac1* and *lac3* genes separated by approximately 23 kb in the same reading frame on scaffold 185007. This physical proximity of *lac* genes has been observed in several genomes. For instance, laccase-encoding genes of *A. bisporus* are organized in tandem and are separated by 1.5 kb [79]. Three genes of the *R. solani* laccases were found on a 12 kb fragment [71]. In *P. ostreatus*, a genomic DNA fragment of 150 kb was found to contain seven laccase genes [76]. In *C. cinerea*, the 17 identified laccase genes are not randomly distributed in the genome but grouped in seven loci in the genome [77]. The multiplicity of genes coding for laccases together with their organization into subgroups could explain the broad range of different physiological functions proposed for this enzyme in the lifecycle of the fungus, such as delignification, formation of fruit bodies, pigment synthesis during asexual development, and pathogenesis [80].
