# Supplementary material for: The genome of the white-rot fungus Pycnoporus cinnabarinus: a basidiomycete model with a versatile arsenal for lignocellulosic biomass breakdown
Source: BMC Genomics. 2014 Jun 18;15:486. doi: 10.1186/1471-2164-15-486 (PMC4101180; doi:10.1186/1471-2164-15-486)
Supplement: Supplementary file 7 — Additional file 7: Table S5: Characteristics of peroxidase genes from P. cinnabarinus BRFM137. (DOCX 14 KB) [file 12864_2014_6245_MOESM7_ESM.docx]

**Additional file 7: Table S5.** Characteristics of peroxidase genes from *P.* *cinnabarinus* BRFM137.

|  | Gene length (bp) | ADNc length (b) | Intron number | Exon number |
| --- | --- | --- | --- | --- |
| ***lip1*** | 1,474 | 1,119 | 6 | 7 |
| ***lip2*** | 1,452 | 1,11 | 6 | 7 |
| ***lip3*** | 1,446 | 1,107 | 6 | 7 |
| ***lip5*** | 1,452 | 1,104 | 6 | 7 |
| ***mnp1*** | 1,396 | 1,095 | 5 | 6 |
| ***mnp2*** | 1,456 | 1,098 | 6 | 7 |
| ***mnp3*** | 1,322 | 1,098 | 4 | 5 |
| ***vp*** | 1,466 | 1,113 | 6 | 7 |
| ***atypical-vp*** | 1,728 | 1,08 | 10 | 11 |
