# Supplementary figures and images for: The genome of the white-rot fungus Pycnoporus cinnabarinus: a basidiomycete model with a versatile arsenal for lignocellulosic biomass breakdown
Source: BMC Genomics. 2014 Jun 18;15:486. doi: 10.1186/1471-2164-15-486 (PMC4101180; doi:10.1186/1471-2164-15-486)

**Additional file 8: Figure S2**


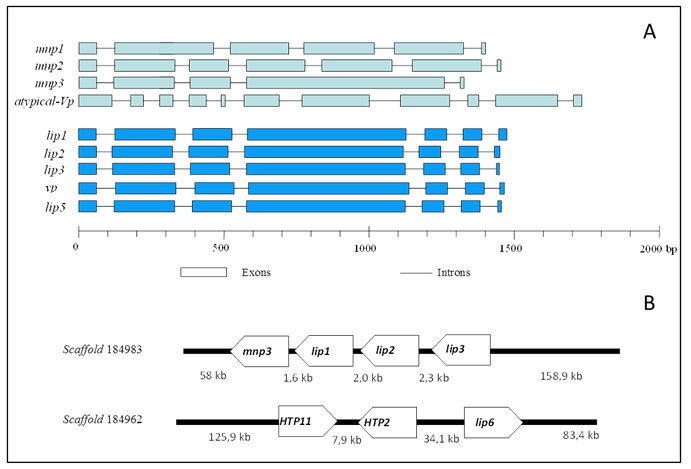

Supplement: Supplementary file 8 — Additional file 8: Figure S2: Molecular characterization of peroxidase genes (A) and schematic organization of peroxidase gene clusters on genomic DNA from P. cinnabarinus BRFM137 (B). (DOCX 64 KB) [file 12864_2014_6245_MOESM8_ESM.docx]

**Additional file 12: Figure S3**


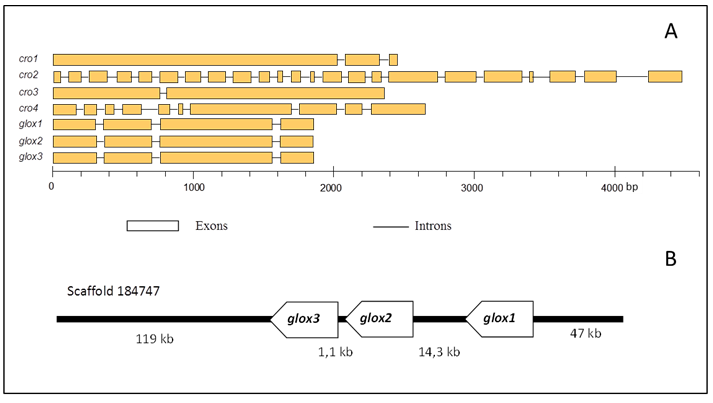

Supplement: Supplementary file 12 — Additional file 12: Figure S3: Molecular characterization of AA5_1 genes (A) and schematic organization of glyoxal oxidase gene clusters on genomic DNA from P. cinnabarinus BRFM137 (B). (DOCX 50 KB) [file 12864_2014_6245_MOESM12_ESM.docx]
