# Supplementary material for: The genome of the white-rot fungus Pycnoporus cinnabarinus: a basidiomycete model with a versatile arsenal for lignocellulosic biomass breakdown
Source: BMC Genomics. 2014 Jun 18;15:486. doi: 10.1186/1471-2164-15-486 (PMC4101180; doi:10.1186/1471-2164-15-486)
Supplement: Supplementary file 9 — Additional file 9: Data S2: Amplification of lip-like genes in white-rot fungi 90 91 92 93. (DOCX 15 KB) [file 12864_2014_6245_MOESM9_ESM.docx]

**Additional file 9: Data S2. Amplification of lip-like genes in white-rot fungi**

Pointing *et al*. [90] were the first to amplify a partial *lip* sequence (500 bp) in the strains *P. sanguineus* CBS614.73 and *P. coccineus* CBS355.63 using degenerated primers. Very recently, in the framework of a phylogenetic analysis, Morgenstern *et al*. [91] sequenced several partial sequences of genes related to peroxidases in *P. cinnabarinus*. However, peroxidase activity has never been clearly demonstrated in the genus *Pycnoporus* in liquid culture. This discrepancy between genomic data and enzyme activity has also been observed in other fungi such as *Phanerochaete sordida* and *Ceriporiopsis subvermipora*, in which the detection of *lip*-like genes and the evidence of a transcript by RT-PCR have never been supported by detectable LiP activity in the culture medium [92]. A study recently confirmed that *C. subvermispora* produces LiP-type enzymes, as demonstrated after heterologous expression and characterization of the corresponding recombinant enzymes [93].
