# Supplementary material for: The genome of the white-rot fungus Pycnoporus cinnabarinus: a basidiomycete model with a versatile arsenal for lignocellulosic biomass breakdown
Source: BMC Genomics. 2014 Jun 18;15:486. doi: 10.1186/1471-2164-15-486 (PMC4101180; doi:10.1186/1471-2164-15-486)
Supplement: Supplementary file 10 — Additional file 10: Table S6: Annotation of the AA5_1 proteins predicted from the genome of P. cinnabarinus BRFM137. (DOCX 14 KB) [file 12864_2014_6245_MOESM10_ESM.docx]

**Additional file 10: Table S6.** Annotation of the AA5_1 proteins predicted from the genome of *P. cinnabarinus* BRFM137.

| ORF number | Predicted enzyme | Detection in the cDNA library and recovery rate compared with predicted cDNA |  |
| --- | --- | --- | --- |
| scf185014.g43 | AA5_1  (called **Cro1**) | yes (100%) | |
| scf185009.g14 | AA5_1 (+**WSC)** (called **Cro2**) | yes (60%) | |
| scf184992.g45 | AA5_1 (called **Cro3**) | yes (90%) | |
| scf184281.g9 | AA5_1 (called **Cro4**) | yes (30%) | |
| scf184747.g41 | AA5_1 (called **Glox 3**) | yes (100%) | |
| scf184747.g42 | AA5_1 (called **Glox2**) | yes (70%) | |
| scf184747.g48 | AA5_1 (called **Glox1**) | yes (70%) | |
