# Supplementary material for: The genome of the white-rot fungus Pycnoporus cinnabarinus: a basidiomycete model with a versatile arsenal for lignocellulosic biomass breakdown
Source: BMC Genomics. 2014 Jun 18;15:486. doi: 10.1186/1471-2164-15-486 (PMC4101180; doi:10.1186/1471-2164-15-486)
Supplement: Supplementary file 11 — Additional file 11: Table S7: Characteristics of AA5_1 genes from P. cinnabarinus BRFM137. (DOCX 14 KB) [file 12864_2014_6245_MOESM11_ESM.docx]

**Additional file 11: Table S7.** Characteristics of AA5_1 genes from *P.* *cinnabarinus* BRFM137.

|  | Gene length (bp) | ADNc length (b) | Intron number | Exon number |
| --- | --- | --- | --- | --- |
| ***cro1*** | 2,448 | 2,328 | 2 | 3 |
| ***cro2*** | 4,451 | 2,993 | 22 | 23 |
| ***cro3*** | 2,360 | 2,313 | 1 | 2 |
| ***cro4*** | 2,634 | 2,052 | 9 | 10 |
| ***glox1*** | 1,854 | 1,671 | 3 | 4 |
| ***glox2*** | 1,850 | 1,680 | 3 | 4 |
| ***glox3*** | 1,856 | 1,677 | 3 | 4 |
