# Supplementary material for: The genome of the white-rot fungus Pycnoporus cinnabarinus: a basidiomycete model with a versatile arsenal for lignocellulosic biomass breakdown
Source: BMC Genomics. 2014 Jun 18;15:486. doi: 10.1186/1471-2164-15-486 (PMC4101180; doi:10.1186/1471-2164-15-486)
Supplement: Supplementary file 13 — Additional file 13: Data S3: Copper radical oxidases of P. chrysosporium [95–97] . (DOCX 16 KB) [file 12864_2014_6245_MOESM13_ESM.docx]

**Additional file 13: Data S3. Copper radical oxidases of *P. chrysosporium***

Preliminary analysis of the *P. chrysosporium* genome found six sequences related to glyoxal oxidases, named *cro1* to *cro6* [95]. Number of introns ranged from three to 23, corresponding to coding sequences ranging from 1947 to 3072 b. However, three genes (*cro3*, *cro4* and *cro5*) appeared to form a more homogeneous group from the perspective of intron/exon organization and position, thus constituting a subgroup of sequences containing a WSC-terminal domain at the *N*-terminus. This type of module has several cysteine residues potentially involved in disulfide bond formation and assumed to be involved in polysaccharide binding [95]. However, their precise role in basidiomycetes remains unclear. In the case of *Pycnoporus*, the *cro2* gene also appeared to possess such a module. The various residues involved in the catalytic site of *P. chrysosporium* Cro were determined using the 3D structure of another fungal copper metalloenzyme, named galactose oxidase (or ‘Gaox’) [96, 97]. These two types of enzymes share low sequence identity in terms of primary sequence but they nevertheless have similar active site structures. Thus, all the Cro identified in *P. chrysosporium* have the conserved residues coordinating the copper atom that constitutes the active site of glyoxal oxidases (Tyr135, Tyr377, His378 and His471). In addition, the cysteine residue Cys70 linked to Tyr135 constituting the redox site appears to have been conserved among the 6 sequences [95].
