# Supplementary material for: The genome of the white-rot fungus Pycnoporus cinnabarinus: a basidiomycete model with a versatile arsenal for lignocellulosic biomass breakdown
Source: BMC Genomics. 2014 Jun 18;15:486. doi: 10.1186/1471-2164-15-486 (PMC4101180; doi:10.1186/1471-2164-15-486)
Supplement: Supplementary file 14 — Additional file 14: Figure S4: Modeling of the active site of the theoretical Glox1 predicted in P. cinnabarinus BRFM137 and comparison with galactose oxidase from Dactylium dendroides. (DOCX 701 KB) [file 12864_2014_6245_MOESM14_ESM.docx]

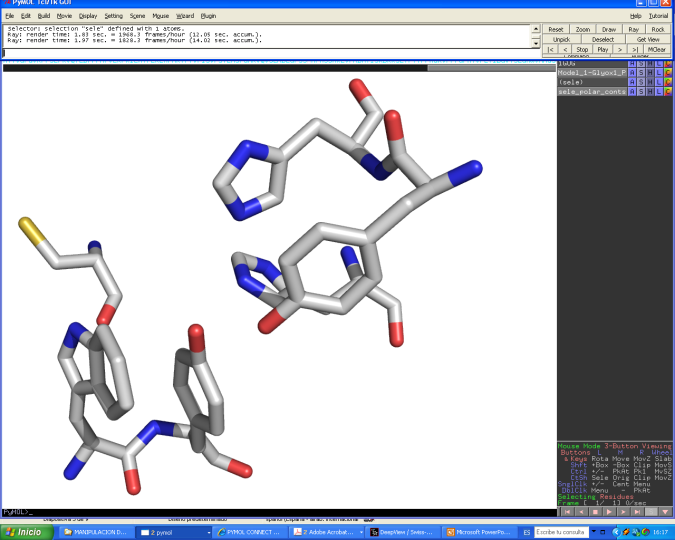

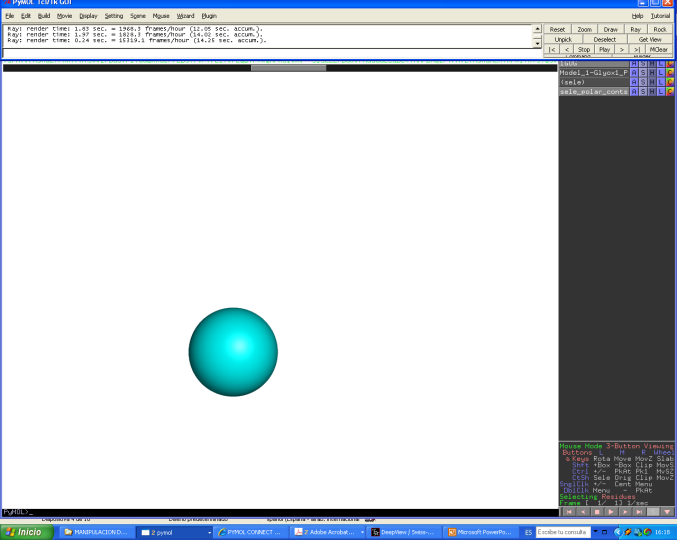


**Cu**

**Cys72**

**Tyr137**

**Trp136**

**Tyr378**

**His379**

**His472**

**Glox1 from *P. cinnabarinus* BRFM137**


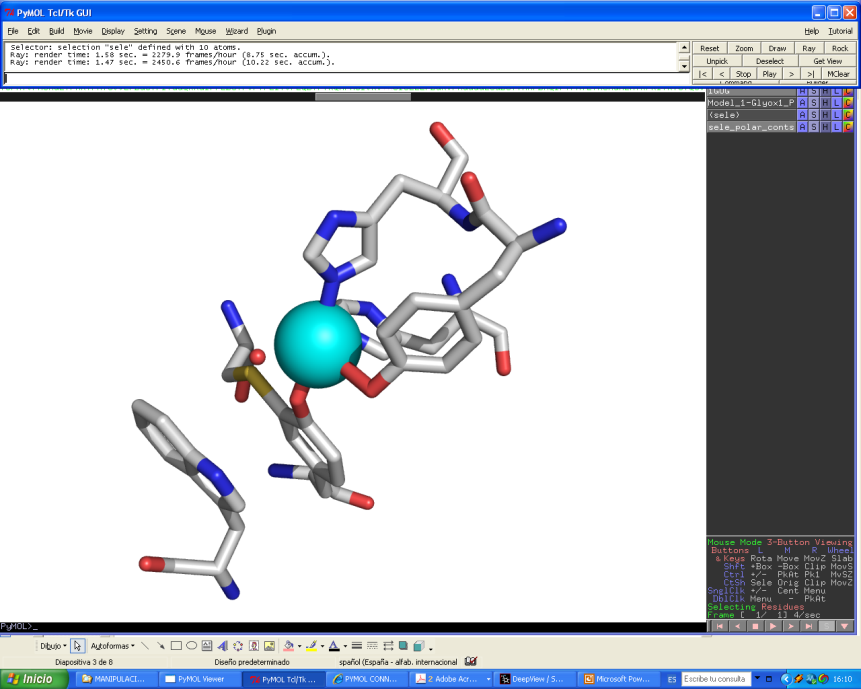


**Cu**

**Cys228**

**Tyr272**

**Trp290**

**Tyr495**

**His496**

**His581**

**Galactose oxidase (PDB: 1GOG)**

**H_2_O**

Tyr-Cys redox cofactor

**Additional file 14: Figure S4**. Modeling of the active site of the theoretical Glox1 predicted in *P. cinnabarinus* BRFM137 and comparison with galactose oxidase from *Dactylium dendroides*.
