# Supplementary material for: The genome of the white-rot fungus Pycnoporus cinnabarinus: a basidiomycete model with a versatile arsenal for lignocellulosic biomass breakdown
Source: BMC Genomics. 2014 Jun 18;15:486. doi: 10.1186/1471-2164-15-486 (PMC4101180; doi:10.1186/1471-2164-15-486)
Supplement: Supplementary file 15 — Additional file 15: Data S4: Structural comparison between Glox1 and Gaox proteins [97]. (DOCX 14 KB) [file 12864_2014_6245_MOESM15_ESM.docx]

**Additional file 15: Data S4. Structural comparison between Glox1 and Gaox proteins.**

T'he alignment of the Glox1 protein sequence from *P. cinnabarinus* with that of Gaox (PDB reference 1GOG) revealed a low percentage identity between the two proteins (21% corresponding to the alignment between Glox1 and three-quarters of the protein sequence from the Gaox C-terminus). Despite this low percentage identity, some of the *P. cinnabarinus* residues involved in the catalytic site of Glox1 are conserved, i.e. the two histidines and two tyrosines involved in the coordination of the copper atom (**Supplementary data, Figure S6**). The main differences between the two proteins were related to: *(i)* the different orientation of the side chain of the Cys72 in *P. cinnabarinus*, which should be directed to the Tyr137 residue compared with its homolog in *Dactylium dendroides*; experimental data [97] suggest a catalytic role for this residue, similar to the Cys228 (covalently linked to Tyr272) in Gaox, *(ii)* the Gaox tryptophan catalytic residue (Trp290, which plays multiple critical roles in enzymatic catalysis), which seemed to be replaced by the Trp136 residue, close to the Tyr137 residue in the 3D model structure proposed.
