# Supplementary material for: The genome of the white-rot fungus Pycnoporus cinnabarinus: a basidiomycete model with a versatile arsenal for lignocellulosic biomass breakdown
Source: BMC Genomics. 2014 Jun 18;15:486. doi: 10.1186/1471-2164-15-486 (PMC4101180; doi:10.1186/1471-2164-15-486)
Supplement: Supplementary file 17 — Additional file 17: Table S9: List of unique and common genes identified in the different growth conditions. (DOCX 18 KB) [file 12864_2014_6245_MOESM17_ESM.docx]

**Additional file 17: Table S9. List of unique and common genes identified in the different growth conditions.**

**LC-M:** Liquid cultures with 20 g/l maltose

**LC-B:** Liquid cultures with 5 g/l maltose and 15 g/l micronized birchwood

**LC-M-MB-A:** Liquid cultures with 5 g/l maltose, 15 g/l Avicel cellulose (Sigma) and 15 g/l autoclaved maize bran

**SSF**: Solid-state fermentation cultures with: sugarcane bagasse, banana skins, wood shavings, hemp and micronized birchwood

**Elements only in LC M:**

scf184946_g21 AA2

scf184938_g40 CE16

scf184916_g7 CE16

scf185043_g112 GH125

scf185007_g46 GH20

scf184836_g54 GH28

scf184938_g20 GH32

scf184970_g147 GH35

scf185002_g87 GH55

scf184977_g152 GH78

scf184594_g3 GH79

scf184911_g6 PL8_4

**Elements only in LC B:**

scf184992_g18 AA6

scf184831_g7 AA9

scf185037_g14 AA9

scf184747_g17 AA9-CBM1

scf184616_g3 CBM1

scf184727_g5 CBM1-GH5_5

scf184921_g29 CBM1-GH5_5

scf184977_g63 GH131

scf184911_g15 GH16

scf184980_g23 GH17

scf184977_g62 GH3

scf185000_g65 GH5_9

scf184817_g11 GH92

**Elements only in LC M-MB-A:**

scf184844_g129 AA9

scf184969_g36 CBM1-CE1

scf184856_g2 CBM1-CE15

scf184817_g18 CBM1-GH10

scf184727_g5 CBM1-GH5_5

scf184866_g20 GH10

scf184866_g23 GH10

scf184817_g14 GH3

scf184748_g21 GH51

scf184969_g74 GH92

**Elements only in SSF:**

scf184983_g23 AA2

scf184569_g58 AA2

scf184866_g23 CBM1-GH10

scf185013_g116 CE8

scf184449_g5 GH10

scf184712_g6 GH13

scf184992_g11 GH13-CBM20

scf184902_g23 GH16

scf184983_g60 GH16

scf184989_g27 GH16

scf184992_g8 GH18-CBM5

scf184798_g90 GH43

scf185000_g65 GH5

scf185000_g66 GH5

scf185002_g86 GH5

scf184501_g5 GH92

scf185042_g150 PL14

**Common elements in LC M,** **LC B, LC M-MB-A and SSF:**

scf185007_g107 AA1_1

scf185007_g100 AA1_1

scf184817_g29 AA1_1

scf184746_g13 AA3_2

scf184803_g17 AA3_2

scf184992_g45 AA5_1

scf185014_g56 CBM1-GH6

scf184942_g36 CE4

scf184806_g33 GH12

scf184338_g2 GH18

scf184747_g1 GH18

scf184970_g4 GH25

scf184977_g120 GH27

scf184414_g9 GH28

scf184996_g46 GH28

scf184868_g9 GH3

scf184334_g6 GH30

scf185022_g7 GH31

scf184970_g129 GH35

scf184569_g13 GH47

scf184980_g29 GH55

scf185007_g75 GH7

scf184969_g23 GH7

scf184942_g35 GH72-CBM43

scf184926_g3 GH76

scf184926_g4 GH76

scf184969_g71 GH92

scf184593_g5 GH95

scf184911_g5 PL8_4

**Common elements in LC M and LC B:**

scf185002_g8 AA3_2

scf185013_g1 AA8-AA3_1

scf184712_g7 CBM13

scf184654_g6 CBM1-GH131

scf185007_g51 CBM1-GH5_5

scf184961_g16 CBM1-GH5_7

scf184751_g9 CE16

scf185042_g12 GH10

scf184712_g6 GH13_1

scf184992_g11 GH13_32-CBM20

scf184935_g11 GH16

scf184645_g2 GH17

scf184657_g36 GH30_3

scf184970_g56 GH37

scf185000_g66 GH5_9

scf185042_g150 PL14_5

**Common elements in LC B and LC M-MB-A:**

none

**Common elements in LC M-MB-A and SSF:**

scf185013_g1 AA3_1

scf184908_g125 AA9

scf184654_g6 CBM1

scf184830_g3 CBM18-GH16

scf184961_g16 CBM1-GH5_7

scf185007_g51 CBM1-GH5_5

scf184866_g22 GH10

scf184970_g119 GH12

scf184622_g5 GH15

scf184657_g36 GH30

scf184985_g3 GH31

**Common elements in LC M,** **LC B and LC M-MB-A:**

scf184611_g7 AA3_2

scf185009_g14 AA5_1

scf184939_g35 GH18

scf184615_g9 GH2

scf184603_g6 GH31

scf184817_g30 GH79

scf185000_g28 GH88

scf185000_g29 GH88

**Common elements in LC B,** **LC M-MB-A and SSF:**

scf184817_g21 AA9

scf184980_g44 CBM1-CE1

scf184977_g100 GH3

scf184935_g52 GH7

scf184977_g4 GH74-CBM1

**Common elements in LC M** **and LC M-MB-A:**

scf184913_g15 AA3_4

scf184747_g42 AA5_1

scf184940_g83 GH31

**Common elements in LC B** **and SSF:**

scf185015_g16 GH16

scf184801_g33 GH16

scf184845_g46 GH18

**Common elements in LC M,** **LC M-MB-A and SSF:**

scf184913_g15 AA3_4

scf184747_g42 AA5_1

scf184940_g83 GH31

**Common elements in LC M,** **LC B and SSF:**

scf185015_g65 GH16

scf184392_g3 GH16

scf184688_g3 GH18

scf184688_g4 GH18

**Common elements in LC M** **and SSF:**

scf184829_g10 GH16

scf184939_g23 GH18-CBM5

scf184970_g39 GH20
