# Supplementary material for: The genome of the white-rot fungus Pycnoporus cinnabarinus: a basidiomycete model with a versatile arsenal for lignocellulosic biomass breakdown
Source: BMC Genomics. 2014 Jun 18;15:486. doi: 10.1186/1471-2164-15-486 (PMC4101180; doi:10.1186/1471-2164-15-486)
Supplement: Supplementary file 18 — Additional file 18: Table S10: P. cinnabarinus genome annotation related to protein secretion pathways. (DOCX 48 KB) [file 12864_2014_6245_MOESM18_ESM.docx]

**Additional file 18: Table S10. *P. cinnabarinus* genome annotation related to protein secretion pathways**

| **ENTRY** **INTO** **ER** | | | | |
| --- | --- | --- | --- | --- |
| **SIGNAL** **RECOGNITION** | | | | |
| *Pycnoporus cinnereus* Gene ID | *Aspergillus niger* Gene ID | *Saccharomyces cerevisiae* Gene ID | Gene Name | Description in SGD |
| No hits found | An01g02800 | YPL243w | SRP68 | Core component of the signal recognition particle (SRP) ribonucleoprotein (RNP) complex that functions in targeting nascent secretory proteins to the endoplasmic reticulum (ER) membrane |
| No hits found | An04g06890 | YPL210c | SRP72 | Core component of the signal recognition particle (SRP) ribonucleoprotein (RNP) complex that functions in targeting nascent secretory proteins to the endoplasmic reticulum (ER) membrane |
| scf184845.g53 | An01g10070 | YML105c | SEC65 | Subunit of the signal recognition particle (SRP), involved in protein targeting to the ER; interacts with Srp54p; homolog of mammalian SRP19 |
| scf184637.g5 | An15g06470* |  | - | similarity to signal sequence receptor alpha chain Canis lupus |
| No hits found | An07g05800 | YDL092w | SRP14 | Signal recognition particle (SRP) subunit |
| scf184785.g11 | An09g06320 | YPR088c | SRP54 | Signal recognition particle (SRP) subunit (homolog of mammalian SRP54) |
| scf184798.g87 | An15g01670 | YDR292c | SRP101 | Signal recognition particle (SRP) receptor alpha subunit; contain GTPase domains; involved in SRP-dependent protein targeting; interacts with the beta subunit, Srp102p |
| No hits found | An05g00140 | YKL154w | SRP102 | Signal recognition particle (SRP) receptor beta subunit; involved in SRP-dependent protein targeting; anchors the alpha subunit, Srp101p to the ER membrane |
|  |  |  |  |  |
| **SIGNAL** **PEPTIDASE COMPLEX** | | | | |
| scf184996.g50 | An01g00560 | YIR022w | SEC11 | subunit of the Signal Peptidase Complex which cleaves the signal sequence of proteins targeted to the ER |
| No hits found | An17g02095 | YJR010c-a | SPC1 | subunit of the Signal Peptidase Complex which cleaves the signal sequence of proteins targeted to the ER |
| scf184600.g16 | An16g07390* | YML055w | SPC2 | subunit of the Signal Peptidase Complex which cleaves the signal sequence of proteins targeted to the ER |
| scf184696.g16 | An09g05420* | YLR066w | SPC3 | subunit of the Signal Peptidase Complex which cleaves the signal sequence of proteins targeted to the ER |
|  |  |  |  |  |
| **TRANSLOCATION** **INTO** **ER** | | | | |
| scf184707.g3  scf184707.g1*^2^  scf184874.g3*^2^  scf184711.g1*^2^  scf184492.g1*^2^ | An03g04340 | YLR378c | SEC61 | subunit of Sec61 complex (Sec61p, Sbh1p, and Sss1p); forms a channel for SRP-dependent protein import and retrograde transport of misfolded proteins out of the ER; |
| No hits found | An01g03820 | YER087c-b | SBH1 | Beta subunit of the Sec61p ER translocation complex (Sec61p-Sss1p-Sbh1p); involved in protein translocation into the endoplasmic reticulum |
| scf185007.g14 | An01g11630 | YDR086c | SSS1 | Beta subunit of the Sec61p ER translocation complex (Sec61p-Sss1p-Sbh1p); involved in protein translocation into the endoplasmic reticulum |
| No hits found | An01g03820 | YER019c-a | SBH2 | Ssh1p-Sss1p-Sbh2p complex component, involved in protein translocation into the endoplasmic reticulum |
| scf184583.g3 | An02g01510 | YPL094c | SEC62 | Essential subunit of Sec63 complex (Sec63p, Sec62p, Sec66p and Sec72p); with Sec61 complex, Kar2p/BiP and Lhs1p forms a channel competent for SRP-dependent and post-translational SRP-independent protein targeting and import into the ER |
| scf184370.g7 | An01g13070 | YOR254c | SEC63 | Essential subunit of Sec63 complex (Sec63p, Sec62p, Sec66p and Sec72p); with Sec61 complex, Kar2p/BiP and Lhs1p forms a channel competent for SRP-dependent and post-translational SRP-independent protein targeting and import into the ER |
| scf184863.g26 | An16g08830 | YBR171w | SEC66 | Non-essential subunit of Sec63 complex (Sec63p, Sec62p, Sec66p and Sec72p); with Sec61 complex, Kar2p/BiP and Lhs1p forms a channel competent for SRP-dependent and post-translational SRP-independent protein targeting and import into the ER |
|  |  |  |  |  |
| **PROTEIN** **FOLDING IN THE ER** | | | | |
| scf184858.g22 | An02g14800 | YCL043c | PDI1 | Protein disulfide isomerase; essential for the formation of disulfide bonds in secretory and cell-surface proteins, unscrambles non-native disulfide bonds |
| scf184844.g23 | An18g02020 | YCL043c | PDI1 | Protein disulfide isomerase; essential for the formation of disulfide bonds in secretory and cell-surface proteins, unscrambles non-native disulfide bonds |
| scf184844.g23*  scf184858.g22* | An01g04600 | YOR288C | MPD1 | Member of the protein disulfide isomerase (PDI) family |
| scf184613.g2 | An02g05890 | YIL005W | EPS1 | ER protein with chaperone and co-chaperone activity, involved in retention of resident ER proteins; has a role in recognizing proteins targeted for ER-associated degradation (ERAD), member of the protein disulfide isomerase family |
| scf184775.g6 | An16g07620 | YML130c | ERO1 | Thiol oxidase required for oxidative protein folding in the endoplasmic reticulum |
| scf184395.g1 | An08g07810 | YDL045c | FAD1 | Flavin adenine dinucleotide (FAD) synthetase, performs the second step in synthesis of FAD from riboflavin |
| scf185015.g72 | An08g06370 | YBL033c | RIB1 | GTP cyclohydrolase II; catalyzes the first step of the riboflavin biosynthesis p |
| scf184900.g4 | An10g00350 | - | - | similarity to GTP cyclohydrolase II ribA - Actinobacillus  pleuropneumoniae |
| scf184794.g18 | An12g06490 | - | - | similarity to N-oxide-forming dimethylaniline monooxygenase  FMO1 - Homo sapiens |
| No hits found | An18g06470 | - | - | similarity to N-oxide-forming dimethylaniline monooxygenase  FMO1 - Homo sapiens |
| scf184797.g7 | An05g00880 | YMR214w | SCJ1 | homolog of bacterial chaperone DnaJ, located in the ER lumen where it cooperates with Kar2p to mediate maturation of proteins |
| scf185007.g186 | An01g08420 | YAL058w | CNE1 | Calnexin; integral membrane ER chaperone involved in folding and quality control of glycoproteins |
| scf184966.g13 | An04g02020 | YDR155c | CPR1 | Cytoplasmic peptidyl-prolyl cis-trans isomerase (cyclophilin), catalyzes the cis-trans isomerization of peptide bonds N-terminal to proline residues |
| scf184914.g1 | An01g06670 | YDR519w | FPR2 | Membrane-bound peptidyl-prolyl cis-trans isomerase (PPIase |
| scf184999.g48 | An11g04180 | YJL034w | KAR2 | ATPase involved in protein import into the ER, also acts as a chaperone to mediate protein folding in the ER and may play a role in ER export of soluble proteins; regulates the unfolded protein response via interaction with Ire1p |
|  |  |  |  |  |
| scf184902.g6 | An01g13220 | YJL034w | KAR2 | ATPase involved in protein import into the ER, also acts as a chaperone to mediate protein folding in the ER and may play a role in ER export of soluble proteins; regulates the unfolded protein response via interaction with Ire1p |
|  |  |  |  |  |
| **PROTEIN** **MISFOLDING** | | | | |
| **UNFOLDED** **PROTEIN** **RESPONSE** **(UPR)** | | | | |
| No hits found | An01g00160 | YFL031W | HAC1 | Basic leucine zipper (bZIP) transcription factor (ATF/CREB1 homolog) that regulates the unfolded protein response |
| scf184776.g5 | An01g06550 | YHR079c | IRE1 | Serine-threonine kinase and endoribonuclease; transmembrane protein that mediates the UPR by regulating Hac1p synthesis through HAC1 mRNA splicing |
| scf184855.g11 scf184961.g15 | An14g04770 | YDL006w | PTC1 | Type 2C protein phosphatase (PP2C) |
| scf184909.g43 | An08g00830 | YER089c | PTC2 | Type 2C protein phosphatase (PP2C); dephosphorylates Ire1p to downregulate the unfolded protein response |
| scf184926.g8 | An08g01480 | YJL087c | TRL1 | tRNA ligase, required for tRNA splicing and for both splicing and translation of HAC1 mRNA in the UPR |
| No hits found | An01g07900 | YEL009c | GCN4 | bZIP transcriptional activator of amino acid biosynthetic genes in response to amino acid starvation; expression is tightly regulated at both the transcriptional and translational |
| scf184817.g21 | An11g11250 | YLR090w | XDJ1 | Putative chaperone, homolog of E. coli DnaJ |
| scf184759.g9 | An01g08980 | YGR038w | ORM1 | required for resistance to agents that induce unfolded protein response; |
|  |  |  |  |  |
| **ER ASSOCIATED DEGRADATION (ERAD)** | | | | |
| scf184805.g32 scf184970.g113 scf184908.g154 | An04g09170 | YDL126c | CDC48 | ATPase involved in ubiquitin-mediated protein degradation |
| scf184990.g26 | An01g05330 | YBR170c | NPL4 | Ubiquitin-binding protein involved in protein degradation; Cdc48p-Npl4p-Ufd1p complex participates in (ERAD) |
| scf185002.g115 | An01g05760 | YGR048w | UFD1 | Involved in recognition of polyubiquitinated proteins and their presentation to the 26S proteasome for degradation |
| scf184909.g16  scf184693.g11 | An15g00640 | YBR201W | DER1 | ER membrane protein, required for ERAD of misfolded or unassembled proteins; |
| scf185014.g85 | An03g04600 | YKL213c | DOA1 | WD repeat protein required for ubiquitin-mediated protein degradation |
| scf184499.g21 | An08g09000 | YMR276w | DSK2 | Nuclear-enriched ubiquitin-like polyubiquitin-binding protein |
| scf184677.g8 | An16g07970 | YOL013c | HRD1 | Ubiquitin-protein ligase; required for ERAD of misfolded proteins |
| scf184970.g123 | An17g00260 | YDR177w | UBC1 | Ubiquitin-conjugating enzyme that mediates selective degradation of short-lived and abnormal proteins |
| scf184767.g2 | An06g01120 | YGL058w | RAD6 | Ubiquitin-conjugating enzyme (E2), |
| scf184999.g39 | An09g06110 | YMR022w | UBC7 | Ubiquitin conjugating enzyme, involved in the ER-associated protein degradation pathway; |
| scf184909.g21 | An04g01730 | YDL190c | UFD2 | Ubiquitin chain assembly factor (E4) that cooperates with a ubiquitin-activating enzyme (E1), a ubiquitin-conjugating enzyme (E2), and a ubiquitin protein ligase (E3) to conjugate ubiquitin to substrates |
| scf184851.g10 | An04g01720 | YMR161w | HLJ1 | Co-chaperone for Hsp40p, anchored in the ER membrane; with its homolog Ydj1p promotes ER-associated protein degradation (ERAD) of integral membrane substrates |
| scf185043.g124 | An12g00340 | YHR204w | MNL1 | Alpha-1,2-specific exomannosidase of the endoplasmic reticulum; |
| scf184763.g4 | An18g06220 | YJR131w | MNS1 | Alpha-1,2-mannosidase involved in ER-associated protein degradation (ERAD |
| scf184650.g6  scf184981.g1 | An04g00360 | YLR208w | SEC13 | Structural component of three distinct complexes |
| scf184917.g17 | An12g04000 | YIL030c | SSM4 | Ubiquitin-protein ligase involved in ER-associated protein degradation |
| scf184962.g12 | An14g00230 | YER100w | UBC6 | Ubiquitin-conjugating enzyme involved in ER-associated protein degradation |
| scf184636.g10 | An01g04280 | YNL064c | YDJ1 | Type I HSP40 co-chaperone involved in regulation of the HSP90 and HSP70 functions; involved in protein translocation across membranes |
| scf184750.g6 | An15g01420 | YGL027c | CWH41 | Processing alpha glucosidase I, ER type II integral membrane N-glycoprotein involved in assembly of cell wall beta 1,6 glucan and asparagine-linked protein glycosylation; also involved in ER protein quality control and sensing of ER stress |
|  |  |  |  |  |
| **PROTEASOME** | | | | |
| No hits found with | An18g06520 | YHL030w | ECM29 | Scaffold protein that assists in association of the proteasome core particle with the regulatory particle |
| scf184280.g10 | An18g03010 | YHR027c | RPN1 | Non-ATPase base subunit of the 19S regulatory particle of the 26S proteasome; |
| scf185013.g137 | An04g03270* | YIL075c | RPN2 | Subunit of the 26S proteasome, substrate of the N-acetyltransferase Nat1p |
| scf184569.g18 | An11g10380 | YER021w | RPN3 | Essential, non-ATPase regulatory subunit of the 26S proteasome lid |
| No hits found | An08g06850 | YDL020c | RPN4 | Transcription factor that stimulates expression of proteasome genes; |
| scf185013.g98 | An11g09690 | YDL147w | RPN5 | Subunit of the COP9 signalosome (CSN) and non-ATPase regulatory subunit of the 26S proteasome |
| scf184895.g14  scf184702.g6  scf184895.g15 | An18g05070 | YDL097c | RPN6 | Essential, non-ATPase regulatory subunit of the 26S proteasome |
| scf184895.g13 | An11g02610 | YPR108w | RPN7 | Essential, non-ATPase regulatory subunit of the 26S proteasome, similar |
| scf184996.g61 | An07g10110 | YOR261c | RPN8 | Essential, non-ATPase regulatory subunit of the 26S proteasome |
| scf184788.g16 | An08g10710 | YDR427w | RPN9 | Non-ATPase regulatory subunit of the 26S proteasome |
| scf184613.g5 | An15g03020 | YHR200w | RPN10 | Non-ATPase base subunit of the 19S regulatory particle (RP) of the 26S proteasome |
| scf184908.g81 | An07g07860 | YFR004w | RPN11 | Metalloprotease subunit of the 19S regulatory particle of the 26S proteasome lid |
| scf184652.g4 | An16g02210 | YFR052w | RPN12 | Subunit of the 19S regulatory particle of the 26S proteasome lid; |
| scf184593.g7 | An02g12760 | YKL145w | RPT1 | One of six ATPases of the 19S regulatory particle of the 26S proteasome |
| scf184756.g27 | An17g00270 | YDL007w | RPT2 | One of six ATPases of the 19S regulatory particle of the 26S proteasome |
| scf184785.g44 | An02g07190 | YDR394w | RPT3 | One of six ATPases of the 19S regulatory particle of the 26S proteasome |
| scf184815.g19 | An18g06230 | YOR259c | RPT4 | One of six ATPases of the 19S regulatory particle of the 26S proteasome |
| scf184915.g60 | An18g05230 | YOR117w | RPT5 | One of six ATPases of the 19S regulatory particle of the 26S proteasome |
| scf184845.g22 | An14g00180 | YGL048c | RPT6 | One of six ATPases of the 19S regulatory particle of the 26S proteasome |
|  |  |  |  |  |

*^2^ Partial ORF identical to first hit

| **Protein complex involved in protein transport** | | | | |
| --- | --- | --- | --- | --- |
| **Exocyst Complex** | | | | |
| scf185007.g35 | An01g03190 | YER008c | SEC3 | Subunit of the exocyst complex which mediates targeting of post-Golgi vesicles to sites of active exocytosis |
| scf184921.g16 | An08g05570 | YDR166c | SEC5 | Subunit of the exocyst complex which mediates targeting of post-Golgi vesicles to sites of active exocytosis |
| scf184962.g24 | An04g06180 | YIL068c | SEC6 | Subunit of the exocyst complex which mediates targeting of post-Golgi vesicles to sites of active exocytosis |
| scf185007.g49 | An03g04210 | YPR055w | SEC8 | Subunit of the exocyst complex which mediates targeting of post-Golgi vesicles to sites of active exocytosis |
| scf185003.g18 | An03g06900 | YLR166c | SEC10 | Subunit of the exocyst complex which mediates targeting of post-Golgi vesicles to sites of active exocytosis |
| scf184908.g161 | An15g00010 | YGL233w | SEC15 | Subunit of the exocyst complex which mediates targeting of post-Golgi vesicles to sites of active exocytosis |
| scf184926.g7 | An02g04030* | YJL085w | EXO70 | Subunit of the exocyst complex which mediates targeting of post-Golgi vesicles to sites of active exocytosis |
| scf184753.g42 | An08g07370 | YBR102c | EXO84 | Subunit of the exocyst complex which mediates targeting of post-Golgi vesicles to sites of active exocytosis |
| scf185001.g27 | An14g00010 | YFL005w | SAR1 | Subunit of the exocyst complex which mediates targeting of post-Golgi vesicles to sites of active exocytosis |
| scf184981.g2 | An18g05980 | YPR165w | RHO1 | Subunit of the exocyst complex which mediates targeting of post-Golgi vesicles to sites of active exocytosis |
| scf184981.g2* | An16g04200 | YNL090w | RHO2 | Non-essential small GTPase of the Rho/Rac subfamily involved in the establishment of cell polarity and in microtubule assembly |
| scf185008.g5 | An11g09620 | YIL118w | RHO3 | Non-essential small GTPase of the Rho/Rac subfamily of Ras-like proteins involved in the establishment of cell polarity |
| scf184636.g11 | An14g05530 | YKR055w | RHO4 | Non-essential small GTPase of the Rho/Rac subfamily of Ras-like proteins, likely to be involved in the establishment of cell polarity |
| scf185007.g269 | An11g10030 | YNL180c | RHO5/RAC | Non-essential small GTPase of the Rho/Rac subfamily of Ras-like proteins, likely involved in protein kinase C (Pkc1p)-dependent signal transduction pathway that controls cell integrity |
| scf185000.g58 | An02g14200 | YLR229c | CDC42 | Small rho-like GTPase, essential for establishment and maintenance of cell polarity; mutants have defects in the organization of actin and septins |
| + |  |  |  |  |
| **SEC34/SEC35 Complex** | | | | |
| scf184585.g3 | An02g06840 | YER157w | SEC34 | Essential component of the conserved oligomeric Golgi complex (Cog1p through Cog8p), a cytosolic tethering complex that functions in protein trafficking to mediate fusion of transport vesicles to Golgi compartments |
| scf184834.g50 | An02g14400 | YPR105c | COG4 | Essential component of the conserved oligomeric Golgi complex (Cog1p through Cog8p), a cytosolic tethering complex that functions in protein trafficking to mediate fusion of transport vesicles to Golgi compartments |
| No hits | An16g03450 | YNL051w | COG5 | Component of the conserved oligomeric Golgi complex (Cog1p through Cog8p), a cytosolic tethering complex that functions in protein trafficking to mediate fusion of transport vesicles to Golgi compartments ([1](http://www.yeastgenome.org/cgi-bin/locus.fpl?locus=YNL051w#S000069911), [3](http://www.yeastgenome.org/cgi-bin/locus.fpl?locus=YNL051w#S000076590)) |
| scf185014.g111 | An06g01630 | YNL041c | COG6/SEC37 | Component of the conserved oligomeric Golgi complex (Cog1p through Cog8p), a cytosolic tethering complex that functions in protein trafficking to mediate fusion of transport vesicles to Golgi compartments |
|  |  |  |  |  |
| **Trapp Complex** | | | | |
| scf184799.g53 | An12g00380 | YKR068c | BET3 | Hydrophilic protein that acts in conjunction with SNARE proteins in targeting and fusion of ER to Golgi transport vesicles; component of the TRAPP |
| scf184979.g5 | An17g01875 | YML077w | BET5 | Component of the TRAPP (transport protein particle) complex, which plays an essential role in the vesicular transport from endoplasmic reticulum to Golgi |
| No hits found | An04g08690 | YDR108w | TRS85 | Subunit of TRAPPIII (transport protein particle), a multimeric guanine nucleotide-exchange factor for Ypt1p, required for membrane expansion during autophagy and the CVT pathway; directs Ypt1p to the PAS; late post-replication meiotic role |
| scf184863.g20 | An15g00470 | YDR407c | TRS120 | One of 10 subunits of the transport protein particle (TRAPP) complex of the cis-Golgi which mediates vesicle docking and fusion; involved in endoplasmic reticulum (ER) to Golgi membrane traffic |
| scf185002.g59 | An08g05190 | YMR218c | TRS130 | One of 10 subunits of the transport protein particle (TRAPP) complex of the cis-Golgi which mediates vesicle docking and fusion; involved in ER to Golgi membrane traffic; mutation activates transcription of OCH1 |
| No hits found | An15g03010 | YDR246w | TRS23 | One of 10 subunits of the transport protein particle (TRAPP) complex of the cis-Golgi which mediates vesicle docking and fusion; involved in endoplasmic reticulum (ER) to Golgi membrane traffic; human homolog is TRAPPC4 |
| scf184829.g45 | An14g06440 | YDR472w | TRS31 | One of 10 subunits of the transport protein particle (TRAPP) complex of the cis-Golgi which mediates vesicle docking and fusion; involved in endoplasmic reticulum (ER) to Golgi membrane traffic |
| scf184829.g75 | An15g00060 | YOR115c | TRS33 | One of 10 subunits of the transport protein particle (TRAPP) complex of the cis-Golgi which mediates vesicle docking and fusion; involved in endoplasmic reticulum (ER) to Golgi membrane traffic |
|  |  |  |  |  |
| **COPI SUBUNITS** | | | | |
| scf184970.g118 | An16g05370 | YER122c | GLO3 | ADP-ribosylation factor GTPase activating protein (ARF GAP), involved in ER-Golgi transport; |
| scf184569.g24 | An16g02460 | YDL145c | COP1 | Alpha subunit of COPI vesicle coatomer complex, which surrounds transport vesicles in the early secretory pathway |
| scf185013.g48 | An01g14260 | YFR051c | RET2 | Delta subunit of the coatomer complex (COPI), which coats Golgi-derived transport vesicles; involved in retrograde transport between Golgi and ER |
| scf184847.g10 | An12g04830 | YPL010w | RET3 | Zeta subunit of the coatomer complex (COPI), which coats Golgi-derived transport vesicles; involved in retrograde transport between Golgi and ER |
| scf185007.g92 | An07g06030  An14g03050 | YNL287w | SEC21 | Gamma subunit of coatomer, a heptameric protein complex that together with Arf1p forms the COPI coat; involved in ER to Golgi transport of selective cargo |
| scf184494.g2 | An02g05870 | YGL137w | SEC27 | Essential beta'-coat protein of the COPI coatomer, involved in ER-to-Golgi and Golgi-to-ER transport; contains WD40 domains that mediate cargo selective interactions; |
| scf184908.g162 | An08g03690 | YDL137w | ARF1/ARF2 | ADP-ribosylation factor, GTPase of the Ras superfamily involved in regulation of coated formation vesicles in intracellular trafficking within the Golgi; |
|  |  |  |  |  |
| **COPII SUBUNITS** | | | | |
| scf185002.g129 | An01g04040 | YPL218w | SAR1 | GTPase, GTP-binding protein of the ARF family, component of COPII coat of vesicles; required for transport vesicle formation during ER to Golgi protein transport |
| scf185007.g231 | An08g03270 | YDR238c | SEC26 | Essential beta-coat protein of the COPI coatomer, involved in ER-to-Golgi protein trafficking and maintenance of normal ER morphology |
| scf184650.g6 | An04g00360 | YLR208w | SEC13 | Structural component of three distinct complexes; subunit of the Nup84 nuclear pore sub-complex (NPC), the COPII vesicle coat, and the Seh1-associated (SEA) complex |
| scf185002.g1 | An02g01690 | YDL195w | SEC31 | ponent of the Sec13p-Sec31p complex of the COPII vesicle coat, required for vesicle formation in ER to Golgi transport |
| scf184844.g77 | An01g04730 | YPR181c | SEC23 | GTPase-activating protein, stimulates the GTPase activity of Sar1p; component of the Sec23p-Sec24p heterodimer of the COPII vesicle coat, involved in ER to Golgi transport |
| scf184790.g19 | An08g10650 | YIL109c | SEC24 | Component of the Sec23p-Sec24p heterodimer of the COPII vesicle coat, required for cargo selection during vesicle formation in ER to Golgi transport; homologous to Sfb2p and Sfb3p |
| scf184473.g15 | An16g03320 | YHR098c | SFB3 | Component of the Sec23p-Sfb3p heterodimer of the COPII vesicle coat, required for cargo selection during vesicle formation in ER to Golgi transport; homologous to Sec24p and Sfb2p |
| scf184863.g14 | An15g01520 | YPL085w | SEC16 | COPII vesicle coat protein required for ER transport vesicle budding; Sec16p is bound to the periphery of ER membranes and may act to stabilize initial COPII complexes |
|  |  |  |  |  |
| **Proteins involved in vesicle formation and docking** | | | | |
| **SNARE proteins** | | | | |
| scf185002.g61 | An12g01190 | YPL232w | SSO1/SSO2 | Plasma membrane t-SNARE involved in fusion of secretory vesicles at the plasma membrane and in vesicle fusion during sporulation; forms a complex with Sec9p that binds v-SNARE Snc2p |
| scf185002.g28 | An12g01190* | YPL232w | SSO1/SSO2 | Plasma membrane t-SNARE involved in fusion of secretory vesicles at the plasma membrane and in vesicle fusion during sporulation; forms a complex with Sec9p that binds v-SNARE Snc2p |
| scf185039.g6 | An07g02170 | YLR078c | BOS1 | V-SNARE (vesicle specific SNAP receptor), localized to the endoplasmic reticulum membrane and necessary for vesicular transport from the ER to the Golgi |
| scf185007.g149 | An08g02460 | YHL031c | GOS1 | v-SNARE protein involved in Golgi transport |
| scf184911.g2 | An02g05390 | YGR009c | SEC9 | T-SNARE protein important for fusion of secretory vesicles with the plasma membrane |
| No hits found | An07g09960 | YIL004c | BET1 | Type II membrane protein required for vesicular transport between the endoplasmic reticulum and Golgi complex; v-SNARE with similarity to synaptobrevins |
| No hits found | An15g01380 | YLR268w | SEC22 | -SNARE protein; assembles into SNARE complex with Bet1p, Bos1p and Sed5p; cycles between the ER and Golgi complex; involved in anterograde and retrograde transport between the ER and Golgi |
| scf184994.g13 | An04g07020 | YOL018c | TLG2 | Syntaxin-like t-SNARE that forms a complex with Tlg1p and Vti1p and mediates fusion of endosome-derived vesicles with the late Golgi; |
| No hits found | An04g01530 | YOR036w | PEP12 | Target membrane receptor (t-SNARE) for vesicular intermediates traveling between the Golgi apparatus and the vacuole |
| No hits found | An04g05980 | YMR197c | VTI1 | Protein involved in cis-Golgi membrane traffic; v-SNARE that interacts with two t-SNARES, Sed5p and Pep12p; required for multiple vacuolar sorting pathways |
| scf184569.g72 | An09g04890 | YDR468c | TLG1 | Essential t-SNARE that forms a complex with Tlg2p and Vti1p and mediates fusion of endosome-derived vesicles with the late Golgi; binds the docking complex VFT (Vps fifty-three) through interaction with Vps51p |
| scf185016.g92 | An04g08480 | YKL196c | YKT6 | Vesicle membrane protein (v-SNARE) with acyltransferase activity; involved in trafficking to and within the Golgi, endocytic trafficking to the vacuole, and vacuolar fusion; membrane localization due to prenylation at the carboxy-terminus |
| scf184806.g16 | An08g07470 | YLR093c | NYV1 | v-SNARE component of the vacuolar SNARE complex involved in vesicle fusion; inhibits ATP-dependent Ca(2+) transport activity of Pmc1p in the vacuolar membrane ([3](http://www.yeastgenome.org/cgi-bin/locus.fpl?locus=YLR093c#S000041180#S000041180), [4](http://www.yeastgenome.org/cgi-bin/locus.fpl?locus=YLR093c#S000062034#S000062034) and see [*Summary Paragraph*](http://www.yeastgenome.org/cgi-bin/locus.fpl?locus=YLR093c#summaryParagraph#summaryParagraph)) |
| scf184806.g6 | An12g07570 | YAL030w | SNC1 | Vesicle membrane receptor protein (v-SNARE) involved in the fusion between Golgi-derived secretory vesicles with the plasma membrane; proposed to be involved in endocytosis; member of the synaptobrevin/VAMP family of R-type v-SNARE proteins |
| No hits found | An07g05990 | YGL212w | VAM7 | Vacuolar SNARE protein that functions with Vam3p in vacuolar protein trafficking; has an N-terminal PX domain (phosphoinositide-binding module) that binds PtdIns-3-P and mediates membrane binding; SNAP-25 homolog |
| scf185013.g106 | An02g12980 | YLR026c | SED5 | cis-Golgi t-SNARE syntaxin required for vesicular transport between the ER and the Golgi complex |
| No hits found | An04g01530 | YOR106w | VAM3 | Syntaxin-like vacuolar t-SNARE that functions with Vam7p in vacuolar protein trafficking; mediates docking/fusion of late transport intermediates with the vacuole; has an acidic di-leucine sorting signal and C-terminal transmembrane region |
| scf185007.g166 | An02g06780 | YPR032w | SRO7 | Effector of Rab GTPase Sec4p, forms a complex with Sec4p and t-SNARE Sec9p; involved in exocytosis and docking and fusion of post-Golgi vesicles with plasma membrane; homolog of Sro77p and Drosophila lgl tumor suppressor |
|  |  |  |  |  |
| **Secretion related GTPases and interacting proteins** | | | | |
| scf185001.g27 | An14g00010 | YFL005w | SEC4 | Rab family GTPase essential for vesicle-mediated exocytic secretion and autophagy; |
| scf184998.g22 | An14g00010* | YFL005w | SEC4 | Rab family GTPase essential for vesicle-mediated exocytic secretion and autophagy |
| scf184798.g95 | An09g06790 | YFL038c | YPT1 | Rab family GTPase, involved in the ER-to-Golgi step of the secretory pathway |
| scf184940.g51 | An09g06790* | YFL038c | YPT1 | Rab family GTPase, involved in the ER-to-Golgi step of the secretory pathway |
| scf185000.g56 | An01g06060 | YER031c | YPT31/32 | Rab family GTPase, very similar to Ypt32p; involved in the exocytic pathway; mediates intra-Golgi traffic or the budding of post-Golgi vesicles from the trans-Golgi |
| scf185014.g102 | An15g05740 | YLR262c | YPT6 | Rab family GTPase, Ras-like GTP binding protein involved in the secretory pathway, required for fusion of endosome-derived vesicles with the late Golgi, |
| scf185007.g191 | An04g02470 | YOR089c | YPT51/52/53 | Rab family GTPase required for endocytic transport and for sorting of vacuolar hydrolases; |
| scf184977.g98 | An18g02210 | YML001w | YPT7 | Rab family GTPase; GTP-binding protein of the rab family; required for homotypic fusion event in vacuole inheritance, for endosome-endosome fusion, similar to mammalian Rab7 |
| scf184803.g25 | An18g02210* | YML001w | YPT7 | Rab family GTPase; GTP-binding protein of the rab family; required for homotypic fusion event in vacuole inheritance, for endosome-endosome fusion, similar to mammalian Rab7 |
| scf184977.g48 | An02g06400 | YOR089c | YPT51/52/53 | Rab family GTPase required for endocytic transport and for sorting of vacuolar hydrolases; |
| scf184798.g95* | An07g10340 | YER031c | YPT31 | Rab family GTPase, very similar to Ypt32p; involved in the exocytic pathway; mediates intra-Golgi traffic or the budding of post-Golgi vesicles from the trans-Golgi |
| scf184652.g57 | An14g02260 | YKR014c | YPT52 | Rab family GTPase required for endocytic transport and for sorting of vacuolar hydrolases; |
| scf185000.g24 | An02g10450 | YKR001c | VPS1 | Dynamin-like GTPase required for vacuolar sorting; also involved in actin cytoskeleton organization, endocytosis, late Golgi-retention of some proteins, regulation of peroxisome |
| scf184790.g16 | An11g09910 | YNL272c | SEC2 | Guanyl-nucleotide exchange factor for the small G-protein Sec4p |
| scf185007.g252 | An02g03120 | YER136w | GDI1 | GDP dissociation inhibitor, regulates vesicle traffic in secretory pathways by regulating the dissociation of GDP from the Sec4/Ypt/rab family of GTP binding proteins |
| scf185002.g129 | An01g04040 | YPL218w | SARA | GTPase, GTP-binding protein of the ARF family, component of COPII coat of vesicles; required for transport vesicle formation during ER to Golgi protein transport |
| scf185042.g116 | An02g07780 | YOR094w | ARF3 | Glucose-repressible ADP-ribosylation factor, GTPase of the Ras superfamily involved in development of polarity; also has mRNA binding activity |
| scf184908.g162 | An08g03690 An11g01790 | YDL137w YDL192w | ARF1  ARF2 | ADP-ribosylation factor, GTPase of the Ras superfamily involved in regulation of coated formation vesicles in intracellular trafficking within the Golgi; functionally interchangeable with Arf1p |
| No hits found | An17g00400 | YDL226c | GCS1 | ADP-ribosylation factor GTPase activating protein (ARF GAP), involved in ER-Golgi transport |
| scf184798.g65 | An18g02490 | YEL022w | GEA2 | Guanine nucleotide exchange factor for ADP ribosylation factors (ARFs), involved in vesicular transport between the Golgi and ER, |
|  |  |  |  |  |
| **ER to Golgi and Intra-Golgi transport** | | | | |
| No hits found | An11g02650 | YIL044c | AGE2 | ADP-ribosylation factor (ARF) GTPase activating protein (GAP) effector, involved in Trans-Golgi-Network (TGN) transport |
| scf184829.g71 | An08g04350 | YHR142w | CHS7 | involved in chitin biosynthesis by regulating Chs3p export from the ER |
| scf184470.g26 | An08g03590 | YGL200c | EMP24 | Component of the p24 complex; binds to GPI anchor proteins and mediates their efficient transport from the ER to the Golgi; |
| scf184761.g8 | An09g05490 | YDL018c | ERP3 | Protein with similarity to Emp24p and Erv25p, member of the p24 family involved in ER to Golgi transport |
| scf184977.g160 | An07g09160 | YGL054c | ERV14 | Protein localized to COPII-coated vesicles, involved in vesicle formation and incorporation of specific secretory cargo |
| scf184844.g135 | An01g08870* | YML012w | ERV25 | Protein that forms a heterotrimeric complex with Erp1, Erp2p, and Emp24, member of the p24 family involved in endoplasmic reticulum to Golgi transport |
| scf184921.g7 | An08g03960 | YGR284c | ERV29 | Protein localized to COPII-coated vesicles, involved in vesicle formation and incorporation of specific secretory cargo |
| scf185031.g6 | An03g04940 | YML067c | ERV41 | Protein localized to COPII-coated vesicles, forms a complex with Erv46p; involved in the membrane fusion stage of transport; |
| scf184973.g7 | An01g04320 | YAL042w | ERV46 | Protein localized to COPII-coated vesicles, forms a complex with Erv41p; involved in the membrane fusion stage of transport |
| scf184748.g5 | An18g05740 | YHR108w | GGA2 | Protein that interacts with and regulates Arf1p and Arf2p in a GTP-dependent manner to facilitate traffic through the late Golgi; |
| scf184974.g4 | An02g02830 | YCL001w | RER1 | Protein involved in retention of membrane proteins, including Sec12p, in the ER; localized to Golgi; functions as a retrieval receptor in returning membrane proteins to the ER |
| scf184908.g122 | An07g02190 | YDR170c | SEC7 | Guanine nucleotide exchange factor (GEF) for ADP ribosylation factors involved in proliferation of the Golgi, intra-Golgi transport and ER-to-Golgi transport |
| scf184911.g98 | An02g01580 | YBL050w | SEC17 | Peripheral membrane protein required for vesicular transport between ER and Golgi |
| scf184851.g26 | An09g06840 | YOR307c | SLY41 | Protein involved in ER-to-Golgi transport |
| scf184888.g15 | An08g06780 | YDL058w | USO1 | Essential protein involved in the vesicle-mediated ER to Golgi transport step |
| scf184911.g48 | An04g04950 | YLL040c | VPS13 | Protein of unknown function involved in sporulation, vacuolar protein sorting, prospore membrane formation and protein-Golgi retention |
| scf185042.g105 | An14g00210 | YGR172c | YIP1 | Integral membrane protein required for the biogenesis of ER-derived COPII transport vesicles |
| scf184656.g7 | An18g06440 | YNL044w | YIP3 | Protein localized to COPII vesicles, proposed to be involved in ER to Golgi transport; interacts with members of the Rab GTPase family and Yip1p |
| No hits found | An01g08465 | YER074w-a | YOS1 | Integral membrane protein required for ER to Golgi transport; localized to the Golgi, the ER, and COPII |
|  |  |  |  |  |
| **Golgi to endosome transport** | | | | |
| scf184999.g45 | An08g01410 | YBL102w | SFT2 | similar to mammalian syntaxin |
| scf184970.g40 | An07g08220 | YJR125c | ENT3 | Protein containing an N-terminal epsin-like domain involved in clathrin recruitment and traffic between the Golgi and endosomes |
| No hits found | An16g03420 | YJL004c | SYS1 | Integral membrane protein of the Golgi required for targeting of the Arf-like GTPase Arl3p to the Golgi |
|  |  |  |  |  |
| **Vacuolar protein sortin**g | | | | |
| scf184895.g10 | An02g06960 | YDR080w | VPS41 | Vacuolar membrane protein that is a subunit of the homotypic vacuole fusion and vacuole protein sorting |
| scf184973.g6 | An15g00460 | YGL095c | VPS45 | Protein of the Sec1p/Munc-18 family, essential for vacuolar protein sorting; required for the function of Pep12p and the early endosome/late Golgi SNARE Tlg2p; essential for fusion of Golgi-derived vesicles with the prevacuolar compartment |
| scf184766.g20 | An14g03790 | YDR164c | SEC1 | Sm-like protein involved in docking and fusion of exocytic vesicles; binds to assembled SNARE complexes at the membrane and stimulates membrane |
| scf184920.g34 | An16g03010 | YPR173c | VPS4 | AAA-ATPase involved in multivesicular body (MVB) protein sorting |
| scf185008.g12 | An02g01390 | YDR425w | SNX41 | Sorting nexin, involved in the retrieval of late-Golgi SNAREs from the post-Golgi endosome to the trans-Golgi network; interacts with Snx4p |
| scf185007.g93 | An02g05380 | YLR396c | VPS33 | ATP-binding protein that is a subunit of the HOPS complex and of the CORVET tethering complex; essential for protein sorting, vesicle docking and fusion at the vacuole |
| scf184920.g25 | An11g04400 | YNR006w | VPS27 | Endosomal protein that forms a complex with Hse1p; required for recycling Golgi proteins |
| scf184583.g15 | An14g05130 | YPL045w | VPS16 | Subunit of the vacuole fusion and protein sorting HOPS complex and the CORVET tethering complex |
| scf184844.g15 | An15g00540 | YOL129w | VPS68 | Vacuolar membrane protein of unknown function involved in vacuolar |
| scf184938.g54 | An01g08400 | YOR069w | VPS5 | Nexin-1 homolog required for localizing membrane proteins from a prevacuolar/late endosomal compartment back to the late Golgi apparatus |
| scf184970.g11 | An01g04080 | OR359w | VTS1 | shows genetic interactions with Vti1p, a v-SNARE involved in cis-Golgi membrane traffic |
| scf184783.g13 | An01g10830 | YPL065w | VPS28 | Component of the ESCRT-I complex (Stp22p, Srn2p, Vps28p, and Mvb12p |
| scf185042.g41 | An01g04550 | YAL002w | VPS8 | Membrane-binding component of the CORVET complex; involved in endosomal vesicle tethering and fusion in the endosome to vacuole protein targeting pathway; interacts with |
| scf185002.g79 | An02g09460 | YDR323c | PEP7 | Multivalent adaptor protein that facilitates vesicle-mediated vacuolar protein sorting by ensuring high-fidelity vesicle docking and fusion |
| scf184606.g2 | An08g01030 | YHR012w | VPS29 | Endosomal protein that is a subunit of the membrane-associated retromer complex essential for endosome-to-Golgi retrograde transport |
| scf185008.g79 | An01g07920 | YML097c | VPS9 | A guanine nucleotide exchange factor involved in vesicle-mediated vacuolar protein transport; specifically stimulates the intrinsic guanine nucleotide exchange activity of Vps21p/Rab5 |
|  |  |  |  |  |
| **Cellular export and secretion** | | | | |
| scf184759.g1 | An02g08450 | YBR080c | SEC18 | ATPase required for vesicular transport between ER and Golgi |
| scf184813.g9 | An02g14450 | YGL167c | PMR1 | High affinity Ca2+/Mn2+ P-type ATPase required for Ca2+ and Mn2+ transport into Golgi |
| scf185014.g104 | An01g08530 | YNL238w | KEX2 | Subtilisin-like protease (proprotein convertase), a calcium-dependent serine protease involved in the activation of proproteins of the secretory pathway |
|  |  |  |  |  |
