# Supplementary material for: The genome of the white-rot fungus Pycnoporus cinnabarinus: a basidiomycete model with a versatile arsenal for lignocellulosic biomass breakdown
Source: BMC Genomics. 2014 Jun 18;15:486. doi: 10.1186/1471-2164-15-486 (PMC4101180; doi:10.1186/1471-2164-15-486)
Supplement: Supplementary file 19 — Additional file 19: Table S11: P. cinnabarinus genome annotation related to protein glycosylation pathways. (DOCX 32 KB) [file 12864_2014_6245_MOESM19_ESM.docx]

**Additional file 19: Table S11. *P. cinnabarinus* genome annotation related to protein glycosylation pathways**

| **Biosynthesis of nucleotide sugars for glycosylation events** | | | | |
| --- | --- | --- | --- | --- |
| **UDP-Glucose** | | | | |
| scf185043.g193 | An12g08610 | YCL040w | GLK1 | Glucokinase, catalyzes the phosphorylation of glucose at C6 |
| scf184901.g5 | An02g14380  An15g05940*  An16g01620*  An06g00380*  An13g00510* | YFR053c | HXK1 | Hexokinase, catalyzes phosphorylation of glucose |
| scf184845.g15 | An02g07650 | YMR105c | PGM2 | Phosphoglucomutase, catalyzes the conversion from glucose-1-phosphate to glucose-6-phosphate |
| scf185032.g4 | An07g06780 | YMR278w | PGM3 | Phosphoglucomutase, catalyzes interconversion of glucose-1-phosphate and glucose-6-phospate; |
| scf184976.g14  scf184665.g5* | An12g00820 | YKL035w | UGP1 | UDP-glucose pyrophosphorylase, catalyses the reversible formation of UDP-Glc from glucose 1-phosphate and UTP |
| scf184992.g24 | An16g05420 | YBR196c | PGI1 | Phosphoglucose isomerase, catalyzes the interconversion of glucose-6-phosphate and fructose-6-phosphate |
|  |  |  |  |  |
| **UDP-N-acetyl-glucosamine** | | | | |
| scf184790.g17 | An18g06820  An03g05940* | YKL104c | GFA1 | Glutamine-fructose-6-phosphate amidotransferase, catalyzes the formation of glucosamine-6-P and glutamate from fructose-6-P and glutamine. |
| scf184806.g26 | An12g07840 | YFL017c | GNA1 | Glucosamine-6-phosphate acetyltransferase; involved in UDP-N-acetylglucosamine synthesis, forms GlcNAc6P from AcCoA |
| scf184911.g99 | An18g05160 | YEL058w | PCM1 | N-acetylglucosamine-phosphate mutase; converts GlcNAc-6-P to GlcNAc-1-P. |
| scf185007.g57 | An12g00480 | YDL103c | UAP1 | UDP-N-acetylglucosamine pyrophosphorylase, catalyzes the formation of UDP-N-acetylglucosamine (UDP-GlcNAc). |
|  |  |  |  |  |
| **GDP-mannose** | | | | |
| scf184934.g4  scf185008.g128 | An04g03200 An08g06350 | YER003c | PMI40 | Mannose-6-phosphate isomerase, catalyzes the interconversion of fructose-6-P and mannose-6-P; |
| scf184951.g21 | An18g06500 | YFL045c | SEC53 | Phosphomannomutase, involved in synthesis of GDP-mannose and dolichol-phosphate-mannose |
| scf185032.g4 | An07g06780 | YMR278w | PGM3 | Phosphoglucomutase, catalyzes interconversion of glucose-1-phosphate and glucose-6-phospate |
| No hits found (e-value < e-10) | An04g04990 | YDL055c | PSA1 | GDP-mannose pyrophosphorylase (mannose-1-phosphate guanyltransferase), synthesizes GDP-mannose from GTP and mannose-1-phosphate |
| No hits found (e-value < e-10) | An11g02380 | YDL055c | PSA1 | GDP-mannose pyrophosphorylase (mannose-1-phosphate guanyltransferase), synthesizes GDP-mannose from GTP and mannose-1-phosphate |
|  |  |  |  |  |
| **UDP-galactose** | | | | |
| scf184979.g64 | An16g04160 | YBR020w | GAL1 | Galactokinase, phosphorylates alpha-D-galactose to alpha-D-galactose-1-phosphate |
| scf184636.g6 | An02g03590 | YBR018c | GAL7 | Galactose-1-phosphate uridyl transferase, synthesizes glucose-1-phosphate and UDP-galactose from UDP-D-glucose and alpha-D-galactose-1-phosphate |
| scf184817.g2 | An02g09090 | YBR019c | GAL10 | UDP-glucose-4-epimerase, catalyzes the interconversion of UDP-galactose and UDP-D-glucose in galactose metabolism |
| scf184753.g44 | An14g03820 | YBR019c | GAL10 | UDP-glucose-4-epimerase, catalyzes the interconversion of UDP-galactose and UDP-D-glucose in galactose metabolism |
| scf185043.g176 | An16g00180 | YBR019c | GAL10 | UDP-glucose-4-epimerase, catalyzes the interconversion of UDP-galactose and UDP-D-glucose in galactose metabolism |
| scf184979.g52 | An01g11440 | - | - | Strong similarity to UDP-glucose-4-epimerase |
| scf184817.g2 | An02g09090 | YHR210c | - | sequence similarity to aldose 1-epimerases such as GAL10 |
| No hits found (e-value < e-10) | An02g08660 | - | - | UDP-galactopyranose mutase |
| No hits found (e-value < e-10) | An16g02380 | - | - | UDP-galactopyranose mutase |
|  |  |  |  |  |
| **Transporters of sugar nucleotide donors** | | | | |
| **GDP-mannose** | | | | |
| scf184969.g4 | An17g02140 | YGL225w | VRG4 | Golgi GDP-mannose transporter |
| scf185042.g161 | An11g02020 | YML038c | YMD8 | Putative nucleotide sugar transporter, similar to Vrg4 |
|  |  |  |  |  |
| **UDP-GlcNac** | | | | |
| scf184969.g10 | An03g06940 | YEL004w | YEA4 | Uridine diphosphate-N-acetylglucosamine transporter required for cell wall chitin synthesis |
|  |  |  |  |  |
| **UDP-galactose** | | | | |
| scf184799.g26  scf185014.g127 | An08g10400 | - | - | Strong similarity to UDP-Gal transporter |
| scf184649.g22 | An18g04260 | YPL244c | HUT1 | Protein with a role in UDP-galactose transport to the Golgi lumen |
|  |  |  |  |  |
| **UDP-galactofuranose** | | | | |
| No hits found (e-value < e-10) | An02g08670 | - | - | Putative UDP-galactofuranose transporter |
|  |  |  |  |  |
| **Oligosaccharyltransferase subunits** | | | | |
| scf184908.g89 | An02g14560 | YJL002c | OST1 | Subunit of the OST complex of the ER lumen |
| scf184569.g15 | An04g03495 | YMR149w | SWP1 | Subunit of the OST complex of the ER lumen |
| scf184940.g5 | An07g04190 | YEL002c | WBP1 | Subunit of the OST complex of the ER lumen |
| scf185043.g188 | An18g03920 | YOR103c | OST2 | Subunit of the OST complex of the ER lumen |
| scf184863.g13 | An02g14930 | YOR085w | OST3 | Subunit of the OST complex of the ER lumen |
| scf184805.g41 | An16g08570 | YGL022w | SST3 | Subunit of the OST complex of the ER lumen |
| No hits found (e-value < e-10) | An08g07485 | YDL232W | OST4 | Subunit of the OST complex of the ER lumen |
|  |  |  |  |  |
| **Synthesis of the dolicholphosphate linked ER-precursor Glc3Man9GlcNAc2** | | | | |
| scf184931.g2 | An04g03960 | YMR013c | SEC59 | Dolichol kinase, catalyzes the terminal step in dolichyl monophosphate (Dol-P) biosynthesis |
| scf184806.g20 | An16g04330 | YPR183w | DPM1 | Dol-P-Man synthase of the ER membrane, catalyzes the formation of Dol-P-Man from Dol-P and GDP-Man |
| No hits found (e-value < e-10) | An01g05200 | - | - | Dolichol phosphate-mannose biosynthesis regulatory |
| No hits found (e-value < e-10) | An14g00270 | - | - | Dolichol-phosphate mannosyltransferase subunit 3 |
| No hits found (e-value < e-10) | An03g04410 | YPL227c | ALG5 | UDP-glucose:dolichyl-phosphate glucosyltransferase |
| scf184801.g52 | An02g03240 | YBR243c | ALG7 | UDP-N-acetyl-glucosamine-1-P transferase, transfers Glc-Nac-P from UDP-GlcNac to Dol-P |
| scf184785.g46 | An01g09110 | YGL047w | ALG13 | Catalytic component of UDP-GlcNAc transferase, required for the second step of dolichyl-linked oligosaccharide synthesis; |
| scf185001.g38 | An06g01100 | YBR110w | ALG1 | Mannosyltransferase, involved in asparagine-linked glycosylation in the endoplasmic reticulum |
| scf184940.g36 | An14g05910 | YGL065c | ALG2 | Mannosyltransferase that catalyzes two consecutive steps in the N-linked glycosylation pathway |
| scf184970.g62 | An18g05910 | YNL048w | ALG11 | Alpha-1,2-mannosyltransferase, catalyzes sequential addition of the two terminal alpha 1,2-mannose residues |
|  |  | YBL020w | RFT1 | translocation of Man5GlcNac2-PP-Dol from the cytoplasmic side to the lumenal side of the ER membrane but is not the flippase |
| scf184801.g4 | An04g03130 | - | - | Strong similarity to flippase = flipping the precursor Oligosaccharide |
| scf185002.g48 | An18g02360 | YBL082c | ALG3 | Dolichol-P-Man dependent alpha(1-3) mannosyltransferase |
| scf185007.g234 | scf184940.g74 | An02g14940 | ALG9 | both the transfer of seventh mannose residue on B-arm and ninth mannose residue on the C-arm from Dol-P-Man to lipid-linked oligosaccharides |
| scf184939.g28 | An01g08460 | YNR030w | ALG12 | Alpha-1,6-mannosyltransferase localized to the ER; responsible for the addition of the alpha-1,6 mannose to dolichol-linked Man7GlcNAc2 |
|  |  |  |  |  |
| scf184909.g11 | An02g12630 | YOR002w | ALG6 | Alpha 1,3 glucosyltransferase, involved in transfer of oligosaccharides from dolichyl pyrophosphate to asparagine |
| scf184652.g33 | An04g08820 | YOR067c | ALG8 | Glucosyl transferase, involved in N-linked glycosylation; adds glucose to the dolichol-linked oligosaccharide precursor prior to transfer to protein during lipid-linked oligosaccharide biosynthesis; similar to Alg6p |
| scf184672.g13 | An02g02980 | YGR227w | ALG10 | Dolichyl-phosphoglucose-dependent alpha-1,2 glucosyltransferase of the ER, functions in the pathway that synthesizes the dolichol-linked oligosaccharide precursor for N-linked protein glycosylation |
| scf184857.g35* | An15g01460 | YGR036C | CWH8 | Dolichyl pyrophosphate (Dol-P-P) phosphatase |
|  |  |  |  |  |
| **Processing of the ER-precursor Glc3Man9GlcNAc2 after transfer to a polypeptide** | | | | |
| scf184750.g6 | An15g01420 | YGL027c | CWH41 | Processing alpha glucosidase I, ER type II |
| scf184985.g33 | An09g05880 | YBR229c | ROT2 | Glucosidase II catalytic subunit |
| scf184940.g83 | An01g10930 | YBR229c | ROT2 | Glucosidase II catalytic subunit |
| scf185022.g7 | An04g06920 | YBR229c | ROT2 | Glucosidase II catalytic subunit |
| scf184603.g6 | An04g06920* | YBR229c | ROT2 | Glucosidase II catalytic subunit |
| scf184798.g2 | An01g04880 | YBR229c | ROT2 | Glucosidase II catalytic subunit |
| scf185043.g220 | An13g00620 | YDR221w | GTB1 | Glucosidase II beta subunit, |
| scf184763.g4 | An18g06220 | YJR131w | MNS1 | α-1,2-mannosidase involved in ERAD |
| scf184815.g42 | An04g06990 | YJR131w | MNS1 | α-1,2-mannosidase involved in ERAD |
| scf184815.g58 | An04g06990* | YJR131w | MNS1 | α-1,2-mannosidase involved in ERAD |
| scf184569.g13 | An01g12550 | YJR131w | MNS1 | α-1,2-mannosidase involved in ERAD |
| No bidirectional best hit | An06g01510 | YLR057w | MNL2 | Putative mannosidase involved in ERAD |
| scf185043.g124 | An12g00340 | YHR204w | MNL1 | Alpha-1,2-specific exomannosidase of the endoplasmic reticulum |
|  |  |  |  |  |
| **Golgi mannosyltransferase** | | | | |
| No hits found (e-value < e-10) | An03g01090 An05g01750 An05g02320 An11g07490 An12g07020 An14g07140 | YJR075w | HOC1 | α-1,6-mannosyltransferase involved in cell wall mannan biosynthesis |
| No hits found (e-value < e-10) | An07g04940 | YGL038c | OCH1 | Mannosyltransferase of the cis-Golgi apparatus, initiates the mannose outer chain elongation of N-linked oligosaccharides |
| No hits found (e-value < e-10) | An03g05010 | YPL050c | MNN9 | Subunit of the alpha-1,6 mannosyltransferase complex |
| No hits found (e-value < e-10) | An04g01260 | YEL036c | ANP1 | Subunit of the alpha-1,6 mannosyltransferase complex |
| No hits found (e-value < e-10) | An15g06230 An15g03330 | YDR245w | MNN10 | Subunit of the alpha-1,6 mannosyltransferase complex |
| No hits found (e-value < e-10) | An04g05940 | YJL183w | MNN11 | Subunit of the alpha-1,6 mannosyltransferase complex |
| No hits found (e-value < e-10) | An04g06730 An14g06060 An15g00920 | YBR015c | MNN2 | α-1,2-mannosyltransferase, responsible for addition of the first α- -1,2-linked mannose |
|  |  |  |  |  |
| **O-Glycosylation in ER** | | | | |
| scf184588.g3 | An11g09890 | YDL095w | PMT1 | Protein O-mannosyltransferase, transfers mannose residues from dolichyl phosphate-D-mannose to protein Ser/Thr residues; |
| scf184996.g57 | An07g10350 | YAL023c  YOR321w | PMT2  PMT3 | Protein O-mannosyltransferase, transfers mannose residues from dolichyl phosphate-D-mannose to protein Ser/Thr residues; |
| scf184938.g56 | An16g08490 | YJR143c | PMT4 | Protein O-mannosyltransferase, transfers mannose residues from dolichyl phosphate-D-mannose to protein Ser/Thr residues; |
|  |  |  |  |  |
| **Other genes possibly involved in glycosylation events** | | | | |
| scf185033.g56  scf184863.g22*  scf184943.g72* | An14g03910 | YOR099w | KRE2family | Alpha-1,2-mannosyltransferase involved in O- and N-linked protein glycosylation |
| No hits found (e-value < e-10) | An18g03940 An08g05380 | YKL201c | MNN4 | Putative positive regulator of mannosylphosphate transferase (Mnn6p), involved in mannosylphosphorylation of N-linked oligosaccharides |
| No hits found (e-value < e-10) | An15g04810 | YIL014w | MNT3 | Alpha-1,3-mannosyltransferase, adds the fourth and fifth alpha-1,3-linked mannose residues to O-linked |
| No hits found (e-value < e-10) | An03g02990 An11g10260 | YOR320c | GNT1 | N-acetylglucosaminyltransferase capable of modification of N-linked glycans in the Golgi apparatus |
|  |  |  |  |  |
| **Other putative alpha-1,2-mannosidase (no homology to the MNS1/ER-alpha-1,2-mannosidase family** | | | | |
| scf184969.g71 | An08g03060 | - | - | putative alpha-1,2-mannosidase |
| scf184501.g5 | An08g03060* | - | - | putative alpha-1,2-mannosidase |
| scf184969.g74 | An13g01260* | - | - | putative alpha-1,2-mannosidase |
| scf184817.g11 | An14g04240* | - | - | putative alpha-1,2-mannosidase |
| scf184817.g13 | An14g04240* | - | - | putative alpha-1,2-mannosidase |
| scf184590.g13 | An02g11720 | YGL156w | AMS1 | Vacuolar alpha mannosidase, involved in free oligosaccharide (fos) degradation |
|  |  |  |  |  |
| **GPI ANCHOR BIOSYNTHESIS** | | | | |
| scf184996.g52 | An16g03530 | YGR216C | GPI1 | Protein involved in the synthesis of GlcNAc-PI |
| No hits found (e-value < e-10) | An02g13570 | YPL076W | GPI2 | Protein involved in the synthesis of GlcNAc-PI |
| scf184693.g7 | An01g09910 | YPL175W | GPI3 | Protein involved in the synthesis of GlcNAc-PI |
| No hits found (e-value < e-10) (e-value < e-10) | An02g09230 | YNL038W | GPI15 | Protein involved in the synthesis of GlcNAc-PI |
| scf184830.g9 | An16g01530 | YDR437W | GPI19 | Protein involved in the synthesis of GlcNAc-PI |
| scf184569.g56 | An14g06640 | YMR281W | GPI12 | Protein involved in the second step of GPI anchor assembly, the de-N-acetylation of the N-acetylglucosaminyl-phosphatidylinositol |
| scf184969.g82 | An01g12990 | YJL091C | GWT1 | Protein involved in the inositol acylation of glucosaminyl phosphatidylinositol (GlcN-PI) to form glucosaminyl(acyl)phosphatidylinositol (GlcN(acyl)PI |
| scf185002.g128 | An12g01880 | YJR013W | GPI14 | GPI-alpha-1,4 mannosyltransferase I |
| No hits found (e-value < e-10) | An17g00780 | YCL052C | PBN1 | Essential component of GPI-mannosyltransferase I, |
| scf184836.g39 | An14g00900 | YKL165C | MCD4 | multimembrane-spanning protein that localizes to the ER |
| scf184830.g9 | An16g01530 |  | GPI18 | mannosyltransferase that transfers the second mannose in glycosylphosphatidylinositol biosynthesis |
| 69scf185014.g36 | An04g04110 | YGL142C | GPI10 | putative alpha 1,2 mannosyltransferase required for addition of the third mannose onto the GPI core structure |
| No hits found (e-value < e-10) | An10g00480 | YKL165C | GPI11 | involved in the addition of phosphoethanolamine to the multiply mannosylated GPI intermediate |
| scf185033.g18 | An09g02800  An04g05100 | YLL031C | GPI13 | Phosphoryltransferase that adds phosphoethanolamine onto the third mannose residue of the GPI-anchor precursor |
|  |  |  |  |  |
| **GPI-anchor transamidase complex; removes the GPI-Anchoring signal and attaches GPI to proteins in the ER** | | | | |
| No hit found | An08g10720 | YLR088W | GAA1 | Subunit of the GPI (glycosylphosphatidylinositol):protein transamidase complex; ScGaa1-like |
| scf184915.g49 | An04g02650 | YLR459W | GAB1 | Subunit of the GPI protein transamidase complex |
| scf184970.g112 | An11g06770 | YHR188C | GPI16 | Subunit of the GPI protein transamidase complex |
| scf184830.g15 | An07g09270 | YDR434W | GPI17 | Subunit of the GPI protein transamidase complex |
| scf184943.g62 | An01g13530 | YDR331W | GPI8 | Subunit of the GPI protein transamidase complex |
|  |  |  |  |  |
