# Supplementary material for: The genome of the white-rot fungus Pycnoporus cinnabarinus: a basidiomycete model with a versatile arsenal for lignocellulosic biomass breakdown
Source: BMC Genomics. 2014 Jun 18;15:486. doi: 10.1186/1471-2164-15-486 (PMC4101180; doi:10.1186/1471-2164-15-486)
Supplement: Supplementary file 20 — Additional file 20: Figure S5: Alignments of the N-terminal regions of HD1 (A) and HD2 mating type proteins (B) of Pycnoporus and other Agaricomycetes. (DOCX 182 KB) [file 12864_2014_6245_MOESM20_ESM.docx]

**
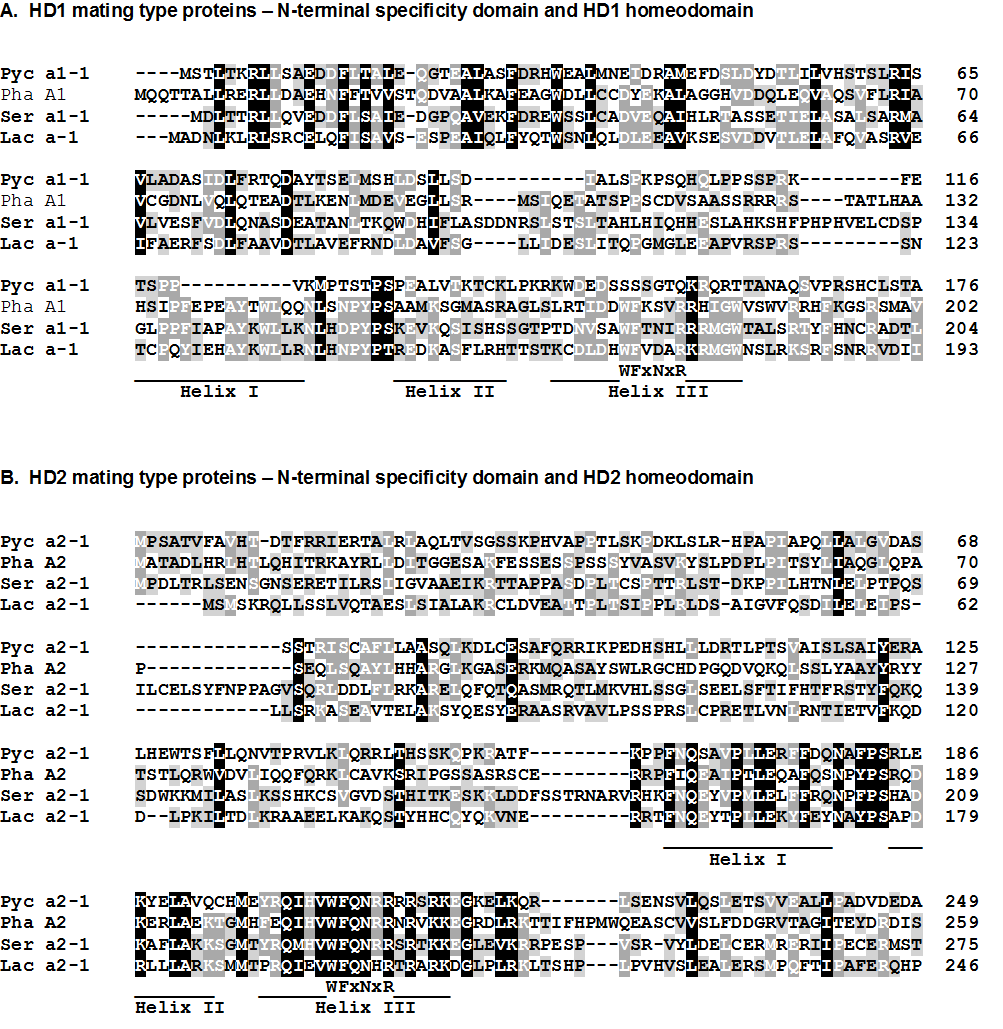
**

**Additional file 20: Figure S5. Alignments of the N-terminal regions of HD1 (A) and HD2 mating type proteins (B) of *Pycnoporus* and other Agaricomycetes.** GenBank accession numbers: *Phanerochaete chrysosporium* A1 (ADN97192.1) and A2 mating type protein (ADN97171.1); *Serpula lacrymans* a1-1 (EGO31057.1) and a2-1 (EGO31058.1); JGI IDs (http://genome.jgi-sf.org/Lacbi2/Lacbi2.home.html): *Laccaria bicolor* a1-1 (ID301103) and a2.1 (ID379291). The three helical positions in the homeodomains and the invariant amino acids in the DNA-binding-motif are marked.
