# Supplementary material for: The genome of the white-rot fungus Pycnoporus cinnabarinus: a basidiomycete model with a versatile arsenal for lignocellulosic biomass breakdown
Source: BMC Genomics. 2014 Jun 18;15:486. doi: 10.1186/1471-2164-15-486 (PMC4101180; doi:10.1186/1471-2164-15-486)
Supplement: Supplementary file 21 — Additional file 21: Figure S6: Alignment of sequences of putative G protein-coupled transmembrane pheromone receptors deduced from EST contigs of P. cinnabarinus (Pci) and sequences of pheromone receptors of P. chrysosporium (Pch; for nomenclature, see James et al. [117]). (DOCX 2 MB) [file 12864_2014_6245_MOESM21_ESM.docx]

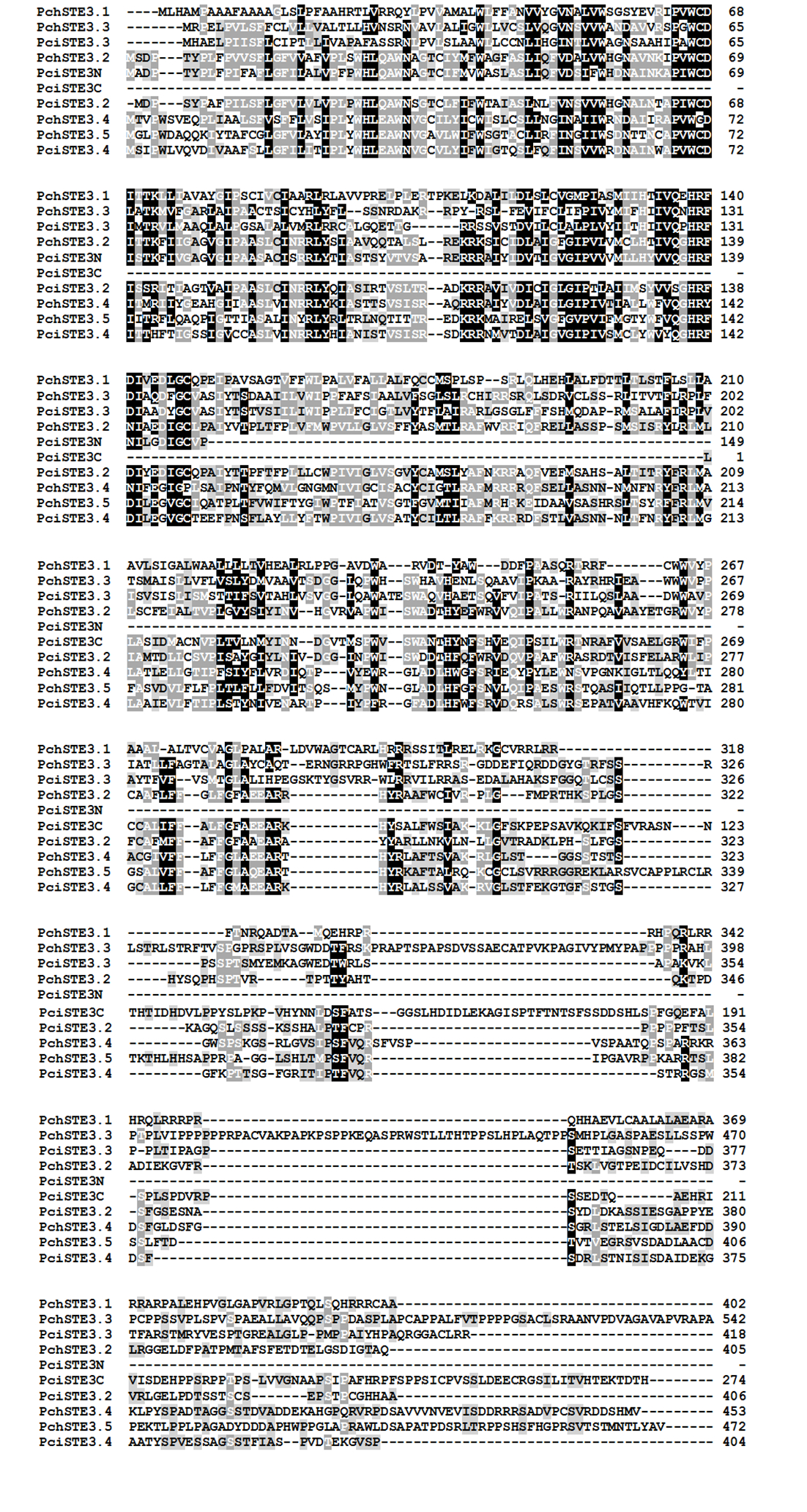


**Additional file 21: Figure S6. Alignment of sequences of putative G protein-coupled transmembrane pheromone receptors deduced from EST contigs of *P. cinnabarinus* (Pci) and of pheromone receptors of *P. chrysosporium*** (Pch; for nomenclature see James et al. [117]). PciSTE3.2, PciSTE3.3, PciSTE3N (only an N-terminal half), PciSTE3C (only a C-terminal half), PciSTE3.4 (nearly complete) were deduced from EST contigs GCTO4WP02F2FHA.f.pc.1, GCTO4WP02F02LK.f.pc.1, GCTO4WP02FNFO2.f.pc.1, GCTO4WP02F7KNS.f.pc.1, and GCTO4WP02F082I.f.pc.1, respectively.
