# Supplementary material for: The genome of the white-rot fungus Pycnoporus cinnabarinus: a basidiomycete model with a versatile arsenal for lignocellulosic biomass breakdown
Source: BMC Genomics. 2014 Jun 18;15:486. doi: 10.1186/1471-2164-15-486 (PMC4101180; doi:10.1186/1471-2164-15-486)
Supplement: Supplementary file 22 — Additional file 22: Figure S7: Neighbour-joining trees (bootstrap values: 500) of sequences of A. (nearly) complete, B. N-terminal and C. C-terminal halves of pheromone receptors of P. cinnabarinus and P. chrysosporium (for nomenclature see James et al. [117]). The classification in non-mating type and B orthologs follows the analysis of Niculita-Hierzel et al. [115]. (DOCX 38 KB) [file 12864_2014_6245_MOESM22_ESM.docx]

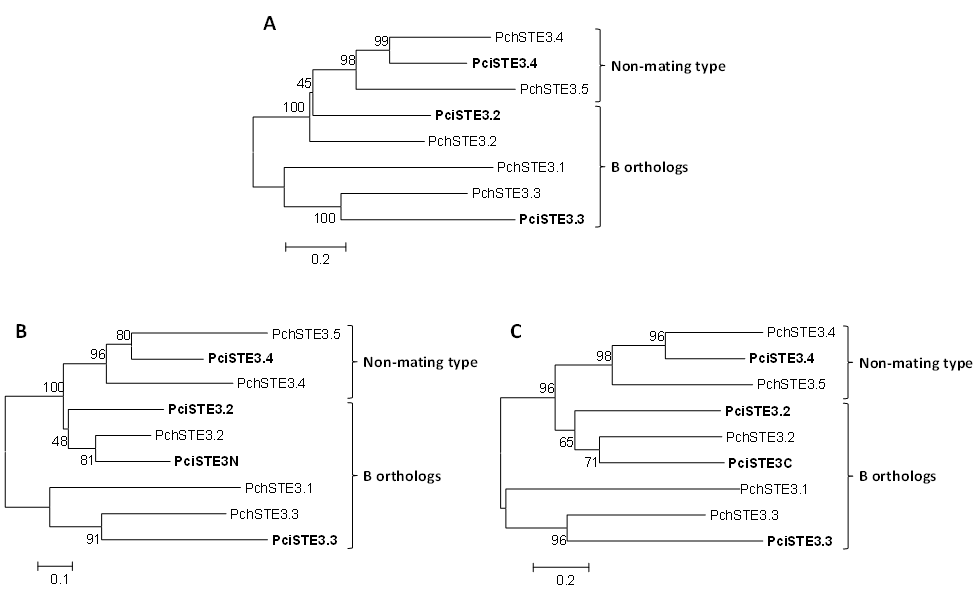


**Additional file 22: Figure S7. Neighbour-joining trees (bootstrap values: 500) of sequences of A. (nearly) complete, B. N-terminal and C. C-terminal halves of pheromone receptors of *P. cinnabarinus* and *P. chrysosporium*** (for nomenclature see James et al. [117]). The classification in non-mating type and B orthologs follows the analysis of Niculita-Hierzel et al. [115].
