# Supplementary material for: The genome of the white-rot fungus Pycnoporus cinnabarinus: a basidiomycete model with a versatile arsenal for lignocellulosic biomass breakdown
Source: BMC Genomics. 2014 Jun 18;15:486. doi: 10.1186/1471-2164-15-486 (PMC4101180; doi:10.1186/1471-2164-15-486)
Supplement: Supplementary file 23 — Additional file 23: Figure S8: Alignment of sequences of putative B mating type pheromone precursors (Ph1 and Ph2) and of putative precursors for non-mating-type pheromone-like peptides (Phl1 to Phl3). (DOCX 42 KB) [file 12864_2014_6245_MOESM23_ESM.docx]

**
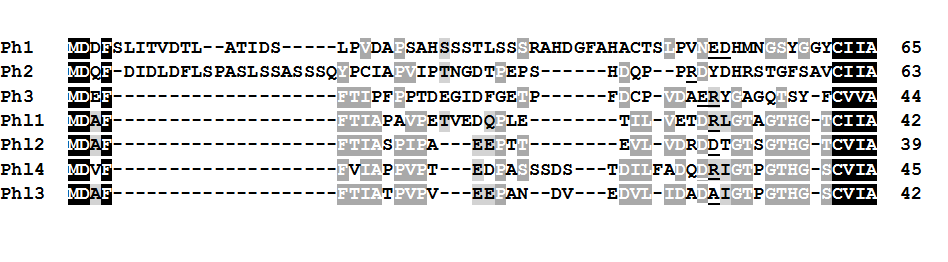
**

**Additional file 23: Figure S8.** **Alignment of sequences of putative B mating type pheromone precursors (Ph1 and Ph2) and of putative precursors for non-mating-type pheromone-like peptides (Phl1 to Phl3).** The N-termini have a conserved MDA/Df-mtif and the C-terminus the CAAX-motif for pheromone processing. Underlined are charged dipeptides at positions typical for peptide processing [111]. The precursors are encoded on EST contigs GCTO4WP02F39ZZ.f.pc.1, GCTO4WP02F04V1.f.pc.1, GCTO4WP02F1UJP.f.pc.1, GCTO4WP02F1MB5.f.pc.1, GCTO4WP02F67UZ.f.pc.1, GCTO4WP02F07I6.f.pc.1, and GCTO4WP02F2RWG.f.pc.1, respectively.
